# Consideration of inequalities in effectiveness trials of mHealth applications – a systematic assessment of studies from an umbrella review

**DOI:** 10.1186/s12939-024-02267-4

**Published:** 2024-09-11

**Authors:** Nancy Abdelmalak, Jacob Burns, Laura Suhlrie, Michael Laxy, Anna-Janina Stephan

**Affiliations:** Professorship of Public Health and Prevention, TUM School of Medicine and Health, Technical University of Munich, Munich, Germany

**Keywords:** mHealth, Equity, Equality, Social determinants of health, Diabetes mellitus, Hypertension, PROGRESS-Plus, Systematic review

## Abstract

**Background:**

The growing use of mobile health applications (apps) for managing diabetes and hypertension entails an increased need to understand their effectiveness among different population groups. It is unclear if efficacy and effectiveness trials currently provide evidence of differential effectiveness, and if they do, a summary of such evidence is missing. Our study identified to what extent sociocultural and socioeconomic inequalities were considered in effectiveness trials of mobile health apps in diabetic and hypertensive patients and if these inequalities moderated app effectiveness.

**Methods:**

We built on our recent umbrella review that synthesized systematic reviews (SRs) of randomized controlled trials (RCTs) on the effectiveness of health apps. Using standard SR methodologies, we identified and assessed all primary RCTs from these SRs that focused on diabetes and/or hypertension and reported on health-related outcomes and inequality-related characteristics across intervention arms. We used the PROGRESS-Plus framework to define inequality-related characteristics that affect health opportunities and outcomes. We used harvest plots to summarize the subgroups (stratified analyses or interaction terms) on moderating effects of PROGRESS-Plus. We assessed study quality using the Risk of Bias 2 tool.

**Results:**

We included 72 published articles of 65 unique RCTs. Gender, age, and education were the most frequently described PROGRESS-Plus characteristics at baseline in more than half of the studies. Ethnicity and occupation followed in 21 and 15 RCTs, respectively. Seven trials investigated the moderating effect of age, gender or ethnicity on app effectiveness through subgroup analyses. Results were equivocal and covered a heterogenous set of outcomes. Results showed some concerns for a high risk of bias, mostly because participants could not be blinded to their intervention allocation.

**Conclusions:**

Besides frequently available gender, age, and education descriptives, other relevant sociocultural or socioeconomic characteristics were neither sufficiently reported nor analyzed. We encourage researchers to investigate how these characteristics moderate the effectiveness of health apps to better understand how effect heterogeneity for apps across different sociocultural or socioeconomic groups affects inequalities, to support more equitable management of non-communicable diseases in increasingly digitalized systems.

**Registration:**

https://osf.io/89dhy/.

**Supplementary Information:**

The online version contains supplementary material available at 10.1186/s12939-024-02267-4.

## Background

The increasing use of mobile health (mHealth) applications (apps) entails a growing focus on evidence regarding their effectiveness. Several systematic reviews (SRs) suggest that using mHealth apps may be effective in chronic disease management through improving medication adherence, physical functioning, and other health outcomes [1, 2]. Such evidence reinforces ongoing policy efforts to integrate mHealth apps as a delivery channel of healthcare services [3]. A concrete example is Germany’s recently established Digital Health Applications (DiGA) system, which allows doctors to prescribe apps through the public health insurance scheme to complement standard disease treatment and management [4]. However, it remains unclear if mHealth app effectiveness generalizes equally to different population subgroups with heterogenous sociocultural and socioeconomic characteristics. With health equity recognized as one crucial performance indicator for health systems [5], political decision-makers should ask if mHealth app integration into standard care can be expected to increase or alleviate existing health inequalities and health inequities [6] and be ready to take measures to mitigate unintended equity consequences, if needed.

Diabetes and hypertension are two highly prevalent and socially unequally distributed chronic conditions [7] for which evidence of the overall effectiveness of mHealth apps exists [8, 9]. In 2019, diabetes contributed to 70.9 million (2.8%) Disability Adjusted Life years (DALYs) globally [10] and, in 2020, was ranked ninth by the World Health Organization (WHO) in the leading causes of death [11]. Hypertension, the s*ilent killer* [12], is a significant risk factor for cardiac diseases and the leading risk factor in hypertensive heart diseases [13, 14].

Patients can self-manage both diseases with proper monitoring, education, and awareness, making mHealth interventions promising support tools [15]. Therefore, integrating digital health technologies into standard diabetes and hypertension care could enhance equity and socio-economic inclusion [16, 17]. Unlike conventional in-person healthcare, using mHealth for disease management and treatment may come with less commuting and waiting time, fewer physical and transportation obstacles to access healthcare, particularly serving people with disabilities, and lower commuting costs, mainly serving people of low socio-economic status [17].

The epidemiology of diabetes and hypertension is already strongly associated with social determinants of health (SDOH); both are more prevalent and entail more and worse complications in disadvantaged communities [18, 19]. Relevant predictors of increased diabetes risk are low socioeconomic position (SEP) (expressed in education, income, or occupation), racism, deprived neighborhoods (housing conditions), and detrimental food access and food insecurity [18]. Diabetes also lowers and negatively affects the engagement of patients in the labor market [20]. Similarly, ethnicity, discrimination, racism, and lower education have been linked to worsened hypertension [19]. Lack of health insurance can additionally limit healthcare access and thereby limit monitoring and controlling of these diseases [18, 19, 21, 22]. Diabetic and hypertensive patients are at a higher risk of suffering a disability [20], limiting their access to healthcare and increasing their risk of suffering discrimination and becoming socially vulnerable [23].

Three types of digital divide could materialize if already deprived groups additionally encounter less access to (first), usage of (second), and benefits from usage of (third) digital health technology [24]. Although declining, there are still variations in (affordable) access to the internet between different regions, countries, and cities [25, 26]. Even if equal access is provided to internet and health technologies, take-up and utilization patterns differ among socially more and less disadvantaged groups [17, 24]. For example, digital exclusion is more manifested among individuals of older age, with a disability, with low income, with low education, or living in rural areas [17]. Lastly, even in the presence of equal access and take-up, the amount of benefit an individual can realize through using digital health technologies may depend on social factors. For example, in the Grossman model education increases marginal productivity of inputs (e.g. information communicated mHealth apps) into health production (suggesting potentially higher health benefits from usage in individuals with higher education or health literacy) [27]. On the other hand, the availability and accessibility of (in-person) health services in urban areas may result in reduced in marginal benefit from additional usage of health technologies compared to rural areas where such technologies may not act as an adjunct, but in some instances as the only available healthcare delivery channel.

As a consequence, mHealth apps have the potential to reinforce or reduce existing health inequalities. Therefore, scientific evidence on differential app effectiveness (inequalities in benefit) is a prerequisite for political decision-makers attempting to improve or ensure equity through political measures.

In this systematic assessment, we explored to what extent effect heterogeneity across sociocultural and socioeconomic characteristics that might increase or decrease health inequalities is considered in trials investigating the effectiveness of mHealth apps on health outcomes in patients with diabetes and/or hypertension. Firstly, we identified and summarized which factors representing inequalities were reported in these studies, either descriptively or through subgroup analyses. Secondly, we assessed, summarized, and synthesized how and to what extent these inequality factors moderate the effectiveness of health apps.

## Methods

Our study design, which we refer to as a systematic assessment, is largely akin to a SR. Our only deviation from the traditional SR methods relates to how we identified potential eligible studies, i.e. the search strategy. While a traditional SR generally conducts primary searches within electronic databases to identify potentially eligible studies, we followed a previously described approach [28] and used the included studies of existing and related SRs as the pool of potentially eligible studies. We identified these relevant systematic reviews through the conduct of our recently published umbrella review [29], which identified and mapped available SRs of RCTs on mHealth app effectiveness across various disease indications. This systematic assessment approach follows the work of others and emphasizes efficiency and the responsible use of research resources (i.e. building on rather than duplicating existing research). More detail on how these potentially eligible studies were identified, as well as the other methods, are described in detail below after the “Study Inclusion and Exclusion Criteria”.

We followed a pre-defined publicly available protocol [30] and existing methodological guidelines for conducting and reporting SRs, including the Preferred Reporting Items for Systematic Reviews and Meta-Analyses - Equity (PRISMA-E) extension [31] (Additional file1).

### Study inclusion and exclusion criteria

Our eligibility criteria (Additional file2) were based on the population (patients with diabetes and/or hypertension), intervention (mHealth app), comparator (any), and study design (RCTs) (PICOS) framework [32].

Following previous studies [28, 33–35] and methodological recommendations [36], we defined inequality characteristics using the PROGRESS-Plus framework [37]. *PROGRESS* stands for place of residence, religion, occupation, gender, race/ethnicity, education, SEP, and social networks/capital, and *Plus* for age, disability, sexual orientation, and context-specific additional factors [37]. For this study, we included insurance status as a *Plus* characteristic.

### Identification of potentially eligible studies

The search from the umbrella review, which aimed to identify all SRs of RCTs on mHealth app effectiveness across various disease indications, was concluded on 28 August 2023. From these SRs, we used backward citation searches to identify all underlying primary RCTs that focused on diabetes (all types) and/or hypertension.

Where primary studies could not be identified (i.e. where a SR did not provide the full references of all included studies), we contacted the SR’s corresponding authors to obtain the references. Where contact attempts remained unsuccessful, we cross-checked the names of the primary RCT’s authors and the publication years mentioned in the SR with studies cited in other relevant SRs with the same author names and years. Where such cross-checks remained unsuccessful, we additionally used PubMed and Google Scholar to identify the full reference of the remaining studies with incomplete citations via their available author-year combinations.

### Study selection

Given the small pool of potentially eligible studies, we combined the title and abstract screening with the full-text screening stage into one screening and selection step of the full text of the studies. After deduplication, the primary reviewer (NA) screened all primary studies for eligibility. A second reviewer (LS, AJS, GM, or NOK) verified these decisions. We used Microsoft Excel [38] to facilitate this process.

### Data extraction strategy

From the selected studies, we extracted details on the population, intervention, control, outcome(s), and sample sizes at baseline for each RCT. We extracted the reported PROGRESS-Plus-related descriptives from all studies. Additionally, for RCTs that conducted subgroup analyses on PROGRESS-Plus characteristics, we extracted sample size for intention-to-treat (ITT) analyses, the type of subgroup analysis (interaction terms between the intervention and subgroup, or stratified analysis of the intervention effect within each subgroup) and the respective effect estimates. Adjusted overall effect estimates were additionally extracted for outcomes with PROGRESS-Plus subgroup analyses. The primary reviewer (NA) extracted all data, and a second reviewer (AJS, GM, or NOK) double-checked the extraction.

### Study quality assessment

We assessed the quality of studies that conducted subgroup analyses for PROGRESS-Plus characteristics. Although effect heterogeneity may not be influenced by the bias in the overall outcome/effect estimate, we aimed to understand if and how far the overall health outcomes that were included in subgroup analyses were biased. To do so, we used the Risk of Bias (RoB) 2 tool, which assesses RoB at the outcome level [39]. We based this assessment on the respective overall outcome, not on the outcome specific to the subgroup analysis.

We used separate tools for individually-randomized parallel and cluster-randomized RCTs as recommended and followed the available guidance documents to answer the signaling questions [39]. Two reviewers (NA and LS) independently assessed the RoB of all the outcomes and compared their assessments for consensus. In cases of differences, AJS and JB were available for consolidation. Additional information on the tool and decision rules supporting the assessment are outlined in Additional file3.

### Data synthesis and presentation

We narratively summarized the main characteristics of the included studies. Subsequently, we created bar charts presenting the number of studies that covered each PROGRESS-Plus characteristic in their baseline descriptives or through subgroup analyses. For each PROGRESS-Plus characteristic, we additionally summarized and compared its distribution across study samples in tables and figures. For categorical variables, we reported numbers and percentages; for continuous variables that could be presented consistently across studies, we calculated the minimum, maximum, mean, and median. Additional file4 details how we harmonized the PROGRESS-Plus descriptives across studies and analyzed their distribution.

The results of PROGRESS-Plus subgroup analyses were visualized using harvest plots as one of the methods recommended by Cochrane for synthesis without a meta-analysis (SWiM) [40, 41]. Of note, harvest plots visualize effect directions and the relative size of effect estimates without considering the statistical significance of a result [40]. For the summary of subgroup analyses, we used the phrasing of the PROGRESS-Plus levels as reported in the respective original study. The harvest plots display sections for each PROGRESS-Plus characteristic (e.g., gender), the health outcomes (e.g., HbA1c), and the months after which the outcome changed. Additional file5 further visualizes and explains the interpretation of the harvest plot.

## Results

### Identification and selection of studies

Of the 48 SRs in the umbrella review [29], 24 included at least one study on diabetes or hypertension populations. The search strategies from these 24 systematic reviews encompassed 30 databases: Medline, PubMed, Scopus, Cochrane, Central Register of Controlled Trials (CENTRAL), PsycINFO, Embase, Google Scholar, ProQuest, Web of Science, Cumulative Index to Nursing and Allied Health Literature, Latin American and Caribbean Health Sciences Literature (LILACS), China National Knowledge Infrastructure (CNKI), China biology medicine database, Wanfang, Sinomed, Cumulative Index to Nursing and Allied Health Literature (CINAHL), Koreamed, KMbase, ScienceOn, Elsevier, SAGE, Science Direct, Taylor & Francis, IEEE XPLORE, Academic Research Premier, BVSalud, EBSCO, The Joanna Briggs Institute Library, Chinese Biomedical Literature, and Clinical Trials.

Three reviews did not cite their 54 primary studies [42–44], and the corresponding authors did not reply to our respective inquiries. Yet, we could identify most studies by author and year, leaving four unidentifiable records.

**Fig. 1:**
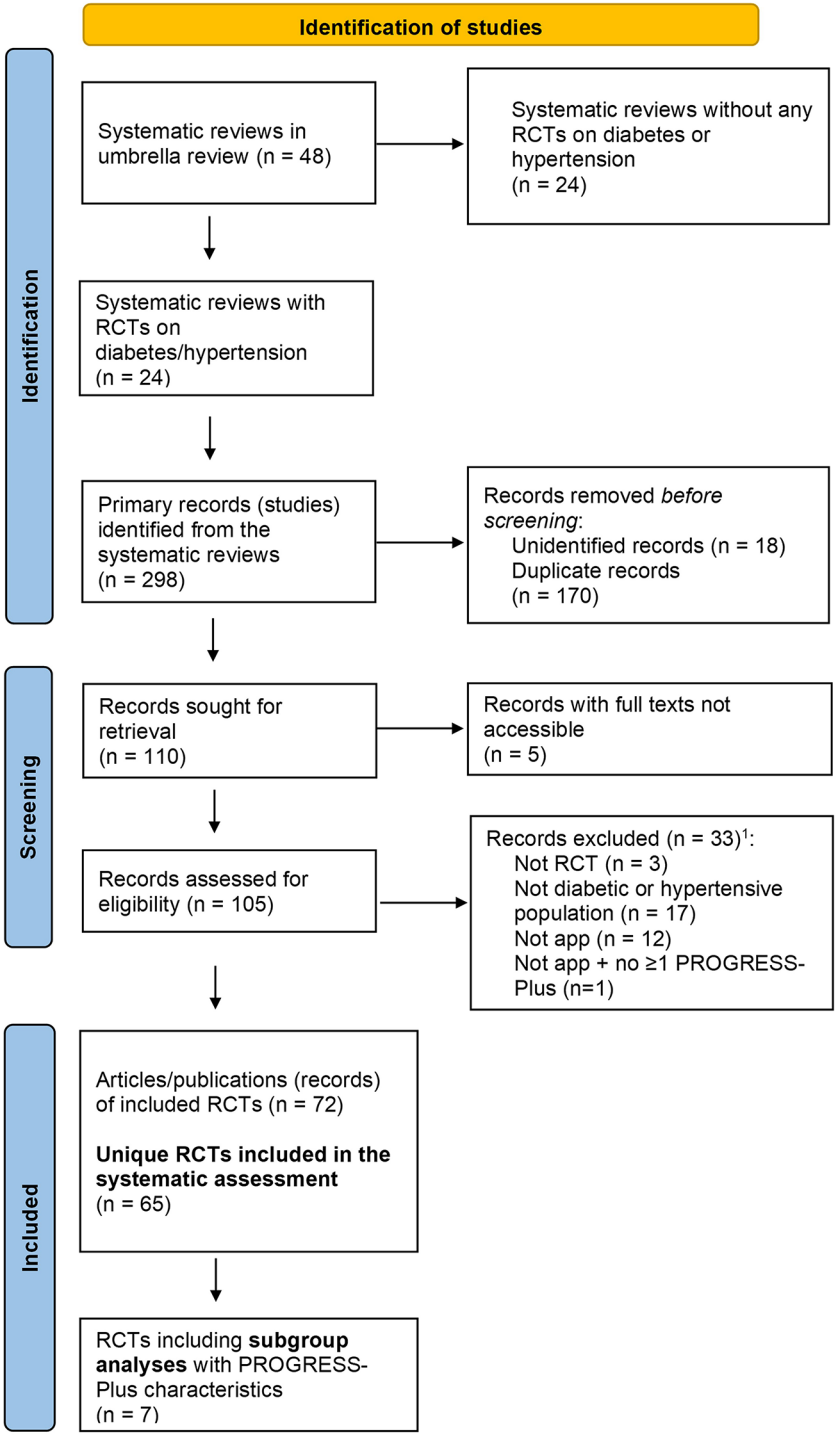
PRISMA [45] flow diagram of Identified and Included Studies. *Notes*^1^ Although excluded studies may have met more than one exclusion criterion, the screening process stopped for a study once the first exclusion criterion was identified, and only this exclusion criterion was recorded. Abbreviations app: application; RCT: randomized controlled trial

In total, we extracted 298 records from the umbrella review, of which 170 were duplicates and 18 unidentifiable records. We retrieved the remaining 110 primary studies and assessed their eligibility. This resulted in including 72 published articles which reported on 65 unique RCTs (Fig. 1).

A list of the 24 SRs and the excluded primary RCTs with reasons is provided in Additional file6.

### Characteristics of included studies

**Fig. 2:**
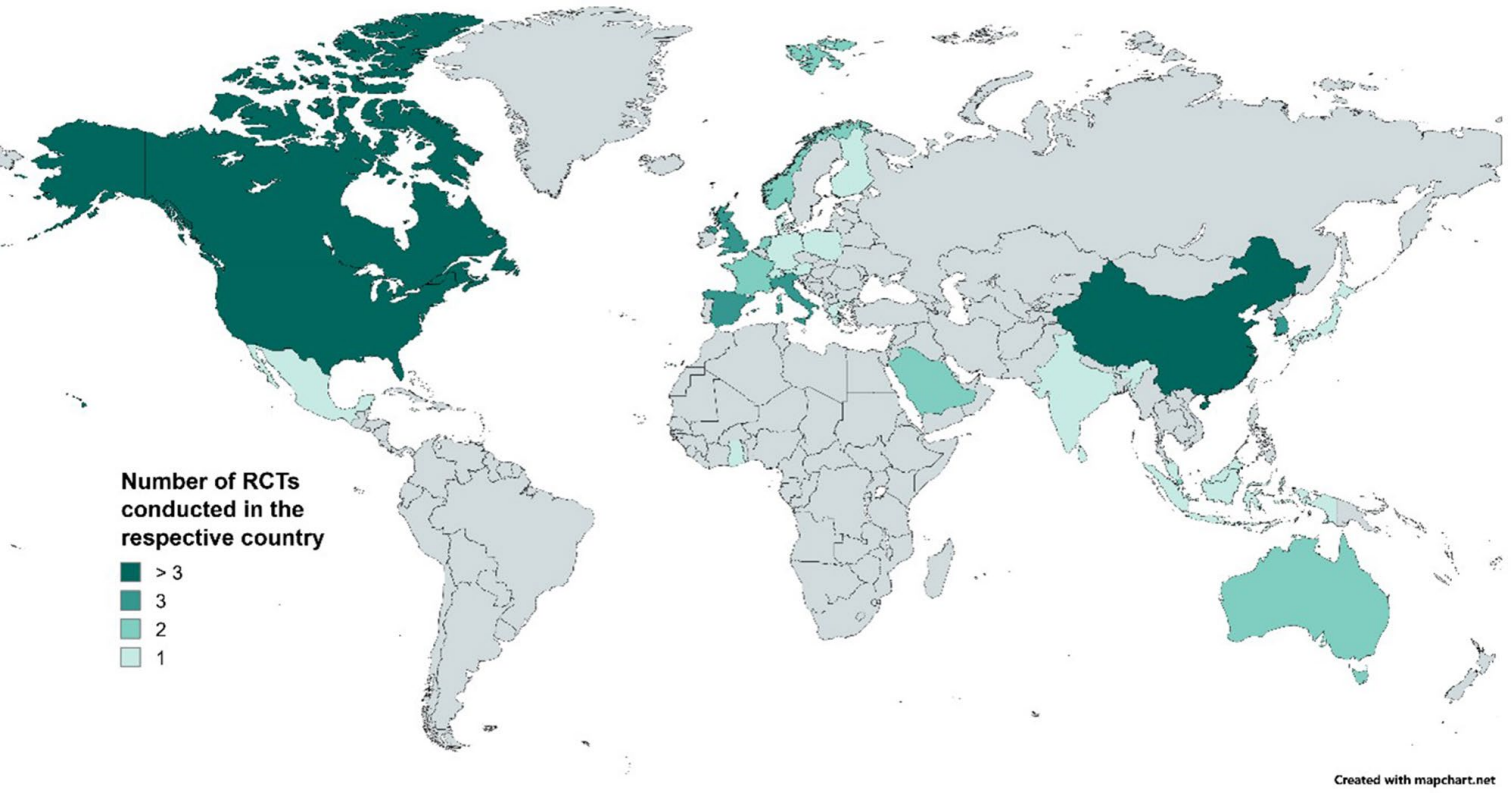
Map of the countries covered in the 66 studies, created using MapChart [54]. *Notes* United States (*n* = 14) [55–70]; China (*n* = 9) [71–79]; Canada (*n* = 5) [80–84]; United Kingdom (*n* = 4 [United Kingdom = 3 [85–88], England = 1 [89, 90]]); South Korea (*n* = 3) [91–93]; Spain (*n* = 3) [89, 90, 94–96]; Italy (*n* = 3) [89, 90, 97, 98]; Singapore (*n* = 2) [99, 100]; France (*n* = 2) [101, 102]; Norway (*n* = 2) [103–105]; Saudi Arabia (*n* = 2) [106, 107]; Netherlands (*n* = 2) [108, 109]; Australia (*n* = 2) [110, 111]; Finland (*n* = 1) [112]; Switzerland (*n* = 1) [113]; Austria (*n* = 1) [114]; Mexico (*n* = 1) [52]; Germany (*n* = 1) [115]; Sri Lanka (*n* = 1) [47]; Poland (*n* = 1) [116]; Japan (*n* = 1) [117]; Ghana (*n* = 1) [50, 51]; Taiwan (*n* = 1) [71]; Denmark (*n* = 1) [118]; Greece (*n* = 1) [119]; India (n = 1) [48]; Indonesia (*n* = 1) [49]; Malaysia (*n* = 1) [53]. Rossi et al. [89, 90] took place in England, Spain, and Italy, therefore it is counted 3 times

Included articles were published between 2009 and 2020, with most publications in 2019 (*n* = 17). The most frequent countries where the studies took place were the United States (US) (*n* = 14), China (*n* = 9), and Canada (*n* = 5). Most studies (*n* = 59) were conducted in high-income countries [46], four in lower-middle-income countries [47–51], two in upper-middle-income countries [52, 53], and none in low-income countries. Figure 2 illustrates this geographical distribution.

Out of *n* = 72 articles (65 unique RCTs with 7796 individuals, mean and median of the number of participants are 120 and 85 respectively); *n* = 58 focused on diabetes (*n* = 51 unique RCTs with 5815 individuals), *n* = 10 on hypertension (*n* = 9 unique RCTs with 1622 individuals) [50, 51, 56, 57, 63–65, 70, 72, 96] and *n* = 4 on diabetes and/or hypertension (359 individuals) [59, 73, 82, 114].

The RCTs focusing on diabetes included *n* = 14 on T1DM [60, 61, 81, 84, 89, 97, 101, 105, 109, 111, 113, 115, 118, 119], *n* = 32 on T2DM (one of which had overweight/obese patients [75]), five without a specification of diabetes type [47, 78, 79, 85, 87], and none focused on gestational diabetes. Two RCTs of the *n* = 9 on hypertension included hypertensive stroke survivors [50, 51, 63].

Diabetes studies reported on glycated hemoglobin (HbA1c), self-efficacy and self-care, biomarkers, anthropometric, behavioral, and psychological outcomes. Healthcare utilization was reported five times [66, 75, 85, 101, 118] and medication prescription changes once [66, 67]. Hypertension studies reported on blood pressure, medication adherence, consumption of sodium, and other types of food. Studies on both diseases reported on blood pressure, HbA1c, anxiety, and depression [59, 73, 82, 114]. All studies reported at least one PROGRESS-Plus descriptive and seven reported PROGRESS-Plus-relevant subgroup analyses [48, 64, 65, 68, 93, 97, 110].

Additional file7 includes a study-level summary of study designs, diseases, sample sizes, and follow-up time points. Additional file8 contains a study-level summary of the interventions and apps, their functions, the devices used, and comparison groups. Additional file9 includes a summary of the outcomes and overall effect estimates for outcomes further assessed in subgroup analyses.

### Study quality assessment

We assessed RoB for 11 outcomes included with PROGRESS-Plus subgroup analyses. Eight outcomes stem from five individually-randomized parallel RCTs (HbA1c [*n* = 3], compliance with self-monitoring of blood glucose [*n* = 1], health-related quality of life [HRQoL] [*n* = 1], medication adherence [*n* = 1], and systolic blood pressure [SBP] [*n* = 2]) [48, 64, 65, 97, 110], and three outcomes from two cluster-randomized parallel RCTs (HbA1c [*n* = 2] and fasting plasma glucose [FPG] [*n* = 1]) [68, 93]. All studies were judged as having some or a high risk of bias as participants were unblinded to their allocation. Some studies lacked information regarding blinded outcome assessors or had self-reported outcomes by the participants who were unblinded [64, 66, 68, 110]. The randomization domain had some RoB in cluster RCTs as allocation was not always clearly concealed until after recruitment [93]. Further RoB2 results and figures can be found in Additional file10.

### Reported PROGRESS-Plus characteristics

**Fig. 3:**
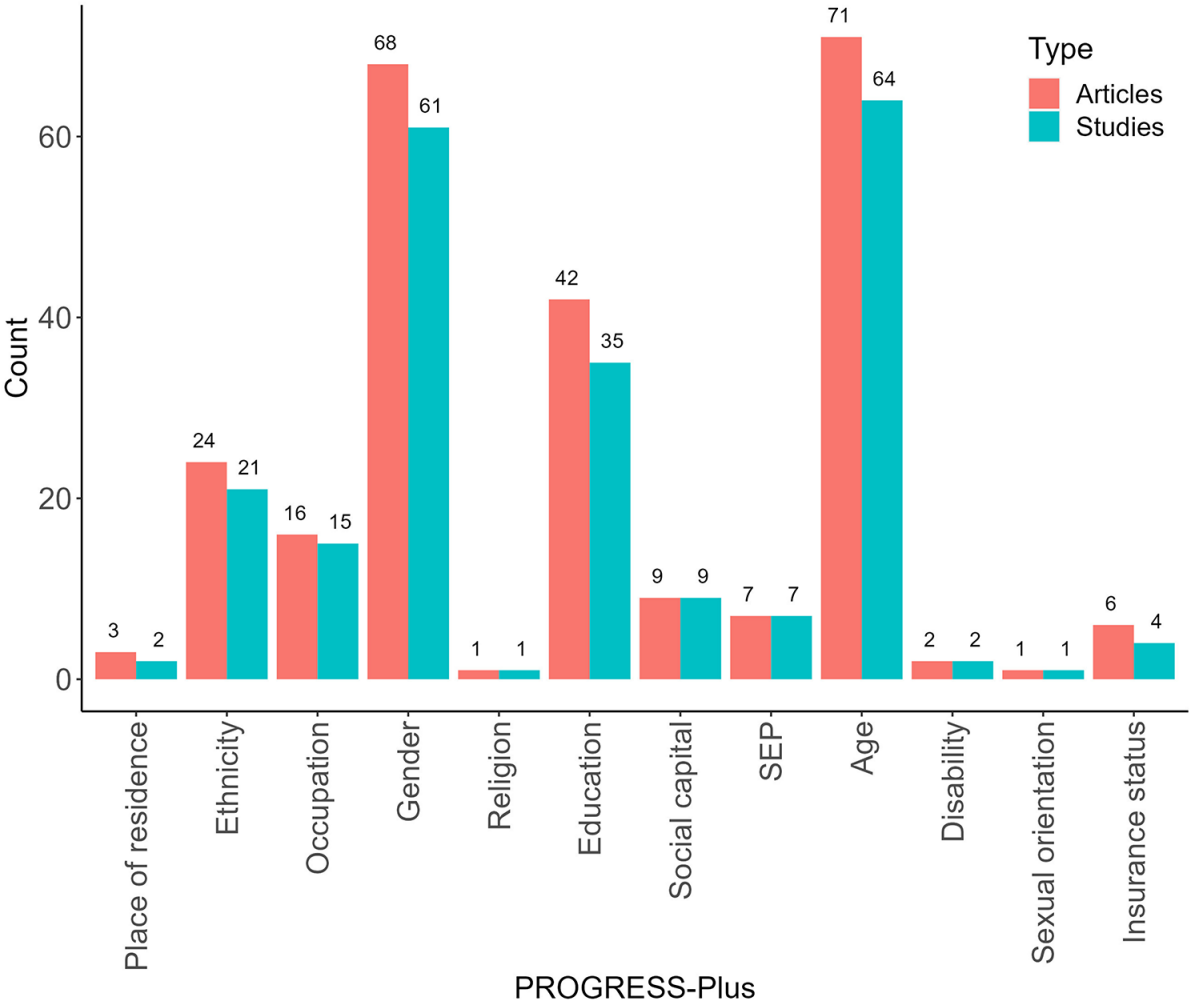
Number of unique RCTs and articles reporting on PROGRESS-Plus Characteristics. Notes Abbreviations SEP: socioeconomic position

The most frequently reported PROGRESS-Plus descriptives were age, gender, and education (Fig. 3).

One RCT reported participant shares with urban and rural (58% vs. 42% respectively) places of residence [50, 51]. Another RCT reported the living conditions of the participants; more than half had between four and five rooms (54%), while the rest had less [100].

**Fig. 4:**
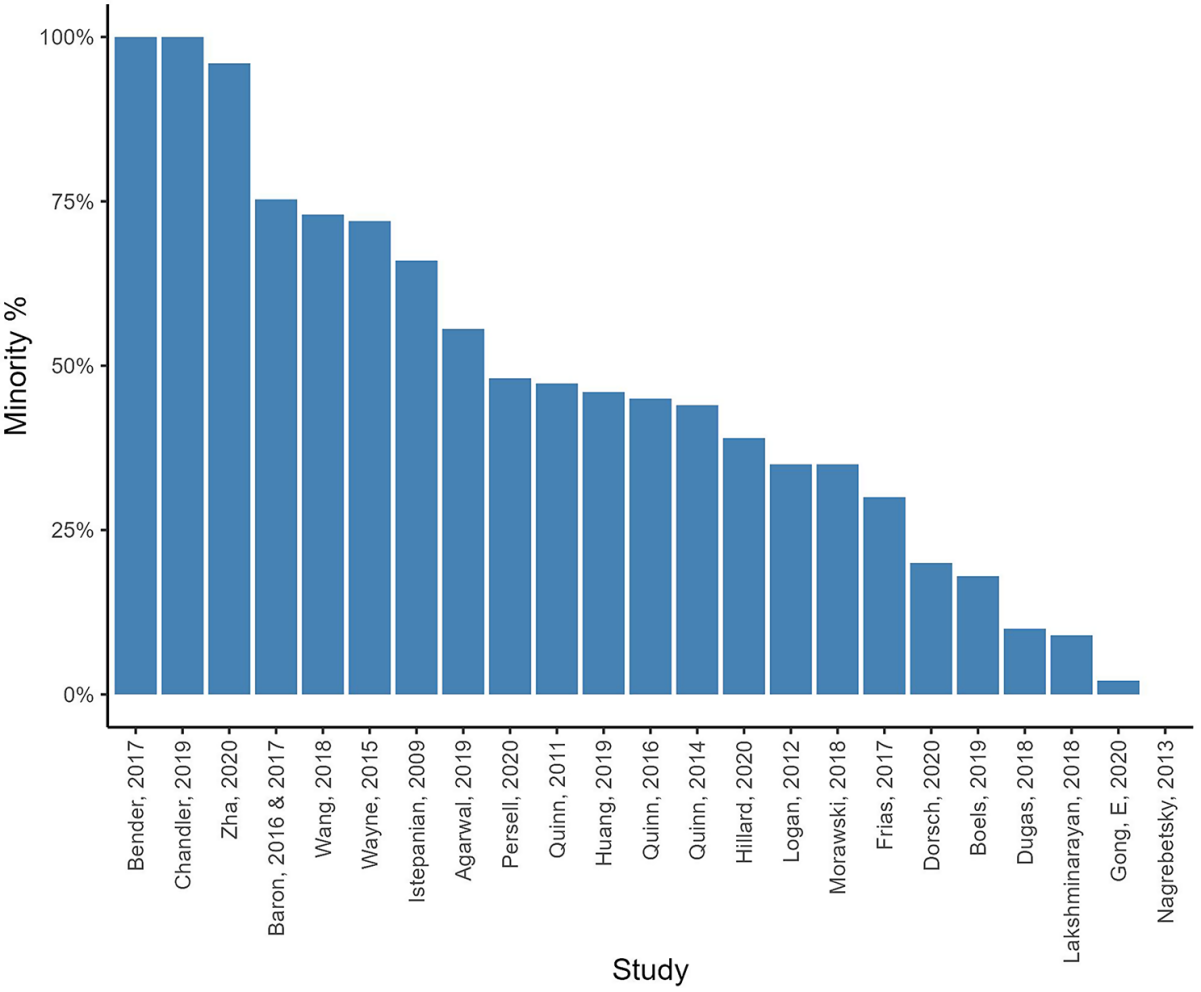
Percentages of ethnic/racial minorities in the RCTs. *Notes* Dorsch et al. [57], Frias et al. [59], Persell et al. [65], Wang et al. [69], and Zha et al. [70] reported on both ethnicity and race, and race is what is illustrated in the figure as it included more subgroups

Ethnicity and/or race descriptives were reported in *n* = 21 studies (see Table 1 in Additional file 11 for more details and descriptive analysis). Shares of participants from an ethnic minority ranged from 0% [88] to 100% [55, 56] (Fig. 4). Six RCTs explicitly targeted minority/underserved populations [55, 56, 69, 70, 83, 87]. One RCT in the US reported on the language of the participants and 48% spoke English at home [85, 86].

**Fig. 5:**
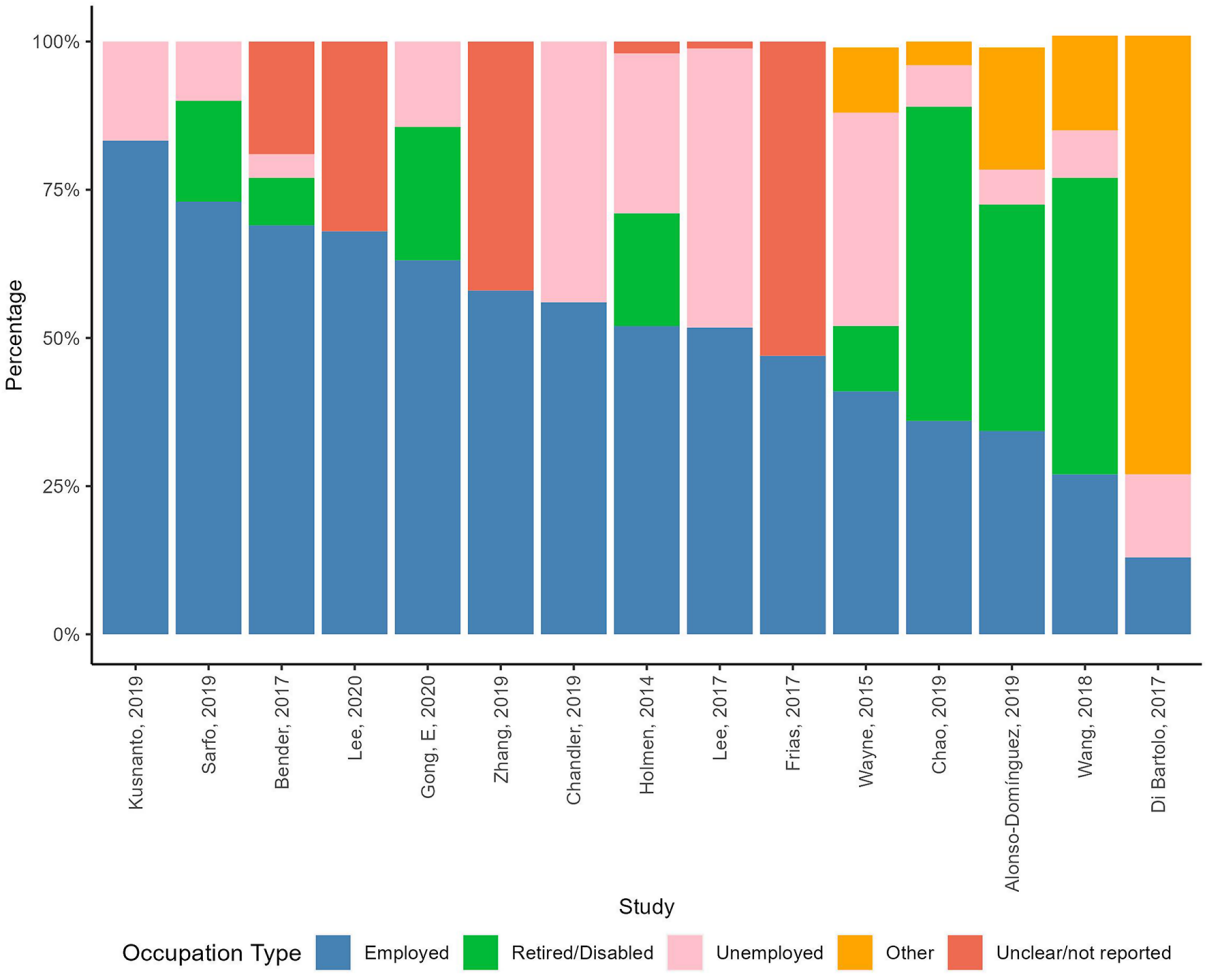
Distribution of occupational statuses in the RCTs. *Notes* Percentages might not add up to 100% due to approximation of decimal numbers. Gong et al. [110] reports 14.4% under unemployed or other combined, and the graph illustrates them under unemployed

Fifteen studies [49, 50, 53, 55, 56, 59, 69, 71, 78, 83, 92, 94, 97, 103, 110] reported on the occupational status of the participants (see Table 2 in Additional file 11 for more details and descriptive analysis). Shares of employed participants ranged from 13 to 83% (Fig. 5).

**Fig. 6:**
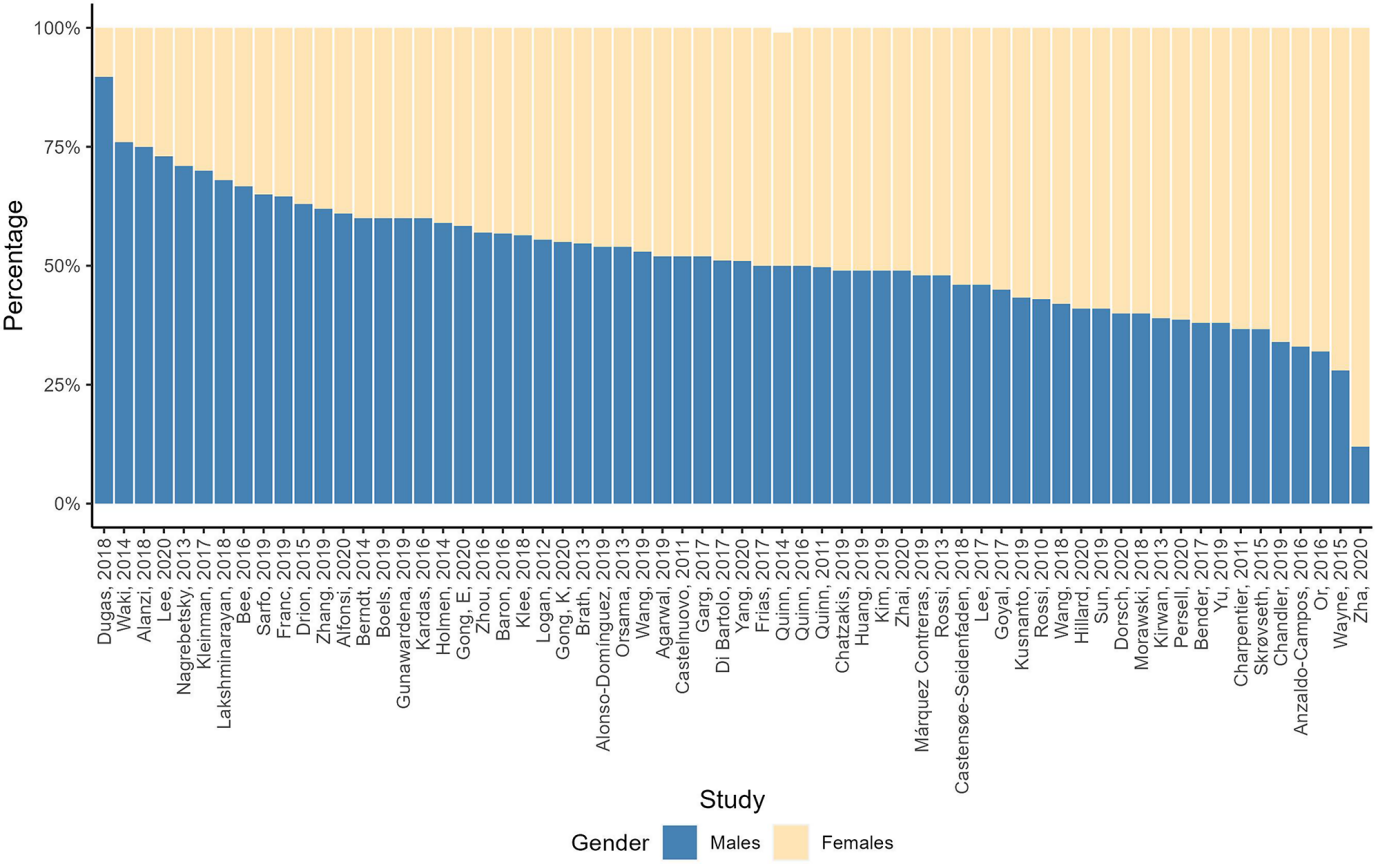
Gender percentages reported in the RCTs. *Notes* Percentages might not add up to 100% due to approximation of decimal numbers

Of the 61 studies reporting on gender (see Table 3 in Additional file 11 for more details and descriptive analysis), approximately two-thirds of studies (40 of 61) included percentages of males or females ranging between 40% and 60% (Fig. 6). The RCT with the lowest number of males was a pilot study in a community center in Newark, US, and 88% of the participants were women [70]. The study with the highest male percentage (90%) was also in the US and focused on veterans [58].

Religion was mentioned in one study from Malaysia. All participants were Muslims with diabetes who were fasting during the month of Ramadan [53].

Educational levels of participants were reported in 35 studies and were inconsistently reported across the studies (see Table 4 in Additional file 11 for more details and descriptive analysis). One study had solely highly educated participants [55].

**Fig. 7:**
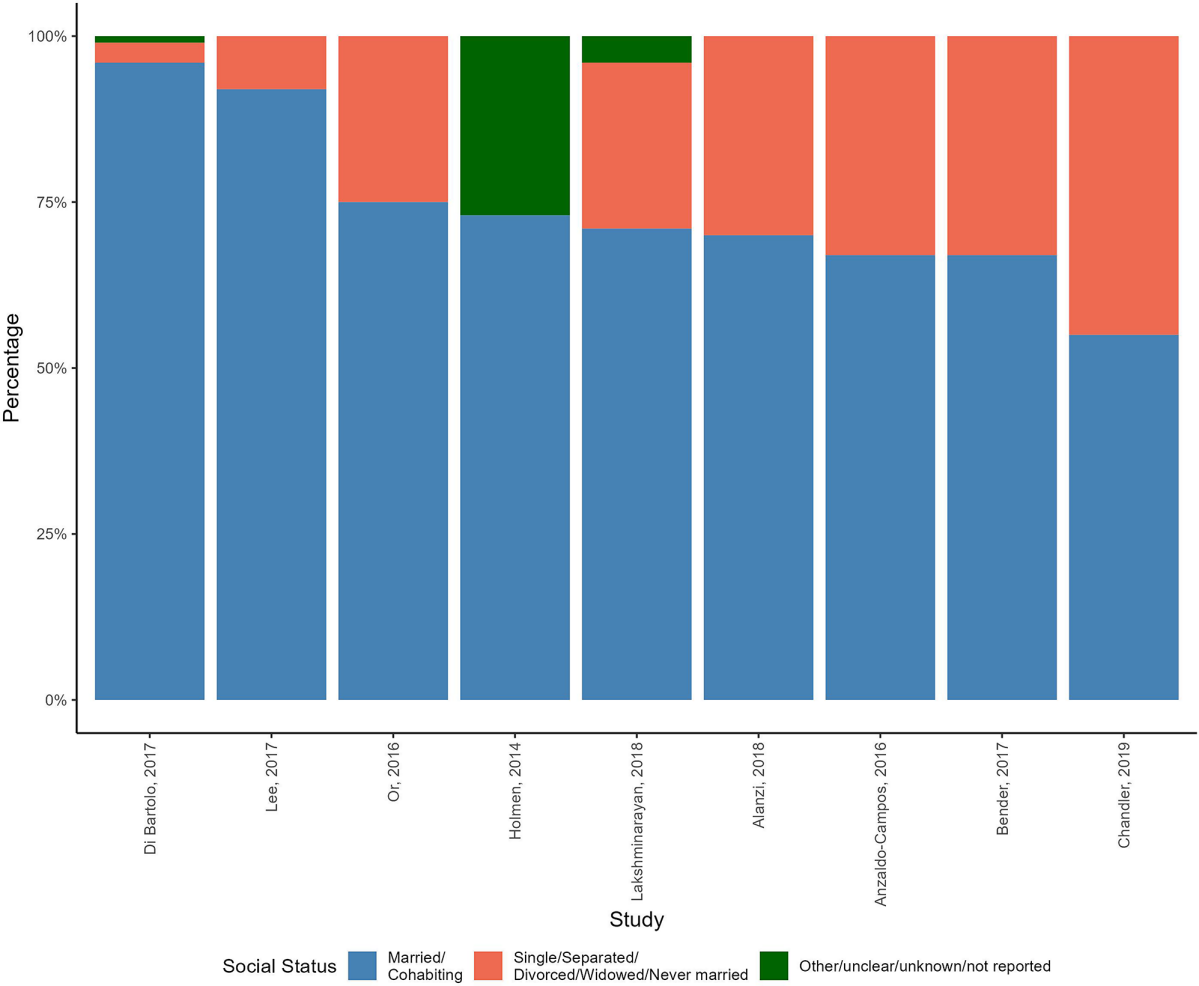
Social network/capital percentages of participants in the RCTs

All nine studies reporting on social capital had more than half of the participants married or living with someone (see Fig. 7 below and Table 5 in Additional file 11 for more details and descriptive analysis).

Seven studies reported on the income of their participants (see Table 6 in Additional file 11 for more details and descriptive analysis) [50, 56, 59, 69, 80, 83, 100]. One study additionally reported that 50% of participants had no access to a car [83]. Two studies mainly targeted participants of lower income or SEP [69, 83].

**Fig. 8:**
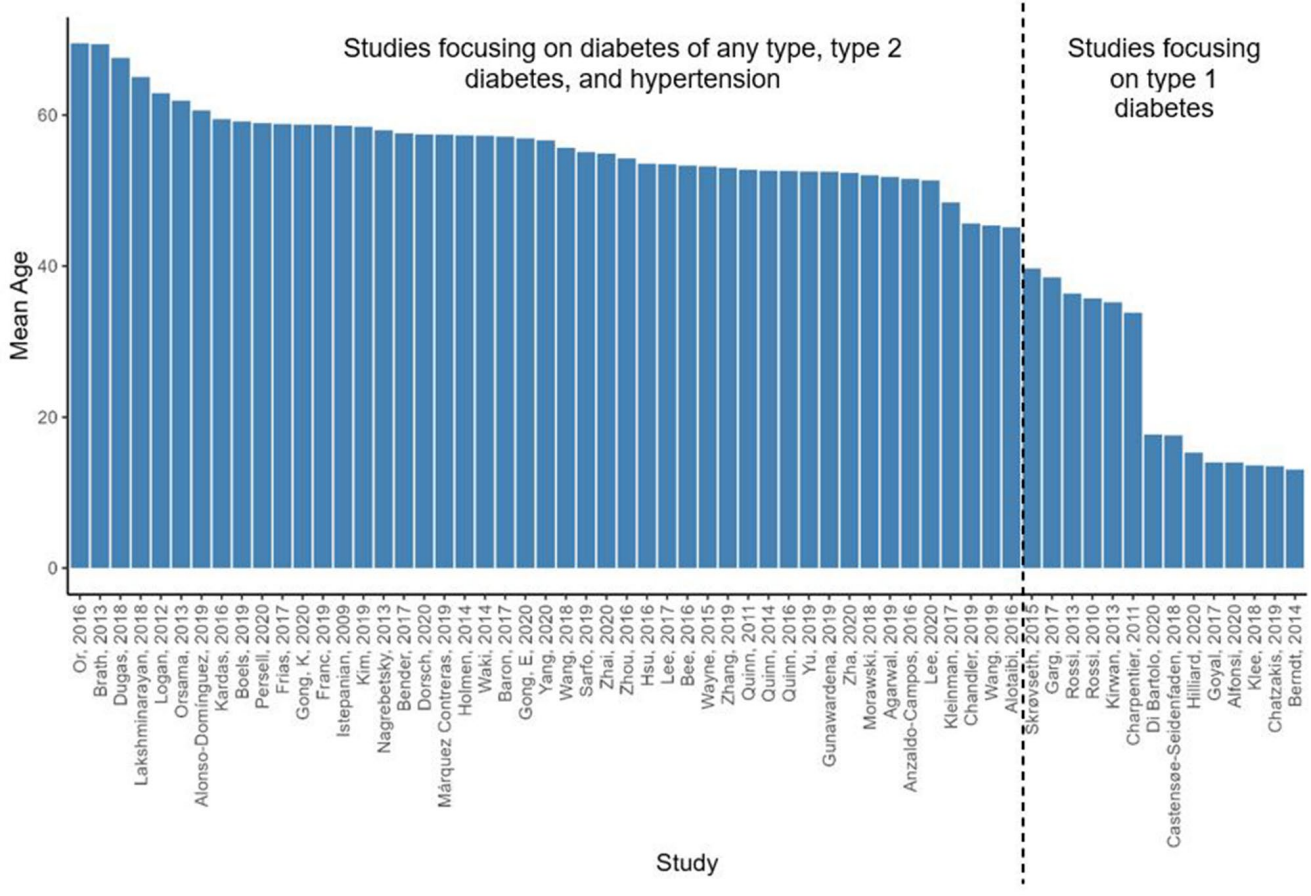
Mean age in the RCTs

In the 64 studies reporting on age, it ranged between 45 and 70 years in the studies of T2DM, diabetes of any type, hypertension, and both diseases (see Table 7 in Additional file 11 for more details and descriptive analysis). The participants in the studies focusing on T1DM were between 13 and 40 years (see Fig. 8).

Disability was reported twice. One study had 10.5% of the participants with a learning disability or mental health condition [118], and another had 31% categorized as disabled or unable to work [69].

Four studies reported their participants’ health insurance status [61, 66, 69, 83]. In a study from Canada, all participants were publicly insured [83]. The others took place in the US, with all participants privately insured [66], 22% insured by Medicare [69], and 26% publicly insured [61] in one study each.

### Reported subgroup analyses with PROGRESS-Plus

**Fig. 9:**
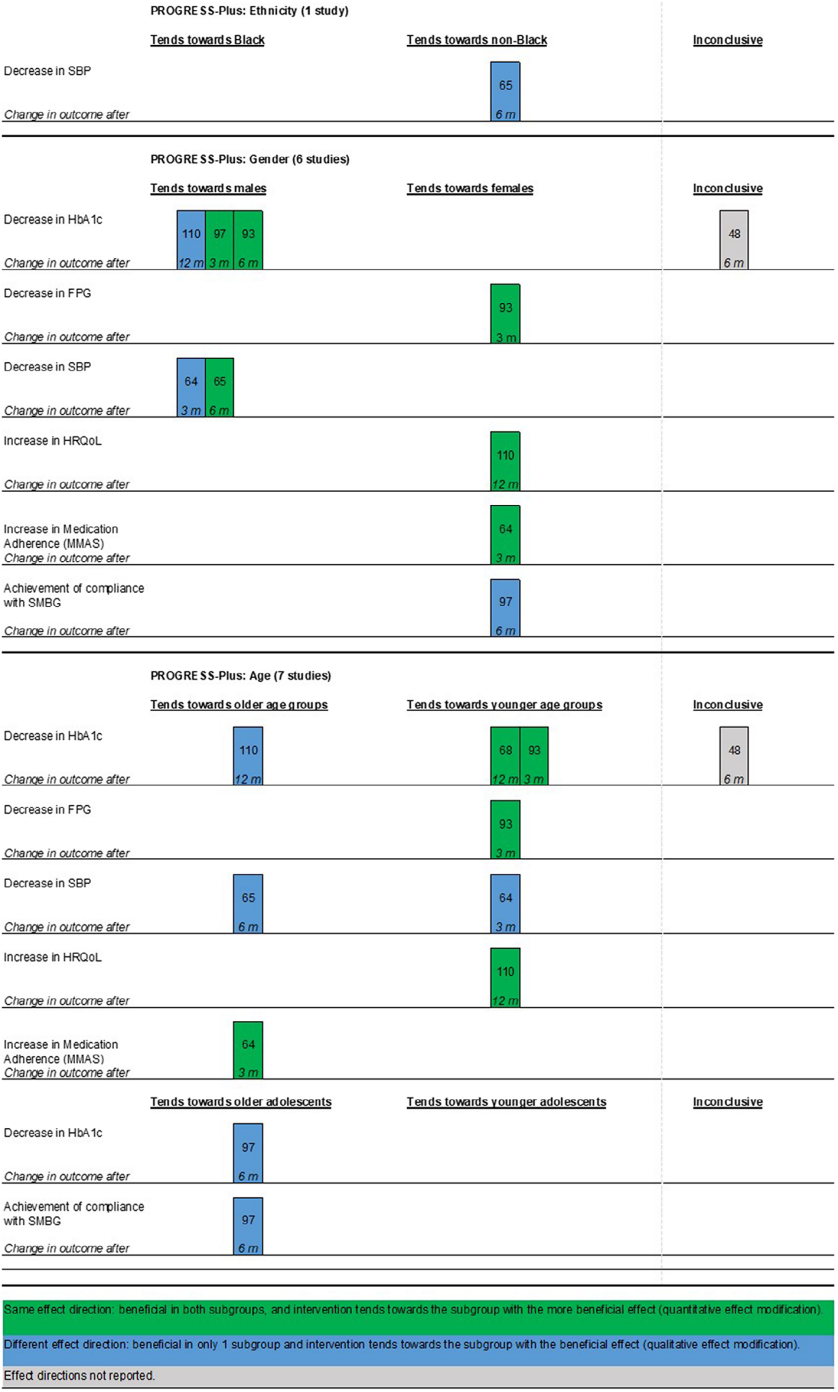
Harvest plot of the reported subgroup analyses. *Notes* The harvest plot illustrates the subgroup analyses. The position of the bar shows the tendency of the effect of mHealth towards a particular subgroup. The color of the bar shows the effect direction in the subgroups, whether both benefitted, both were harmed, or one was harmed and the other benefitted. The numbers inside the bars are the reference numbers of the studies. The italic numbers at the bottom of the bars are the months at which the outcome effect was measured. The harvest plot does not include the category “No difference between subgroups,” as illustrated in Additional file 5, because it was never met. Of note, harvest plots do not take into consideration the statistical significance of the effect estimates. Abbreviations FPG: fasting plasma glucose; HbA1c: glycated hemoglobin; HRQoL: health-related quality of life; m: months; SBP: systolic blood pressure

Seven studies reported subgroup analyses for mHealth app effectiveness with regard to at least one of three PROGRESS-Plus characteristics (ethnicity, gender, and age) [48, 64, 65, 68, 93, 97, 110]. Only for gender and age, respective subgroup analyses were available from more than one RCT, with effect estimates showing no general tendency toward a particular subgroup in either characteristic. Four subgroup analyses showed better health outcomes in females [64, 93, 97, 110] and five in males [64, 65, 93, 97, 110]. Five subgroup analyses showed better health outcomes in older individuals [64, 65, 97, 110] and five in younger individuals [64, 68, 93, 110]. There was a singular subgroup analysis for ethnicity; SBP improved in non-Black participants [65]. These results are visualized in the harvest plots in Fig. 9, and further details about concrete effect estimates, their statistical significance, and the type of subgroup analysis are in Table 1.

**Table 1:** Summary of the mHealth app effectiveness outcomes and subgroups analyses with PROGRESS-Plus (*N* = 7 studies)

Primary Author, Year (Disease)	Outcome Analyzed	PROGRESS-Plus Variable	Type of Subgroup Analysis^a^	Measured Effect Estimate	CI or *P*-Value of the Measured Effect	Effect Tendency^b^	Effect Direction^b^	Beneficial/Harmful^c^
Di Bartolo et al., 2017 [97] (T1DM)	HbA1c (%)	Age 14–17	Stratification	+ 0.06	*P* = 0.82	Effect tends towards older adolescents	Different effect direction	-
		Age 18–24	Stratification	-0.34	*P* = 0.21			+
		Men	Stratification	-0.16	*P* = 0.48	Effect tends towards men	Same effect direction	++
		Women	Stratification	-0.06	*P* = 0.83			+
	Achievement of compliance with SMBG (%)	Age 14–17	Stratification	-5.8	*P* = 0.57	Effect tends towards older adolescents	Different effect direction	-
		Age 18–24	Stratification	+ 4.3	*P* = 0.71			+
		Gender: man	Stratification	-4.9	*P* = 0.66	Effect tends towards women	Different effect direction	-
		Gender: woman	Stratification	+ 1.0	*P* = 0.93			+
Gong et al., 2020 [110] (T2DM)	HbA1c (%)	Gender: male	Stratification	-0.09	CI: (-0.65, 0.47) *P* = 0.75	Effect tends towards males	Different effect direction	+
		Gender: female	Stratification	+ 0.024	CI: (-0.57, 0.62) *P* = 0.94			-
		Age: <60	Stratification	+ 0.1	CI: (-0.54, 0.74) *P* = 0.75	Effect tends towards older age	Different effect direction	-
		Age: ≥60	Stratification	-0.21	CI: (-0.61, 0.18) *P* = 0.29			+
	HRQoL (score of AQoL-8D scale)	Gender: male	Stratification	+ 0.03	CI: (-0.02, 0.08) *P* = 0.30	Effect tends towards females	Same effect direction	+
		Gender: female	Stratification	+ 0.06	CI: (0.01, 0.11) *P* = 0.03			++
		Age: <60	Stratification	+ 0.05	CI: (0.00, 0.09) *P* = 0.05	Effect tends towards younger age	Same effect direction	++
		Age: ≥60	Stratification	+ 0.02	CI: (-0.03, 0.08) *P* = 0.44			+
Kleinman et al., 2017^d^ [48] (T2DM)	HbA1c (%)	Gender	Stratification	N/A	N/A	N/A	N/A	N/A
		Age	Stratification	N/A	N/A	N/A	N/A	N/A
Morawski et al., 2018 [64] (Hypertension)	Medication adherence (MMAS)	Gender: male	Stratification	+ 0.15	CI: (-0.29, 0.59)	Effect tends towards females	Same effect direction	+
		Gender: female	Stratification	+ 0.54	CI: (0.12, 0.96)			++
		Age: at or below median^e^	Stratification	+ 0.30	CI: (-0.14, 0.74)	Effect tends towards older age	Same effect direction	+
		Age: above median^e^	Stratification	+ 0.44	CI: (0.01, 0.88)			++
	SBP (mm Hg)	Gender: male	Stratification	-0.91	CI: (-6.29, 4.47)	Effect tends towards males	Different effect direction	+
		Gender: female	Stratification	+ 1.53	CI: (-2.46, 5.51)			-
		Age: at or below median^e^	Stratification	-1.32	CI: (-5.83, 3.20)	Effect tends towards younger age	Different effect direction	+
		Age: above median^e^	Stratification	+ 2.02	CI: (-2.60, 6.64)			-
Persell et al., 2020 [65] (Hypertension)	SBP (mm Hg)	Age: below median (< 61)	Stratification	+ 0.10	CI: (-3.8, 4.2)	Effect tends towards older age	Different effect direction	-
		Age: At or above median (≥ 61)	Stratification	-4.00	CI: (-8.1, 0.1)			+
		Gender: male	Stratification	-3.60	CI: (-8.1, 0.9)	Effect tends towards males	Same effect direction	++
		Gender: female	Stratification	-0.80	CI: (-4.5, 2.9)			+
		Race: non-Black	Stratification	-3.70	CI: (-7.3, − 0.1)	Effect tends towards non-Black	Different effect direction	+
		Race: Black	Stratification	+ 1.20	CI: (-3.8, 6.1)			-
Quinn et al., 2016^f^ [66, 68] (T2DM)	HbA1c (%)	Age: <55	Interaction + stratification	-1.0	CI: (-1.8, -0.2) *P* = 0.02	Effect tends towards older age	Same effect direction	+
		Age: ≥55	Interaction + stratification	-1.4	CI: (-2.3, -0.6) *P* = 0.001			++
Yang et al., 2020 [93] (T2DM)	HbA1c (%)	Age: <60	Interaction	-0.44	CI: (-0.74, -0.15)	Effect tends towards younger age	Same effect direction	++
		Age: ≥60	Interaction	-0.14	CI: (-0.40, 0.13)			+
		Gender: male	Interaction	-0.35	CI: (-0.66, -0.03)	Effect tends towards males	Same effect direction	++
		Gender: female	Interaction	-0.23	CI: (-0.47, 0.01)			+
	FPG (mg/dL)	Age: <60	Interaction	-22.50	CI: (-40.62, -4.38)	Effect tends towards younger age	Same effect direction	++
		Age: ≥60	Interaction	-11.17	CI: (-27.00, 4.67)			+
		Gender: male	Interaction	-14.46	CI: (-33.27, 4.35)	Effect tends towards females	Same effect direction	+
		Gender: female	Interaction	-19.25	CI: (-34.20, -4.30)			++

*Notes* The table shows the differential effectiveness of mHealth app by several characteristics from PROGRESS-Plus. Taking the HbA1c results among the age subgroups of Di Bartolo et al., 2019 as an example, the interpretation would be: HbA1c increased in age group 14–17 with the use of mHealth app by 0.06% more than the comparison group, and HbA1c decreased in age group 18–24 with the use of mHealth app by 0.34% in comparison to the control group. The point estimate therefore suggested a detrimental effect of mHealth app use in the 14–17 year age group. Note that all interpretations on “harm” or “benefit” are based on point estimates only, and do not take statistical significance into account. These may well be chance results and are compatible with no substantial effect modification

^a^ In *Type of Subgroup Analyses*, interaction subgroup analysis refers to having an interaction term between the intervention and the subgroup, and stratification subgroup analyses refers to analyzing separately the intervention effect within the subgroups. When only an interaction analysis occurred, the effect estimate represented the difference in the intervention effect between the subgroups, i.e., the effect in one subgroup relevant to the other. When only a stratification occurred, the effect estimate was the intervention effect in a particular subgroup. By "intervention effect", we refer to the mean differences between intervention and the control groups

^b^*Effect Tendency* and *Effect Direction* do not consider the statistical significance of the effect estimates, but rather the quantitative effect modification in cases when the effect was either better in all subgroup or worse in all subgroups, and the qualitative effect modification when in one subgroup the effect was better and in the other subgroup it was worse

^c^ The column *Beneficial/Harmful* represents a visual summary of the columns *Effect Tendency* and *Effect Direction*. The “+” or “-“ under Beneficial/Harmful represent the improvement (+) or worsening (-) of the effect in the subgroups with different effect direction, and “++” refers to better effect in comparison to the other subgroup where both subgroups benefitted/harmed from the intervention

Abbreviations: CI: confidence interval; FPG: fasting plasma glucose; HbA1c: glycated hemoglobin; HRQoL: health-related quality of life; MMAS: Morisky medication adherence scale; N/A: not applicable; SBP: systolic blood pressure; SMBG: self-monitoring of blood glucose; T1DM: type 1 diabetes mellitus; T2DM: type 2 diabetes mellitus

^d^ The study only stated that the subgroup analysis did not have statistically significant results without providing the results, making it hard to state the effect direction and the tendency towards a certain subgroup without the statistical significance

^e^ Median age not specified in the study

^f^ The study (Quinn et al., 2016) had a typo in the confidence interval reported in the article, but the typo is corrected in the table above

## Discussion

### Summary of findings

Our systematic assessment of 65 effectiveness trials investigating mHealth app effectiveness among diabetic and hypertensive patients showed that descriptions of the study population regarding inequality-relevant characteristics are currently heterogeneous and often incomplete. Even though, judging from the reported descriptives, most studies did collect information on at least some relevant inequality factors in their recruited patient samples, they rarely used this information to investigate differential effectiveness. Age and gender were by far most frequently reported, with mostly expected distributions, followed by education, ethnicity, and occupation. In contrast, descriptives for social capital, SEP, insurance status, place of residence, religion, and disability were rare, and for sexual orientation were never reported. This lack of appropriate descriptions of the underlying study population makes it difficult to assess to which extent those RCTs adequately represent potentially vulnerable population subgroups. Furthermore, the countries with a higher burden of both diseases are low- and middle-income countries [120, 121], yet most of the RCTs we identified took place in high-income countries and none in low-income countries. This leaves evidence gaps for some of the most vulnerable populations [122, 123]. Only seven of the 65 studies reported inequality-related subgroup analyses for mHealth app effectiveness, mostly by gender and age, with a singular analysis by ethnicity.

General user profiles for mHeath apps were previously reported to be younger, better educated, and with higher income [124]. These profiles somehow match the frequencies of our three most reported characteristics of the participants which were age, gender, and education, and the most analyzed in subgroup analyses which was age. This shows the interest in understanding the generalizability and heterogeneity of the results among the presumably most relevant moderators.

It is noteworthy that the place of residence, ethnicity, social networks/capital, income, and insurance status were insufficiently or rarely evaluated as potential effect moderators. This may in part be because these subpopulations have lower access to mHealth care and, therefore, may have been underrepresented in our pool of studies which included only populations with access to mHealth. Specifically, because mHealth apps are heralded as bridging geographical, social, and financial barriers, it should be in the interest of app developers, policy makers, health service providers, and patients to investigate if these aspirations are fulfilled. Similarly, disability should be considered a vital stratification factor, as mHealth promises to better cater and tailor services for people living with disabilities [17, 125]. However, disability was considered in only two studies. For religion and sexual orientation, on the other hand, which are relevant to many health inequalities, it seems more understandable that they were rarely investigated as there is little evidence to support the assumption that they specifically moderate mHealth effectiveness in diabetes and hypertension.

Our study primarily highlights the significant gap in considering relevant socioeconomic and sociocultural inequality characteristics among study populations, and a gap in analyzing these characteristics in subgroup analyses. Similar to other reviews assessing inequality/inequity factors using PROGRESS-Plus in diabetes care [34, 126, 127] and other health indications [28, 33, 35], inequality characteristics are overall currently not sufficiently considered in RCTs on mHealth app effectiveness. The frequencies of the characteristics match those reported in previous reviews [33, 34, 126, 127] except for place of residence, ethnicity and health insurance which were reported more in some reviews about diabetes care [34, 126, 127]. Our findings also match the findings of previous studies that show limited consideration of inequality characteristics through subgroup analyses [35], with gender and age being the most frequently analyzed [28, 33]. Although the subgroup analyses showed inconsistent and weak tendencies towards particular subgroups, adding an equity lens on the implications of these tendencies with respect to the disease burden/dominance among the subgroups is crucial in identifying health inequity concerns.

In gender and age subgroup analyses, mHealth app effectiveness did not tend towards particular subgroups. Regarding gender, SBP in hypertension and HbA1c in T1DM and T2DM improved in men with the use of mHealth [64, 65, 93, 97, 110], potentially alleviating their relatively higher disease burden in these conditions [19, 128–131]. On the one hand, more behavioral outcomes such as HRQoL, medication adherence, and compliance to self-monitoring of blood glucose were reported to improve with the use of mHealth among females than males. On the other hand, clinical outcomes such as HbA1c, FBG, and SBP seemed to improve with the use of mHealth among males rather than females. Worth noting that such heterogeneity in health outcomes, while justified and necessary to assess the extent of effectiveness of mHealth apps, challenges grouping the outcomes to compare and provide stronger evidence of differential effectiveness from subgroup analyses. Regarding age, diabetes control was slightly better among younger individuals using mHealth [68, 93, 110], which hints at potential inequity as the disease worsens with age [132, 133], and first and second digital divides (i.e. inequalities in access and use) already exist between older and younger individuals [17, 26].

Regarding other characteristics, a singular subgroup analysis [65] raised some inequity concerns as SBP increased with using an mHealth app in individuals of Black racial/ethnic backgrounds, who already suffer a higher disease burden of hypertension [134]. Surprisingly, no underserved populations under SEP, in terms of income, education, or employment, were included in the subgroup analyses.

Several studies had a RoB in the measurement of the outcomes included in subgroup analyses due to the nature of the intervention, as participants were not blinded to their allocation. The potential for selection bias could not be completely ruled out in parallel and cluster RCTs where recruitment of participants occurred after randomization.

As RCTs are not usually powered for subgroup analyses, no single RCT can provide definite evidence on differential effectiveness among individuals with different sociocultural and socioeconomic characteristics. However, such evidence on differential effectiveness of mHealth apps could be generated through meta-analyses of subgroup results, collected through systematic reviews or assessments such as ours. A necessary prerequisite is a sufficient number of RCTs conducts and reports stratified and subgroup analyses for those inequality characteristics. To date, this is not the case.

We cannot therefore provide a definitive answer to the question if inequality factors moderate the effectiveness of mHealth apps among diabetes and hypertensive patients. Moreover, we found that the potential for quantitative synthesis is currently hampered by the heterogeneity of outcome measures and PROGRESS Plus subgroup operationalizations. In practice, these knowledge gaps may continue to limit the evidence base for decision makers and healthcare providers to assess and mitigate the risk of increasing health inequalities in the face of ongoing digitalization of healthcare systems. This complements existing challenges hindering the expansion of mHealth public health interventions [135]. Ideally, evidence on all three digital divides (i.e. inequalities in access, use, and differential effectiveness) could jointly inform policy interventions aiming to ease access to and usage of mHealth apps and mitigating consequences of differential effectiveness. These interventions would result in better inclusion and benefit from mHealth usage across disadvantaged groups [136] and could include providing senior citizens with digital training, outreach to rural areas, Wi-Fi hotspots, or financial reimbursement [137].

### Strengths and limitations

A main strength of this study is that we comprehensively assessed the consideration of inequality factors, particularly in RCTs of mHealth apps among specifically diabetic and hypertensive patients, while evaluating how these factors moderate the app’s effectiveness. Previous studies had different disease indications [28, 33, 138] or different interventions and study designs [34, 126, 127]. We also had a specific scope of choosing only studies with an app-based intervention and no other mHealth interventions such as text messages. Limiting the study design to RCTs was carried over from the umbrella review [29] and strengthened our evidence base, as RCTs are considered the gold standard for effectiveness research [139].

Our systematic assessment comes with limitations related to our conduct of the assessment and to the weaknesses of the included studies. Although limiting the study design to RCTs strengthens internal validity, it limits the external validity of the results. Moreover, RCTs are not usually meant for detecting inequalities; randomization usually ensures balanced characteristics of included patients across study arms, but RCTs might completely exclude or oversample certain populations. In our included RCTs all patients had access or were provided access to mHealth apps thereby limiting any insight into inequalities in access to and usage of mHealth apps (first and second digital divides, respectively) despite their undisputed relevance in the digital age. Thus, it should be noted that, per design, our study can only provide insights into inequalities in benefit of usage (third digital divide).

In the light of our unconventional study design, we refrain from calling our study a SR despite being aligned with the methodology of a SR in acknowledgement of our lack of a search strategy which is an integral part of a traditional systematic review. We aimed to increase the efficiency of the evidence generation process, as many systematic reviews on mHealth app effectiveness in diabetic and/or hypertensive patients have already been conducted with overlapping PICO criteria as our study. Implementing a separate search strategy to re-identify either the underlying RCTs or the existing systematic reviews of these RCTs from scratch would have, in our opinion, duplicated previous efforts without adding substantially to the evidence base. We believe our pool of studies was in the end comprehensive and covered most relevant studies on mHealth apps in diabetes and/or hypertensive patients. Nevertheless, we acknowledge that not conducting a search strategy might have slightly decreased the recency of included studies.

To keep our population coherent as the main users of the mHealth app in the intervention, we excluded a big cluster RCT in India with over 4000 participants because the app was used by the healthcare staff and not the patients [140]. The study also conducted several subgroup analyses on the improvement of HbA1c and SBP with place of residence (town type and population size), gender, education, and age. We moreover might have missed relevant studies since we based our studies only on the umbrella review SRs. For example, we excluded a borderline article that included several socio-economic characteristics of their participants (ethnicity, education, gender, social capital, income, age, and insurance) as it was a secondary analysis of an RCT [141], and the original RCT was not in our pool of identified studies. However, as the RCT population was not exclusively hypertensive and/or diabetic patients, in this particular case, the primary RCT would have been excluded even if it had been part of our pool of potentially eligible studies [142].

Regarding the included studies, several inconsistencies in reporting education, ethnicity, income, and other descriptives made analyses and general conclusions about the populations represented challenging. The inconsistency in the reporting of the educational status is especially limiting as education is a significant modifier pertaining to both SEP and the health literacy of the participants [17]. There was some selection bias in the studies that reported socioeconomic/sociocultural characteristics of the participants, as some specifically targeted underserved populations. Moreover, as participants could not be blinded to their intervention given its nature, this may have affected the internal validity of the studies. Unfortunately, no studies regarding gestational diabetes were identified, which leaves a gap in supporting pregnant women who might face different physical and psychological challenges from other diabetic groups, especially if they belong to a minority population and face discrimination [143–145].

### Implications for future research

It would be useful to replicate our methodology and include an updated pool of RCTs on mHealth effectiveness while potentially focusing on the subgroup analyses of mHealth app effectiveness for PROGRESS-Plus. Conducting a meta-analysis could also be beneficial in deriving (in)equality conclusions. We call for more frequent consideration of subgroup analyses for inequality characteristics when conducting RCTs, as merely considering them descriptively is insufficient to derive equity/inequity conclusions [35]. We also join previous research in encouraging more multi-dimensional analyses combining several inequality/inequity factors in subgroup analyses to tackle intersectionality [35, 146].

Investigating inequality aspects of mHealth apps in other chronic diseases that could be self-managed, such as obesity or mental health diseases, is recommended, to assess their potential to lower the burden on the healthcare system [147]. We encourage adding more SDOH, such as health literacy [18, 19], which is an underlying factor challenging minority/underprivileged populations from properly using and benefitting from mHealth even when provided access [17, 24, 148]. Including other study types would also be interesting, taking into account real-world evidence with higher external validity [149]. Further research could additionally go beyond differential effectiveness of mHealth and explore the socioeconomic and sociocultural characteristics of users of apps in routine care (e.g. users of DiGa in Germany, mHealthBelgium in Belgium [150], or ApiApps in France [151]).

## Conclusions

Our study reported on the considerations of inequalities and their moderation of the effectiveness of mHealth apps in 65 RCTs and 72 published articles. Our findings show a significant gap in the consideration of inequality factors whether as mere descriptives of the recruited populations or in subgroup analyses. If at all, the studies reported mainly descriptively on inequality factors but barely investigated differential effectiveness. We therefore were not able to conclude if inequality factors moderate mHealth app effectiveness among subgroups of diabetes and hypertensive patients. Our results showed that the age, gender, and education of participants were most frequently considered as descriptives of study populations, while information on sociocultural and socioeconomic characteristics was either lacking or insufficiently reported and analyzed. Subgroup analyses were few and sparse, not allowing for proper analysis of health inequalities mHealth app effectiveness. For future research, we encourage building on our study and analyzing more SDOH with respect to the effectiveness of mHealth apps both in diabetes and hypertension, and in other chronic health conditions.

## Electronic supplementary material

Below is the link to the electronic supplementary material.

Supplementary Material 1

Supplementary Material 2

Supplementary Material 3

Supplementary Material 4

Supplementary Material 5

Supplementary Material 6

Supplementary Material 7

Supplementary Material 8

Supplementary Material 9

Supplementary Material 10

Supplementary Material 11

## Data Availability

All data generated or analysed during this study are included in this manuscript [and its additional files].

## Abbreviations

App: Application
CENTRAL: Central Register of Controlled Trials
CI: Confidence interval
CINAHL: Cumulative Index to Nursing and Allied Health Literature
CNKI: China NAtional Knowledge Infrastructure
DALY: Disability-adjusted life year
DiGa: Digital health applications
FPG: Fasting plasma glucose
HbA1c: Glycated hemoglobin
HRQoL: Health-related quality of life
HTN: Hypertension
ITT: Intention-to-treat
LILACS: Latin American and Caribbean Health Sciences Literature
mHealth: Mobile health
MMAS: Morisky medication adherence scale
N/A: Not applicable
OSF: Open Science Framework
PICOS: Population, intervention, comparison, outcome, study design
PRISMA: The Preferred Reporting Items for Systematic reviews and Meta-Analyses
RCT: Randomized controlled trials
RoB: Risk of Bias
SBP: Systolic blood pressure
SDOH: Social determinants of health
SEP: Socioeconomic position
SES: Socioeconomic status
SMBG: Self-measurement of blood glucose
SR: Systematic review
SWiM: Synthesis without a meta-analysis
T1DM: Type 1 diabetes mellitus
T2DM: Type 2 diabetes mellitus
US: United States
WHO: World Health Organization
