# Supplementary material for: Consideration of inequalities in effectiveness trials of mHealth applications – a systematic assessment of studies from an umbrella review
Source: Int J Equity Health. 2024 Sep 11;23:181. doi: 10.1186/s12939-024-02267-4 (PMC11389088; doi:10.1186/s12939-024-02267-4)
Supplement: Supplementary file 2 — Supplementary Material 2 [file 12939_2024_2267_MOESM2_ESM.docx]

Additional File 2. Eligibility Criteria (PICOS Framework)

*Table 1: Eligibility Criteria based on PICOS Framework*

| PICOS Item | Description within study context |
| --- | --- |
| Population | Patients diagnosed with diabetes (type 1 diabetes mellitus [T1DM], type 2 diabetes mellitus (T2DM), or gestational diabetes), hypertension, or both. If the population of the study suffered comorbidities with diabetes and/or hypertension, the study was still included. Prediabetes was not considered. |
| Intervention | An intervention of mHealth app: a program/software installed/downloaded/delivered through a smartphone or a tablet device; the app received input from the user and provided feedback or other functions [[1-3](#_ENREF_1)]. The app could be a standalone intervention or complementary to other intervention tools such as text messaging, wearable devices, etc.  The intervention was excluded if it was not specifically a digital *health* app but rather solely a text messaging app, a wearable device, a videocall app, or a web-delivered service. |
| Comparison | Any comparison group including no intervention, standard/usual care, and additional or alternative interventions. |
| Outcome | The main outcome of interest was direct or indirect changes in health-related outcomes, disease management, or improvement in the health status of the population in the study. This was indicated through, but not excluded to changes in clinical outcomes such as HbA1c levels, SBP and DBP, or improvement in symptoms. Also, we included changes in non-clinical or behavioral outcomes such as medication adherence. We further included outcomes that affected patients’ health indirectly, such as physician prescriptions. Changes in health outcomes were considered irrespective of being primary or secondary outcomes in the primary studies. |
| Study Design | The studies were limited to effectiveness and efficacy randomized control trials (RCTs). If the study was labelled differently (e.g., as a clinical control trial [CCT]), but the methods clearly described a process for randomized allocation then it was included. Only studies published in English were considered following the restriction in the umbrella review and assuming that the risk of losing studies that are not written in English in the domain of digital health would be relatively low. |

**References**

1. Cai X, Qiu S, Luo D, Wang L, Lu Y, Li M. Mobile Application Interventions and Weight Loss in Type 2 Diabetes: A Meta-Analysis. Obesity (Silver Spring). 2020;28(3):502–9.

2. Hanna KT, Wigmore I. What is a mobile app (mobile application)? – TechTarget Definition: Tech Target; [updated 20-Feb-23. Available from: <https://www.techtarget.com/whatis/definition/mobile-app>.

3. Peng Y, Wang H, Fang Q, Xie L, Shu L, Sun W, Liu Q. Effectiveness of Mobile Applications on Medication Adherence in Adults with Chronic Diseases: A Systematic Review and Meta-Analysis. J Manag Care Spec Pharm. 2020;26(4):550–61.
