# Supplementary material for: Consideration of inequalities in effectiveness trials of mHealth applications – a systematic assessment of studies from an umbrella review
Source: Int J Equity Health. 2024 Sep 11;23:181. doi: 10.1186/s12939-024-02267-4 (PMC11389088; doi:10.1186/s12939-024-02267-4)
Supplement: Supplementary file 3 — Supplementary Material 3 [file 12939_2024_2267_MOESM3_ESM.docx]

Additional File 3. Information on Risk of Bias 2 Tool

**Further information on RoB-2 tool**

The RoB-2 tool comprises signaling questions for five domains: randomization process, deviations from intended interventions, missing outcome data, measurement of the outcome, and selection of the reported result. An algorithm combines the assessments for each domain to generate a final judgment of low, some concerns, or high risk of bias.

Four reviewers (AJS, JB, LS, and NA) piloted assessing three outcomes independently and compared their decisions to apply the decision rules to the rest of the RoB process

**Additional decision rules:**

Only three questions did not have a clear advised cutoff: 1.3 in the randomization domain 2.3 in the deviations from intended outcomes domain, and 4.5 in the measurement of the outcome domain. For 1.3, we chose a cutoff of 10% difference between at least two baseline characteristics in the intervention groups to allow for differences due to chance. For 2.3 we assumed that it would be unlikely that there were deviations from the protocol due to the trial context. For 4.5, when the outcome assessors were not the participants, we decided that there is a likelihood that the assessment of the outcome was influenced by the knowledge of the intervention when the authors of the study were the creators of the app being used in the intervention.
