# Supplementary material for: Consideration of inequalities in effectiveness trials of mHealth applications – a systematic assessment of studies from an umbrella review
Source: Int J Equity Health. 2024 Sep 11;23:181. doi: 10.1186/s12939-024-02267-4 (PMC11389088; doi:10.1186/s12939-024-02267-4)
Supplement: Supplementary file 4 — Supplementary Material 4 [file 12939_2024_2267_MOESM4_ESM.docx]

Additional File 4. PROGERSS-Plus Framework

| PROGRESS-Plus characteristics by Oliver et al. [1] | Description of possible variable levels by Oliver et al. [1] | Description/ Adaptation for the current study | Further harmonization/ aggregation of reported variable levels for our analysis |
| --- | --- | --- | --- |
| Place of residence | Rural/urban, country/state, housing characteristics | Same as Oliver et al. | Different variable categorizations were used across papers, which we present in their original form without further harmonization/aggregation. |
| Ethnicity | Ethnic background | We found inconsistent use of the terms race and ethnicity across studies, so we considered and extracted both. | We formed the following groups:   - Minority: minorities depending on the dominant ethnic group of a country (e.g. non-White participants in the studies that took place in countries where White is the main race/ethnicity). - Majority: majorities depending on the dominant ethnic group of a country (e.g. White participants in the studies that took place in countries where White is the main race/ethnicity). - Others (not graphically represented): percentages of participants refusing to answer or missing |
| Occupation | Professional, skilled, unskilled, unemployed etc. | Same as Oliver et al. | We formed the following groups:   - Employed: any type of full- or part-time employment. - Unemployed: as stated by the study (e.g. unemployed, not working). - Retired/disabled: as stated by the study. - Other: homemakers, students, not disclosed, and others as reported by the study. - Unclear/not reported: the percentage difference to 100% after adding up the percentages of all explicitly reported and labelled occupational statuses. |
| Gender | Male/men or female/women | Same as Oliver et al. | The same variable categorization was used consistently across papers with no need for further harmonization/aggregation. |
| Religion | Religious background | Same as Oliver et al. | The same variable categorization was used consistently across papers with no need for further harmonization/aggregation. |
| Education | Years in and/or level of education attained, school type | Same as Oliver et al. | We formed the following groups following the International Standard Classification of Education (ISCED) [2]:   - Low education: below secondary - Intermediate education: between secondary and less than tertiary/university education - High education: tertiary/university and above. - Other: refuse to answer, illiterate, unknown, no education, etc. |
| Social Capital | Neighborhood/community/ family support | Same as Oliver et al. | We formed the following groups:   - Married/cohabiting: people not living alone - Single/separated/divorced/widowed/never married: people living alone. - Other/unclear/unknown/not reported: either stated by the study or the percentage difference to 100% after adding up the percentages of all explicitly reported and labelled social capital statuses. |
| SEP | Income, means tested benefits/welfare, affluence measures, etc. | Same as Oliver et al. | Different variable categorizations were used across papers, which we present in their original form without further harmonization/aggregation. |
| All SEP | SEP income related, plus occupation, education, and elements of place of residence | Same as Oliver et al. +  insurance status | The same variable categorization was used consistently across papers with no need for further harmonization/aggregation. |
| Age | Age range | Same as Oliver et al. / mean age | We calculated the weighted mean age in each study and used it in our representations and analysis. |
| Disability | Existence of physical or emotional/mental disability | Same as Oliver et al. | The same variable categorization was used consistently across papers with no need for further harmonization/aggregation. |
| Sexual orientation | Heterosexual, gay, lesbian, bisexual, transgender | Same as Oliver et al. | Not applicable |
| Other vulnerable groups | School non-attenders, looked after young people, young people in criminal justice system, victims of abuse, runaways, teenage parents | Not applicable | Not application |

**Notes**: Adapted from Oliver et al. [1]. The table includes the description and adaptation of the criteria in the PROGRESS-Plus framework.

In the protocol, the reference cited was Kavanagh et al. [3] as the source from which the table was adapted since it was published before Oliver et al.. However, the more commonly cited and the more elaborate report of PROGRESS-Plus is Oliver et al. therefore the reference was changed from the protocol. Oliver et al. is also cited by Cochrane [4].

Abbreviations: SEP: socioeconomic position.

**References**

1. Oliver S, Kavanagh J, Caird J, Lorenc T, Oliver K, Harden A, et al. Health promotion, inequalities and young people's health. 2008.

2. eurostat. International Standard Classification of Education (ISCED): eurostat; [updated 06-Jan-23. Available from: <https://ec.europa.eu/eurostat/statistics-explained/index.php?title=International_Standard_Classification_of_Education_(ISCED)#Implementation_of_ISCED_2011_.28levels_of_education.29>.

3. Kavanagh J, Oliver S, Lorenc T. Reflections on developing and using PROGRESS-Plus. Equity Update. 2008;2(1):1-3.

4. Cochrane. PROGRESS-Plus | Cochrane Equity [updated 08-Nov-22. Available from: <https://methods.cochrane.org/equity/projects/evidence-equity/progress-plus>.
