# Supplementary material for: Consideration of inequalities in effectiveness trials of mHealth applications – a systematic assessment of studies from an umbrella review
Source: Int J Equity Health. 2024 Sep 11;23:181. doi: 10.1186/s12939-024-02267-4 (PMC11389088; doi:10.1186/s12939-024-02267-4)
Supplement: Supplementary file 5 — Supplementary Material 5 [file 12939_2024_2267_MOESM5_ESM.docx]

Additional File 5. Illustration and explanation of the harvest plot design

**

*Figure 1: Illustration and explanation of the harvest plot design*

**Notes**: The harvest plot comprises a section for each PROGRESS-Plus characteristic (e.g. gender). In each section, health outcomes (e.g. HbA1c) and the months after which the outcome changed are displayed. The bars represent the studies which report on the respective outcome in the respective subgroups. The columns represent the subgroup categories within the characteristic (e.g. men/women) and the vertical position of the bar under one of these columns represents the tendency of the effect towards this subgroup. In the pre-specified study protocol, we planned to call the effect directions as “no effect”, “increasing inequalities” and “decreasing inequalities”, but changed the originally intended wording to avoid attempting to make definitive equality claims.

*Box 1: Explanation of the harvest plot elements*

**There are four main categories of effect tendencies represented by the columns:**

- 1. Tendency towards subgroup X: for a particular outcome, the effect reported was higher/more beneficial/less harmful in subgroup X than subgroup Y.
  2. No difference between subgroups, explained by a true null effect:
     - no interaction between subgroup and intervention (0.00),
     - stratified analysis with exact similar effect size in both subgroups,
     - reported indifference in effects between the subgroups.
  3. Tendency towards subgroup Y: for a particular outcome, the effect reported was higher/more beneficial/less harmful in subgroup Y than subgroup X.
  4. Inconclusive effect:
     - whenever a PROGRESS-Plus criterion (e.g. education) was represented by more than two subgroups (e.g. low, intermediate, high) with varying effect estimates resulting in neither a consistent increase nor decrease in the effect estimates across the ordered subgroup categories this was interpreted as an inconclusive effect tendency, favoring neither clearly one or the other end of the subgroup category spectrum.
     - reported indifference in effect between the subgroups based on statistical insignificance (and not because of a true null effect).

**The color of the bar shows the effect direction between the subgroups:**

1. Same beneficial effect direction (green color): when both subgroups for example had a decrease in HbA1c and therefore both benefited (quantitative effect modification).
2. Different effect direction (blue color): when for example one subgroup had an increase and the other a decrease in HbA1c, and therefore one of the subgroups benefitted and the other was harmed (qualitative effect modification).
3. Not reported (grey color): when the study did not report the effect estimate.
4. Same harmful effect direction (red color): when both subgroups for example had an increase in HbA1c and therefore both were harmed.
