# Supplementary material for: Consideration of inequalities in effectiveness trials of mHealth applications – a systematic assessment of studies from an umbrella review
Source: Int J Equity Health. 2024 Sep 11;23:181. doi: 10.1186/s12939-024-02267-4 (PMC11389088; doi:10.1186/s12939-024-02267-4)
Supplement: Supplementary file 6 — Supplementary Material 6 [file 12939_2024_2267_MOESM6_ESM.docx]

Additional file 6. Screening and Selection

**List of 24 systematic reviews from the umbrella review that included RCTs on diabetes and/or hypertension**

| **Systematic Review Title** | **Systematic Review Author** | **Search ended** | **Number of Databases** | **Databases** |
| --- | --- | --- | --- | --- |
| Mobile Apps to Improve Medication Adherence in Cardiovascular Disease: Systematic Review and Meta-analysis | Al-Arkee et al., 2021 [[1](#_ENREF_1)] | Jan 20 | n= 7 | MEDLINE [Ovid], PubMed Central, Cochrane Library, CINAHL Plus, PsycINFO [Ovid], Embase [Ovid], and Google Scholar |
| Effectiveness of Mobile App-Based Interventions to Support Diabetes Self-Management: A Systematic Review | Amalindah et al., 2020 [[2](#_ENREF_2)] | Jan 20 | n=4 | Scopus, Medline, CINAHL, and Proquest |
| Do mobile device apps designed to support medication adherence demonstrate efficacy? An systematic review of randomised controlled trials, with meta-analysis | Armitage et al., 2020 [[3](#_ENREF_3)] | Nov 18 | n= 5 | Medline/PubMed, PsycINFO, Cumulative Index to Nursing and Allied Health Literature, Embase, and Web of Science |
| Efficacy of Mobile Apps to Support the Care of Patients with Diabetes Mellitus: a Systematic Review and Meta-Analysis of Rnadomized Controlled Trials | Bonoto et al., 2017 [[4](#_ENREF_4)] | 2016 | n= 3 | MEDLINE (PubMed), Cochrane Register of Controlled Trials (CENTRAL), and LILACS (Latin American and Caribbean Health Sciences Literature) |
| Mobile Application Interventions and Weight Loss in Type 2 Diabetes: A Meta-Analysis | Cai et al., 2020 [[5](#_ENREF_5)] | May 2019 | n=2 | PubMed and Scopus |
| T2DM Self-Management via Smartphone Applications: A systematic Review and Meta-Analysis | Cui et al., 2016 [[6](#_ENREF_6)] | Jun 16 | n=3 | PubMed, the Cochrane Library, and EMBASE |
| On the efficacy of behavior change techniques in mHealth fo self-management of diabetes: A meta-analysis | El-Gayar et al., 2021 [[7](#_ENREF_7)] | Oct 2020 | n=3 | PubMed, Medline and Web of Science |
| Impact of DSMES app interventions on medication adherence in type 2 diabetes mellitus: a systematic review and meta-analysis | Enricho Nkhoma et al., 2021 [[8](#_ENREF_8)] | Jul 20 | n=6 | PubMed, Embase, CENTRAL, Web of Science, Scopus and ProQuest |
| Effect of mobile applications on blood pressure control and their development in China: a systematic review and meta-analysis | Han et al., 2020 [[9](#_ENREF_9)] | Aug 19 | n=4 | PubMed, Embase, China national knowledge infrastructure database, and China biology medicine database |
| Effectiveness of smartphone application-based self-management interventions in patients with type 2 diabetes: a systematic review and meta-analysis of randomized controlled trials | He et al., 2021 [[10](#_ENREF_10)] | Jan 21 | n=8 | PubMed, Web of Science, Embase, Cochrane Central Register of Controlled Trials, Google Scholar, China National Knowledge Infrastructure (CNKI), Wanfang, and Sinomed |
| Do Mobile Phone Applications improve glycemic control (HbA1c) in the self-management of Diabetes? A systematic review, meta-analysis, and GRADE of 14 Randomized Trials | Hou et al., 2016 [[11](#_ENREF_11)] | Jun 15 | n=5 | Medline, CINAHL, Cochrane Library, Web of Science, and Embase |
| Mobile phone applications and self-management of diabetes: A systematic review with meta-analysis, meta-regression of 21 randomized trials and GRADE | Hou et al., 2018 [[12](#_ENREF_12)] | May 2017 | n=5 | Medline, CINAHL, Cochrane Library, Web of Science, and Embase |
| Improving glycemic control in type 2 diabetes using mobile applications and e-coaching: a mixed treatment comparison network meta-analysis | Hyun et al., 2021 [[13](#_ENREF_13)] | Oct 2020 | n=7 | PubMed, Web of Science, Cochrane Central Register of Controlled Trials, CINAL, Koreamed, KMbase, and ScienceOn |
| The effectiveness of user-focusedmobile health applications in paediatric chronic disease management: A systematic review | Karatas et al., 2021 [[14](#_ENREF_14)] | Nov 20 | n=10 | Cumulative Index to Nursing and Allied Health Literature (CINAHL), PubMed, the Cochrane Central Register of Controlled Trials (CENTRAL), ELSEVIER, SAGE, Science Direct, Scopus, Taylor Francis,Web of Science, and IEEE XPLORE |
| The Association Between Smartphone App–Based Self-monitoring of Hypertension-Related Behaviors and Reductions in High Blood Pressure: Systematic Review and Meta-analysis | Kassavou et al., 2022 [[15](#_ENREF_15)] | Aug 21 | n=7 | MEDLINE via Ovid, Embase via Ovid, Web of Science, PsycINFO, Scopus, CINAHL, and the Cochrane Central Register of Controlled Trials (CENTRAL) |
| Effectiveness of Mobile App-Assisted Self-Care Interventions for Improving Patient Outcomes in Type 2 Diabetes and/or Hypertension: Systematic Review and Meta-Analysis of Randomized Controlled Trials | Liu et al., 2020 [[16](#_ENREF_16)] | Jan 19 | n=4 | MEDLINE, Cochrane Library, EMBASE, and CINAHL Plus |
| The Effectiveness of Smartphone Apps for Lifestyle Improvement in Noncommunicable Diseases: Systematic Review and Meta-Analyses | Lunde et al., 2018 [[17](#_ENREF_17)] | Feb 17 | n=5 | EMBASE, MEDLINE, CINAHL, Academic Research Premier, and Cochrane Reviews and Trials |
| Mobile Health Applications and Medication Adherence of Patients With Hypertension: A Systematic Review and Meta-Analysis | Mikulski et al., 2022 [[18](#_ENREF_18)] | Jul 20 | n=3 | Scopus, MEDLINE, and BVSalud |
| Effectiveness of Mobile Applications on Medication Adherence in Adults with Chronic Diseases: A Systematic Review and Meta-Analysis | Peng et al., 2020 [[19](#_ENREF_19)] | Dec 2018 | n=5 | MEDLINE, EMBASE, CINAHL Plus, Cochrane Central Register of Controlled Trials, and Web of Science |
| Effect of smartphone apps on glycemic control in young patients with type 1 diabetes: A meta-analysis | Pi et al., 2023 [[20](#_ENREF_20)] | Feb 23 | n=3 | PubMed, Embase, and the Cochrane |
| The Effectiveness of Self-Management Mobile Phone and Tablet Apps in Long-term Condition Management:A Systematic Review | Whitehead et al., 2016 [[21](#_ENREF_21)] | 2016 | n=5 | PubMed, Embase, EBSCO databases, the Cochrane Library, and The Joanna Briggs Institute Library |
| Mobile app-based interventions to support diabetes self-management: a systematic review of randomized controlled trials to identify functions associated with glycemic efficacy | Wu et al., 2017 [[22](#_ENREF_22)] | May 2016 | n=5 | MEDLINE, EMBASE, the Cochrane Central Register of Controlled Trials, the Chinese Biomedical Literature Database, and ClinicalTrials.gov |
| The Efficacy of Mobile Phone Apps for Lifestyle Modification in Diabetes: Systematic Review and Meta-Analysis | Wu et al., 2019 [[23](#_ENREF_23)] | May 2018 | n=5 | Cochrane Central Register of Controlled Trials, MEDLINE, Embase, CINAHL, and PsycINFO |
| The Effect of Smartphone App-Based Interventions for Patients with Hypertension: Systematic Review and Meta-Analysis | Xu et al., 2020 [[24](#_ENREF_24)] | May 2020 | n=4 | MEDLINE, EMBASE, PubMed, and Cochrane Library |

List of excluded RCTs

| **Primary  Author, Year [Reference]** | **Eligibility criteria: English full text** | **Eligibility criteria: RCT** | **Eligibility criteria: Population (DM/HTN)** | **Eligibility criteria: health app-based intervention** | **Eligibility criteria: effectiveness/ health outcomes** | **Eligibility criteria: Reports at least 1 PROGRESS-Plus** | **Included (Yes/No)** | **Comments** |
| --- | --- | --- | --- | --- | --- | --- | --- | --- |
| Block et al., 2015 [[25](#_ENREF_25)] | Yes | Yes | No |  |  | Yes | No | Not all DM or HTN |
| Buis et al., 2017 [[26](#_ENREF_26)] | Yes | Yes | Yes | No |  | Yes | No | Not health app; SMS |
| Cho et al., 2009 [[27](#_ENREF_27)] | Yes | Yes | Yes | No | Yes | Yes | No | Not health app; Diabetes phone (mobile phone containing a device to measure blood glucose) + online portal |
| Choi et al., 2019 [[28](#_ENREF_28)] | Yes | Yes | No | Yes | Yes | Yes | No | Not all DM or HTN; diabetes 20% of participants |
| Del Rosario et al., 2018 [[29](#_ENREF_29)] | Yes | Yes | No | Yes | Yes | Yes | Yes | Device connected to smartphone |
| Eyles et al., 2017 [[30](#_ENREF_30)] | Yes | Yes | No |  |  | Yes | No | Not all DM or HTN; some have cardiovascular diseases |
| Faridi et al., 2008 [[31](#_ENREF_31)] | Yes | Yes | Yes | No | Yes | Yes | No | Not health app |
| Fukuoka et al., 2015 [[32](#_ENREF_32)] | Yes | Yes | No |  |  | Yes | No | Not all DM or HTN; Prediabetes |
| Greenwood et al., 2015 [[33](#_ENREF_33)] | Yes | Yes | Yes | No | Yes | Yes | No | Not health app; Care innovations guide (online portal) + tablet |
| Hansen et al., 2017 [[34](#_ENREF_34)] | Yes | Yes | Yes | No | Yes | Yes | No | Not health app; video calls + glucometer connected by Bluetooth/USB to tablet |
| Ho et al., 2013 [[35](#_ENREF_35)] | Yes | No |  |  |  | Yes | No | Not RCT |
| Holmen et al., 2016 [[36](#_ENREF_36)] | Yes | No | Yes | No | Yes | Yes | No | Not health app + not RCT; cross-sectional study design of a previous RCT |
| Karhula et al., 2015 [[37](#_ENREF_37)] | Yes | Yes | No | Yes | Yes | Yes | No | Not all DM or HTN; Diabetes and CVD |
| Kennelly et al., 2018 [[38](#_ENREF_38)] | Yes | Yes | No |  |  | Yes | No | Not all DM or HTN; Population with high BMI 🡪 to decrease incidence of GD |
| Kim et al, 2016 [[39](#_ENREF_39)] | Yes | No | Yes | Yes | Yes | Yes | No | Secondary analysis of RCT |
| Kumar et al., 2018 [[40](#_ENREF_40)] | Yes | Yes | Yes | No |  | Yes | No | Not health app; SMS |
| Lunde et al., 2020 [[41](#_ENREF_41)] | Yes | Yes | No |  |  | Yes | No | Not all DM or HTN; Hypertension 50% |
| Najafi Ghezeljeh et al., 2018 [[42](#_ENREF_42)] | Yes | Yes | Yes | No |  | Yes | No | Not health app; Telegram app |
| Ni et al., 2018 [[43](#_ENREF_43)] | Yes |  | No |  |  | Yes | No | Not health app; WeChat |
| Petrella et al., 2014 [[44](#_ENREF_44)] | Yes | Yes | No | Yes | Yes | Yes | No | Not all DM or HTN; includes dyslipidemia |
| Plotnikoff et al., 2017 [[45](#_ENREF_45)] | Yes | Yes | No | Yes |  | Yes | No | Not all DM or HTN |
| Prabarakhan et al., 2019 [[46](#_ENREF_46)] | Yes | Yes | No | Yes | Yes | Yes | No | DM and/or HTN population but the users of the app are the doctors and nurses |
| Quinn et al., 2008 [[47](#_ENREF_47)] | Yes | Yes | Yes | No |  | Yes | No |  |
| Rodríguez-Idígoras et al., 2009 [[48](#_ENREF_48)] | Yes | Yes | Yes | No | Yes | Yes | No | Not health app; Teleassistance system: mobile phone + webpage + call center. No app mentioned to be used on the phone |
| Rosario et al., 2018 [[29](#_ENREF_29)] | Yes | Yes | No | No | Yes | Yes | No | Not health app + not all DM or HTN |
| Santo et al., 2019 [[49](#_ENREF_49)] | Yes | Yes | No | Yes |  | Yes | No | Not all DM or HTN |
| Seto et al., 2009 [[50](#_ENREF_50)] | Yes | No |  |  |  | Yes | No | Not RCT; conference paper includes 2 trials: 1 doesn't have PROGRESS-Plus, 1 is not RCT |
| Spring et al., 2017 [[51](#_ENREF_51)] | Yes | Yes | No |  |  | Yes | No | Not all DM or HTN |
| Takenga et al., 2014 [[52](#_ENREF_52)] | Yes | Yes | Yes | No |  | No | No | Not health app |
| Tian et al., 2015 [[53](#_ENREF_53)] | Yes | Yes | No |  |  | Yes | No | Not all DM or HTN |
| van der Weegen et al., 2015 [[54](#_ENREF_54)] | Yes | Yes | No |  |  | Yes | No | Not all DM or HTN |
| Widmer et al., 2017 [[55](#_ENREF_55)] | Yes | No |  |  |  | Yes | No |  |
| Xu et al., 2021 [[56](#_ENREF_56)] | Yes | Yes | Yes | No |  | Yes | No | WeChat + wearable device |
| Yoo et al., 2009 [[57](#_ENREF_57)] | Yes | Yes | Yes | No | Yes | Yes | No | Not health app; glucose measuring device attached to phone to transmit glucose readings + website |

**Notes:** Studies highlighted in yellow were considered borderline ineligible. Reasons for their exclusion are provided in the Comments column.

Abbreviations: app: application; BMI: body-mass index; CVD: cardiovascular diseases; DM: diabetes mellitus; GD: gestational diabetes; HTN: hypertension; RCT: randomized control trial.

**References**

1. Al-Arkee S, Mason J, Lane DA, Fabritz L, Chua W, Haque MS, et al. Mobile Apps to Improve Medication Adherence in Cardiovascular Disease: Systematic Review and Meta-analysis. J Med Internet Res. 2021;23(5):e24190; doi:10.2196/24190.

2. Amalindah D, Winarto A, Rahmi A. Effectiveness of Mobile App-Based Interventions to Support Diabetes Self-Management: A Systematic Review. Jurnal Ners. 2020;15:9-18; doi:10.20473/jn.v15i1Sp.18897.

3. Armitage LC, Kassavou A, Sutton S. Do mobile device apps designed to support medication adherence demonstrate efficacy? A systematic review of randomised controlled trials, with meta-analysis. BMJ Open. 2020;10(1):e032045; doi:10.1136/bmjopen-2019-032045.

4. Bonoto BC, de Araujo VE, Godoi IP, de Lemos LL, Godman B, Bennie M, et al. Efficacy of Mobile Apps to Support the Care of Patients With Diabetes Mellitus: A Systematic Review and Meta-Analysis of Randomized Controlled Trials. JMIR Mhealth Uhealth. 2017;5(3):e4; doi:10.2196/mhealth.6309.

5. Cai X, Qiu S, Luo D, Wang L, Lu Y, Li M. Mobile Application Interventions and Weight Loss in Type 2 Diabetes: A Meta-Analysis. Obesity (Silver Spring). 2020;28(3):502-9; doi:10.1002/oby.22715.

6. Cui M, Wu X, Mao J, Wang X, Nie M. T2DM Self-Management via Smartphone Applications: A Systematic Review and Meta-Analysis. PLoS One. 2016;11(11):e0166718; doi:10.1371/journal.pone.0166718.

7. El-Gayar O, Ofori M, Nawar N. On the efficacy of behavior change techniques in mHealth for self-management of diabetes: A meta-analysis. J Biomed Inform. 2021;119:103839; doi:10.1016/j.jbi.2021.103839.

8. Enricho Nkhoma D, Jenya Soko C, Joseph Banda K, Greenfield D, Li YJ, Iqbal U. Impact of DSMES app interventions on medication adherence in type 2 diabetes mellitus: systematic review and meta-analysis. BMJ Health Care Inform. 2021;28(1); doi:10.1136/bmjhci-2020-100291.

9. Han H, Guo W, Lu Y, Wang M. Effect of mobile applications on blood pressure control and their development in China: a systematic review and meta-analysis. Public Health. 2020;185:356-63; doi:10.1016/j.puhe.2020.05.024.

10. He Q, Zhao X, Wang Y, Xie Q, Cheng L. Effectiveness of smartphone application-based self-management interventions in patients with type 2 diabetes: A systematic review and meta-analysis of randomized controlled trials. J Adv Nurs. 2022;78(2):348-62; doi:10.1111/jan.14993.

11. Hou C, Carter B, Hewitt J, Francisa T, Mayor S. Do Mobile Phone Applications Improve Glycemic Control (HbA1c) in the Self-management of Diabetes? A Systematic Review, Meta-analysis, and GRADE of 14 Randomized Trials. Diabetes Care. 2016;39(11):2089-95; doi:10.2337/dc16-0346.

12. Hou C, Xu Q, Diao S, Hewitt J, Li J, Carter B. Mobile phone applications and self-management of diabetes: A systematic review with meta-analysis, meta-regression of 21 randomized trials and GRADE. Diabetes Obes Metab. 2018;20(8):2009-13; doi:10.1111/dom.13307.

13. Hyun MK, Lee JW, Ko SH, Hwang JS. Improving Glycemic Control in Type 2 Diabetes Using Mobile Applications and e-Coaching: A Mixed Treatment Comparison Network Meta-Analysis. J Diabetes Sci Technol. 2022;16(5):1239-52; doi:10.1177/19322968211010153.

14. Karatas N, Kaya A, Isler Dalgic A. The effectiveness of user-focused mobile health applications in paediatric chronic disease management: A systematic review. J Pediatr Nurs. 2022;63:e149-e56; doi:10.1016/j.pedn.2021.09.018.

15. Kassavou A, Wang M, Mirzaei V, Shpendi S, Hasan R. The Association Between Smartphone App-Based Self-monitoring of Hypertension-Related Behaviors and Reductions in High Blood Pressure: Systematic Review and Meta-analysis. JMIR Mhealth Uhealth. 2022;10(7):e34767; doi:10.2196/34767.

16. Liu K, Xie Z, Or CK. Effectiveness of Mobile App-Assisted Self-Care Interventions for Improving Patient Outcomes in Type 2 Diabetes and/or Hypertension: Systematic Review and Meta-Analysis of Randomized Controlled Trials. JMIR Mhealth Uhealth. 2020;8(8):e15779; doi:10.2196/15779.

17. Lunde P, Nilsson BB, Bergland A, Kvaerner KJ, Bye A. The Effectiveness of Smartphone Apps for Lifestyle Improvement in Noncommunicable Diseases: Systematic Review and Meta-Analyses. J Med Internet Res. 2018;20(5):e162; doi:10.2196/jmir.9751.

18. Mikulski BS, Bellei EA, Biduski D, De Marchi ACB. Mobile Health Applications and Medication Adherence of Patients With Hypertension: A Systematic Review and Meta-Analysis. American Journal of Preventive Medicine. 2022;62(4):626-34; doi:<https://doi.org/10.1016/j.amepre.2021.11.003>.

19. Peng Y, Wang H, Fang Q, Xie L, Shu L, Sun W, et al. Effectiveness of Mobile Applications on Medication Adherence in Adults with Chronic Diseases: A Systematic Review and Meta-Analysis. J Manag Care Spec Pharm. 2020;26(4):550-61; doi:10.18553/jmcp.2020.26.4.550.

20. Pi L, Shi X, Wang Z, Zhou Z. Effect of smartphone apps on glycemic control in young patients with type 1 diabetes: A meta-analysis. Front Public Health. 2023;11:1074946; doi:10.3389/fpubh.2023.1074946.

21. Whitehead L, Seaton P. The Effectiveness of Self-Management Mobile Phone and Tablet Apps in Long-term Condition Management: A Systematic Review. J Med Internet Res. 2016;18(5):e97; doi:10.2196/jmir.4883.

22. Wu X, Guo X, Zhang Z. The Efficacy of Mobile Phone Apps for Lifestyle Modification in Diabetes: Systematic Review and Meta-Analysis. JMIR Mhealth Uhealth. 2019;7(1):e12297; doi:10.2196/12297.

23. Wu Y, Yao X, Vespasiani G, Nicolucci A, Dong Y, Kwong J, et al. Mobile App-Based Interventions to Support Diabetes Self-Management: A Systematic Review of Randomized Controlled Trials to Identify Functions Associated with Glycemic Efficacy. JMIR Mhealth Uhealth. 2017;5(3):e35; doi:10.2196/mhealth.6522.

24. Xu H, Long H. The Effect of Smartphone App-Based Interventions for Patients With Hypertension: Systematic Review and Meta-Analysis. JMIR Mhealth Uhealth. 2020;8(10):e21759; doi:10.2196/21759.

25. Block G, Azar KM, Romanelli RJ, Block TJ, Hopkins D, Carpenter HA, et al. Diabetes Prevention and Weight Loss with a Fully Automated Behavioral Intervention by Email, Web, and Mobile Phone: A Randomized Controlled Trial Among Persons with Prediabetes. J Med Internet Res. 2015;17(10):e240; doi:10.2196/jmir.4897.

26. Buis L, Hirzel L, Dawood RM, Dawood KL, Nichols LP, Artinian NT, et al. Text Messaging to Improve Hypertension Medication Adherence in African Americans From Primary Care and Emergency Department Settings: Results From Two Randomized Feasibility Studies. JMIR Mhealth Uhealth. 2017;5(2):e9; doi:10.2196/mhealth.6630.

27. Cho JH, Lee HC, Lim DJ, Kwon HS, Yoon KH. Mobile communication using a mobile phone with a glucometer for glucose control in Type 2 patients with diabetes: as effective as an Internet-based glucose monitoring system. J Telemed Telecare. 2009;15(2):77-82; doi:10.1258/jtt.2008.080412.

28. Choi BG, Dhawan T, Metzger K, Marshall L, Akbar A, Jain T, et al. Image-Based Mobile System for Dietary Management in an American Cardiology Population: Pilot Randomized Controlled Trial to Assess the Efficacy of Dietary Coaching Delivered via a Smartphone App Versus Traditional Counseling. JMIR Mhealth Uhealth. 2019;7(4):e10755; doi:10.2196/10755.

29. Rosario MBD, Lovell NH, Fildes J, Holgate K, Yu J, Ferry C, et al. Evaluation of an mHealth-Based Adjunct to Outpatient Cardiac Rehabilitation. IEEE J Biomed Health Inform. 2018;22(6):1938-48; doi:10.1109/JBHI.2017.2782209.

30. Eyles H, McLean R, Neal B, Jiang Y, Doughty RN, McLean R, et al. A salt-reduction smartphone app supports lower-salt food purchases for people with cardiovascular disease: Findings from the SaltSwitch randomised controlled trial. Eur J Prev Cardiol. 2017;24(13):1435-44; doi:10.1177/2047487317715713.

31. Faridi Z, Liberti L, Shuval K, Northrup V, Ali A, Katz DL. Evaluating the impact of mobile telephone technology on type 2 diabetic patients' self-management: the NICHE pilot study. J Eval Clin Pract. 2008;14(3):465-9; doi:10.1111/j.1365-2753.2007.00881.x.

32. Fukuoka Y, Gay CL, Joiner KL, Vittinghoff E. A Novel Diabetes Prevention Intervention Using a Mobile App: A Randomized Controlled Trial With Overweight Adults at Risk. Am J Prev Med. 2015;49(2):223-37; doi:10.1016/j.amepre.2015.01.003.

33. Greenwood DA, Blozis SA, Young HM, Nesbitt TS, Quinn CC. Overcoming Clinical Inertia: A Randomized Clinical Trial of a Telehealth Remote Monitoring Intervention Using Paired Glucose Testing in Adults With Type 2 Diabetes. J Med Internet Res. 2015;17(7):e178; doi:10.2196/jmir.4112.

34. Hansen CR, Perrild H, Koefoed BG, Zander M. Video consultations as add-on to standard care among patients with type 2 diabetes not responding to standard regimens: a randomized controlled trial. Eur J Endocrinol. 2017;176(6):727-36; doi:10.1530/EJE-16-0811.

35. Ho GY, Cattermole GN, Chan SS, Smith BE, Graham CA, Rainer TH. Noninvasive transcutaneous Doppler ultrasound-derived hemodynamic reference ranges in Chinese adolescents. Pediatr Crit Care Med. 2013;14(5):e225-32; doi:10.1097/PCC.0b013e3182772f78.

36. Holmen H, Wahl A, Torbjornsen A, Jenum AK, Smastuen MC, Ribu L. Stages of change for physical activity and dietary habits in persons with type 2 diabetes included in a mobile health intervention: the Norwegian study in RENEWING HEALTH. BMJ Open Diabetes Res Care. 2016;4(1):e000193; doi:10.1136/bmjdrc-2016-000193.

37. Karhula T, Vuorinen AL, Raapysjarvi K, Pakanen M, Itkonen P, Tepponen M, et al. Telemonitoring and Mobile Phone-Based Health Coaching Among Finnish Diabetic and Heart Disease Patients: Randomized Controlled Trial. J Med Internet Res. 2015;17(6):e153; doi:10.2196/jmir.4059.

38. Kennelly MA, Ainscough K, Lindsay KL, O'Sullivan E, Gibney ER, McCarthy M, et al. Pregnancy Exercise and Nutrition With Smartphone Application Support: A Randomized Controlled Trial. Obstet Gynecol. 2018;131(5):818-26; doi:10.1097/AOG.0000000000002582.

39. Kim JY, Wineinger NE, Steinhubl SR. The Influence of Wireless Self-Monitoring Program on the Relationship Between Patient Activation and Health Behaviors, Medication Adherence, and Blood Pressure Levels in Hypertensive Patients: A Substudy of a Randomized Controlled Trial. Journal of medical Internet research. 2016;18(6):e116; doi:10.2196/jmir.5429.

40. Kumar D, Raina S, Sharma SB, Raina SK, Bhardwaj AK. Effectiveness of randomized control trial of mobile phone messages on control of fasting blood glucose in patients with type-2 diabetes mellitus in a Northern State of India. Indian J Public Health. 2018;62(3):224-6; doi:10.4103/ijph.IJPH_199_17.

41. Lunde P, Bye A, Bergland A, Grimsmo J, Jarstad E, Nilsson BB. Long-term follow-up with a smartphone application improves exercise capacity post cardiac rehabilitation: A randomized controlled trial. Eur J Prev Cardiol. 2020;27(16):1782-92; doi:10.1177/2047487320905717.

42. Najafi Ghezeljeh T, Sharifian S, Nasr Isfahani M, Haghani H. Comparing the effects of education using telephone follow-up and smartphone-based social networking follow-up on self-management behaviors among patients with hypertension. Contemp Nurse. 2018;54(4-5):362-73; doi:10.1080/10376178.2018.1441730.

43. Ni Z, Liu C, Wu B, Yang Q, Douglas C, Shaw RJ. An mHealth intervention to improve medication adherence among patients with coronary heart disease in China: Development of an intervention. Int J Nurs Sci. 2018;5(4):322-30; doi:10.1016/j.ijnss.2018.09.003.

44. Petrella RJ, Stuckey MI, Shapiro S, Gill DP. Mobile health, exercise and metabolic risk: a randomized controlled trial. BMC Public Health. 2014;14:1082; doi:10.1186/1471-2458-14-1082.

45. Plotnikoff RC, Wilczynska M, Cohen KE, Smith JJ, Lubans DR. Integrating smartphone technology, social support and the outdoor physical environment to improve fitness among adults at risk of, or diagnosed with, Type 2 Diabetes: Findings from the 'eCoFit' randomized controlled trial. Prev Med. 2017;105:404-11; doi:10.1016/j.ypmed.2017.08.027.

46. Prabhakaran D, Jha D, Prieto-Merino D, Roy A, Singh K, Ajay VS, et al. Effectiveness of an mHealth-Based Electronic Decision Support System for Integrated Management of Chronic Conditions in Primary Care: The mWellcare Cluster-Randomized Controlled Trial. Circulation. 2019;139(3):380-91; doi:10.1161/CIRCULATIONAHA.118.038192.

47. Quinn CC, Clough SS, Minor JM, Lender D, Okafor MC, Gruber-Baldini A. WellDoc mobile diabetes management randomized controlled trial: change in clinical and behavioral outcomes and patient and physician satisfaction. Diabetes Technol Ther. 2008;10(3):160-8; doi:10.1089/dia.2008.0283.

48. Rodriguez-Idigoras MI, Sepulveda-Munoz J, Sanchez-Garrido-Escudero R, Martinez-Gonzalez JL, Escolar-Castello JL, Paniagua-Gomez IM, et al. Telemedicine influence on the follow-up of type 2 diabetes patients. Diabetes Technol Ther. 2009;11(7):431-7; doi:10.1089/dia.2008.0114.

49. Santo K, Singleton A, Rogers K, Thiagalingam A, Chalmers J, Chow CK, et al. Medication reminder applications to improve adherence in coronary heart disease: a randomised clinical trial. Heart. 2019;105(4):323-9; doi:10.1136/heartjnl-2018-313479.

50. Seto E, Istepanian RS, Cafazzo JA, Logan A, Sungoor A. UK and Canadian perspectives of the effectiveness of mobile diabetes management systems. Annu Int Conf IEEE Eng Med Biol Soc. 2009;2009:6584-7; doi:10.1109/IEMBS.2009.5333998.

51. Spring B, Pellegrini CA, Pfammatter A, Duncan JM, Pictor A, McFadden HG, et al. Effects of an abbreviated obesity intervention supported by mobile technology: The ENGAGED randomized clinical trial. Obesity (Silver Spring). 2017;25(7):1191-8; doi:10.1002/oby.21842.

52. Takenga C, Berndt RD, Musongya O, Kitero J, Katoke R, Molo K, et al. An ICT-Based Diabetes Management System Tested for Health Care Delivery in the African Context. Int J Telemed Appl. 2014;2014:437307; doi:10.1155/2014/437307.

53. Tian M, Ajay VS, Dunzhu D, Hameed SS, Li X, Liu Z, et al. A Cluster-Randomized, Controlled Trial of a Simplified Multifaceted Management Program for Individuals at High Cardiovascular Risk (SimCard Trial) in Rural Tibet, China, and Haryana, India. Circulation. 2015;132(9):815-24; doi:10.1161/CIRCULATIONAHA.115.015373.

54. van der Weegen S, Verwey R, Spreeuwenberg M, Tange H, van der Weijden T, de Witte L. It's LiFe! Mobile and Web-Based Monitoring and Feedback Tool Embedded in Primary Care Increases Physical Activity: A Cluster Randomized Controlled Trial. J Med Internet Res. 2015;17(7):e184; doi:10.2196/jmir.4579.

55. Widmer RJ, Allison TG, Lennon R, Lopez-Jimenez F, Lerman LO, Lerman A. Digital health intervention during cardiac rehabilitation: A randomized controlled trial. Am Heart J. 2017;188:65-72; doi:10.1016/j.ahj.2017.02.016.

56. Xu Y, Xu L, Zhao W, Li Q, Li M, Lu W, et al. Effectiveness of a WeChat Combined Continuous Flash Glucose Monitoring System on Glycemic Control in Juvenile Type 1 Diabetes Mellitus Management: Randomized Controlled Trial. Diabetes Metab Syndr Obes. 2021;14:1085-94; doi:10.2147/DMSO.S299070.

57. Yoo HJ, Park MS, Kim TN, Yang SJ, Cho GJ, Hwang TG, et al. A Ubiquitous Chronic Disease Care system using cellular phones and the internet. Diabet Med. 2009;26(6):628-35; doi:10.1111/j.1464-5491.2009.02732.x.
