# Supplementary material for: Consideration of inequalities in effectiveness trials of mHealth applications – a systematic assessment of studies from an umbrella review
Source: Int J Equity Health. 2024 Sep 11;23:181. doi: 10.1186/s12939-024-02267-4 (PMC11389088; doi:10.1186/s12939-024-02267-4)
Supplement: Supplementary file 7 — Supplementary Material 7 [file 12939_2024_2267_MOESM7_ESM.docx]

Additional File 7. Summary of study designs, indications, sample size, and duration of follow-up

**Summary of Sample Sizes, Study Design and Follow-Up**

The sample size at baseline ranged between 17 and 480 patients (mean = 120, median = 85). The sum of all participants after removing duplicate publications was 7796, of which 5815 were in diabetes articles, 1622 were in hypertension articles, and 359 were in articles addressing both diseases.

Of the included 65 RCTs, 37 were parallel group efficacy designs, 22 pilot or feasibility studies (two cluster randomized, and one cross-over), four cluster randomized, one cross-over, and one stepped-wedge design. Most included two study arms, seven had three arms, two had four arms, and one had five arms.

Follow-up durations ranged between 1 and 18 months with a mean of 5.85 and a median of 6.00 months.

*Table 1. Details of study designs, indications, sample sizes, and follow up timepoints*

| **Primary Author, Year** | **Study design (Study Name)** | **Number of Arms** | **Indication/ Disease** | **Sample Size at Baseline Total (IG; CG)** | **Sample size ITT  Total (IG; CG)^a^** | **Follow-up Time Points (months)^b^** |
| --- | --- | --- | --- | --- | --- | --- |
| Agarwal et al., 2019 [80] | Pragmatic multi-center RCT | 2 | T2DM | 223 (110; 113) | N/A | **3**, 6 |
| Alanzi et al., 2018 [106] | Pilot RCT | 2 | T2DM | 20 (10; 10) | N/A | 6 |
| Alfonsi et al., 2020 [81] | Pilot RCT | 2 | T1DM | 46 (23; 23) | N/A | 3 |
| Alonso-Dominguez et al., 2019 [94] | RCT (EMID study) | 2 | T2DM | 204 (102; 102) | N/A | 3, **12** |
| Alonso-Domínguez et al., 2019 [95] | RCT (EMID study)  *(Original study Alonso-Domínguez et al, 2019 [94])* | 2 | T2DM | 204 (102; 102)^d^ | N/A | 3, **12** |
| Alotaibi et al., 2016 [107] | Pilot RCT | 2 | T2DM | 20 (10; 10) | N/A | 6 |
| Anzaldo-Campos et al., 2016 [52] | Open-label RCT (DWT) | 3 | T2DM | 301 (102; 99; 100) | N/A | 4, **10** |
| Baron et al., 2017 [85] | RCT | 2 | T1 and/or T2DM | 81 (45; 36) | N/A | 3, **9** |
| Baron et al., 2017 [86] | RCT (*Original study Baron et al, 2017 [85])* | 2 | T1 and/or T2DM | 81 (45; 36)^d^ | N/A | **3, 9** |
| Bee et al., 2016 [99] | Pilot RCT | 2 | T2DM | 66 (33; 33) | N/A | 1.5, 3, 4.5**, 6** |
| Bender et al., 2017 [55] | Pilot RCT | 2 | T2DM | 45 (22; 23) | N/A | **3**, 4, 6 |
| Berndt et al., 2014 [115] | Pilot RCT | 2 | T1DM | 68 (34; 34) | N/A | 1 |
| Boels et al., 2019 [108] | Open two-arm multicenter RCT (TRIGGER) | 2 | T2DM | 230 (115; 115) | N/A | 6 |
| Brath et al., 2013 [114] | Pilot crossover RCT | 2 | T2DM and/or hypertension | 77 | N/A | 5 |
| Castelnuovo et al., 2011 [98] | RCT (TECNOB) | 2 | T2DM | 34 (17; 17) | N/A | **3, 6, 12** |
| Castensøe-Seidenfaden et al., 2018 [118] | Open parallel RCT | 2 | T1DM | 151 (76; 75) | N/A | 2, 7, **12** |
| Chandler et al., 2019 [56] | RCT (SMASH) | 2 | Hypertension | 54 (26; 28) | N/A | 1, 3, **6**, 9 |
| Chao et al., 2019 [71] | RCT | 2 | T2DM | 121 (59; 62) | N/A | 18 |
| Charpentier et al., 2011 [101] | Open label, parallel group RCT | 3 | T1DM | 180 (59; 60; 61) | N/A | 6 |
| Chatzakis et al., 2019 [119] | prospective, open‐label RCT | 2 | T1DM | 80 (40; 40) | N/A | 3, 6, 12 |
| Di Bartolo et al., 2017 [97] | multicenter, open-label RCT (i-NewTrend) | 2 | T1DM | 182 (92; 90) | 182 (92; 90) | 3, 6, 12 |
| Dorsch et al., 2020 [57] | Pilot RCT | 2 | Hypertension | 50 (24; 26) | N/A | 2 |
| Drion et al., 2015 [109] | Prospective open-label RCT | 2 | T1DM | 63 (32; 31) | N/A | 3 |
| Dugas et al., 2018 [58] | Pilot RCT | 5 | T2DM | 27 (22 [T1: 5, T2:5, T3: 6, T4: 6]; 5) | N/A | 3.25 |
| Franc et al., 2019 [102] | RCT | 3 | T2DM | 189 (63; 64; 62) | N/A | **4,** 13 |
| Frias et al., 2017 [59] | Open-label, prospective, cluster pilot RCT | 3 | Hypertension/T2DM | 109 (40; 40; 29) | N/A | **1**, 3 |
| Garg et al., 2017 [60] | Pilot RCT- single-center, prospective, open-label | 2 | T1DM | 100 (50; 50) | N/A | **3, 6** |
| Gong, E et al., 2020 [110] | Open-label RCT | 2 | T2DM | 187 (93; 94) | 187 (60; 78) | 6, **12** |
| Gong, K et al., 2020 [72] | Multicenter RCT | 2 | Hypertension | 480 (240; 240) | N/A | 6 |
| Goyal et al., 2017 [84] | RCT | 2 | T1DM | 92 (46; 46) | N/A | 3, 6, 9, **12** |
| Gunawardena et al., 2019 [47] | RCT | 2 | Diabetes | 67 (35; 32) | N/A | 3, **6** |
| Hilliard., 2020 [61] | Pilot feasibility and acceptability RCT | 2 | T1DM | 80 (55; 25) | N/A | 3.5 |
| Holmen et al., 2014 [103] | Prospective RCT (RENEWING HEALTH) | 3 | T2DM | 151 (51; 50; 50) | N/A | 4, **12** |
| Hsu et al., 2016 [62] | RCT | 2 | T2DM | 40 (20; 20) | N/A | 3 |
| Huang et al., 2019 [100] | Feasibility RCT | 2 | T2DM | 51 (25; 26) | N/A | 3 |
| Istepanian et al., 2009 [87] | Parallel group RCT | 2 | Diabetes | 137 (72; 65) | N/A | 9 |
| Kardas et al., 2016 [116] | Feasibility prospective parallel-arm RCT (COMODITY2) | 2 | T2DM | 60 (30; 30) | N/A | 1.5 |
| Kim et al., 2019 [91] | Multicenter open-label RCT | 2 | T2DM | 172 (90; 82) | N/A | 3, **6** |
| Kirwan et al., 2013 [111] | RCT | 2 | T1DM | 72 (36; 36) | N/A | 3, 6, **9** |
| Klee et al., 2018 [113] | Double-crossover one-center study RCT | 2 | T1DM | 55 (28; 27) | N/A | 3 |
| Kleinman et al., 2017 [48] | Open labelled RCT | 2 | T2DM | 90 (44; 46) | 90 (44; 46) | 3, **6** |
| Kusnanto et al., 2019 [49] | RCT | 2 | T2DM | 65 (30; 35) | N/A | 3 |
| Lakshminarayan et al., 2018 [63] | Pilot RCT | 2 | Hypertension + stroke survivors | 56 (34; 22) | N/A | 3 |
| Lee et al., 2017 [53] | Cluster RCT (Making Ramadan Fasting A Safer Experience Study [MRFAST]) | 2 | T2DM | 85 (45; 40) | N/A | 3 |
| Lee et al., 2020 [92] | Open-label RCT | 2 | T2DM | 72 (41; 31) | N/A | 3, **6** |
| Logan et al., 2012 [82] | Prospective, randomized, open, blinded primary end-point trial | 2 | Diabetes + hypertension | 110 (55; 55) | N/A | 12 |
| Márquez Contreras et al., 2019 [96] | Observational prospective cluster RCT | 2 | Arterial hypertension | 154 (77; 77) | N/A | 6, **12** |
| Morawski et al., 2018 [64] | RCT | 2 | Hypertension | 411 (209; 202) | 411 (209; 202) | 1, 2, **3** |
| Nagrebetsky et al., 2013 [88] | Feasibility RCT | 2 | T2DM | 17 (8; 9) | N/A | 3, **6** |
| Or et al., 2016 [73] | Pilot RCT | 2 | T2DM and/or hypertension | 63 (33; 30) | N/A | 1, 2, **3** |
| Orsama et al., 2013 [112] | RCT | 2 | T2DM | 56 (27; 29) | N/A | 10 |
| Persell et al., 2020 [65] | RCT | 2 | Hypertension | 297 (144; 153) | 297 (144; 152) | 6 |
| Quinn et al., 2011 [66] | Cluster RCT | 4 | T2DM | 213 (38; 33; 80; 62) | N/A | 3, 6, 9, **12** |
| Quinn et al., 2014 [67] | Cluster RCT *(Original study Quinn et al, 2011 [66])* | 2^c^ | T2DM | 118 (62; 56) | N/A | 12 |
| Quinn et al., 2016 [68] | Cluster RCT *(Original study Quinn et al, 2011 [66])* | 2^c^ | T2DM | 118 (62; 56)^d^ | 118 (62; 56) | 12 |
| Rossi et al., 2010 [89] | Open label, multicenter, parallel group RCT | 2 | T1DM | 130 (67; 63) | N/A | 3, **6** |
| Rossi et al., 2013 [90] | Open label, multicenter, parallel group RCT  *(Original study Rossi et al, 2010 [89])* | 2 | T1DM | 127 (63; 64) | N/A | 3, **6** |
| Sarfo et al., 2018 [50] | Pilot cluster RCT  *(Interim Results of Sarfo et al, 2019 [51])* | 2 | Hypertension + stroke survivors | 60 (30; 30) | N/A | 3 |
| Sarfo et al., 2019 [51] | Pilot cluster RCT | 2 | Hypertension + stroke survivors | 60 (30; 30) | N/A | 3, 6, **9** |
| Skrøvseth et al., 2015 [105] | Stepped wedge RCT | 2 | T1DM | 30 (15; 15) | N/A | 2 |
| Sun et al., 2019 [74] | RCT | 2 | T2DM | 91 (47; 44) | N/A | **3, 6** |
| Torbjørnsen et al., 2014 [104] | RCT (Renewing Health)  *(Part 1/short-term results of Holmen et al, 2014 [103])* | 3 | T2DM | 151 (51; 50; 50)^d^ | N/A | **4,** 12 |
| Waki et al., 2014 [117] | RCT | 2 | T2DM | 54 (27; 27) | N/A | 3 |
| Wang et al., 2018 [69] | Pilot RCT | 3 | T2DM + overweight/ obese | 26 (11; 9; 9) | N/A | 3, **6** |
| Wang et al., 2019 [74] | RCT | 2 | T2DM | 120 (60; 60) | N/A | 6 |
| Wayne et al., 2015 [83] | Pragmatic RCT | 2 | T2DM | 131 (67; 64) | N/A | 3, **6** |
| Yang et al., 2020 [93] | Cluster RCT | 2 | T2DM | 247 (150; 97) | 239 (145; 94) | 3 |
| Yu et al., 2019 [76] | Pilot RCT | 4 | T2DM | 185 (45; 48; 45; 47) | N/A | 3, **6** |
| Zha et al., 2020 [70] | Pilot RCT | 2 | Hypertension | 30 (15;15) | N/A | 3, **6** |
| Zhai et al., 2020 [77] | Single-center, open-label, prospective RCT | 2 | T2DM | 120 (60; 60) | N/A | **3, 6** |
| Zhang et al., 2019 [78] | Single-center, open-labeled, prospective RCT | 3 | Diabetes | 234 (78; 78; 78) | N/A | 3, **6** |
| Zhou et al., 2016 [79] | Pilot RCT | 2 | Diabetes | 100 (50; 50) | N/A | 3 |

**Notes**: Reference numbers refer to the reference numbers in the main text.

Abbreviations: CG: control group; IG: intervention group; ITT: intention-to-treat; N/A: not applicable; RCT: randomized control trial; T1DM: Type1 diabetes mellitus; T2DM: Type 2 diabetes mellitus.

^a^ Sample size for ITT analysis was only extracted for the studies that had subgroup analyses with PROGRESS-Plus characteristics.

^b^ Studies with multiple follow-up points have the main follow-up point in bold.

^c^ Not all arms of the original study were taken in the additional publications.
^d^ Sample sizes excluded from the total sum sample size reported in the manuscript.
