# Supplementary material for: Consideration of inequalities in effectiveness trials of mHealth applications – a systematic assessment of studies from an umbrella review
Source: Int J Equity Health. 2024 Sep 11;23:181. doi: 10.1186/s12939-024-02267-4 (PMC11389088; doi:10.1186/s12939-024-02267-4)
Supplement: Supplementary file 8 — Supplementary Material 8 [file 12939_2024_2267_MOESM8_ESM.docx]

Additional File 8. Summary of app used, the intervention, and the comparison Groups

### **Summary of Apps Characteristics and Comparison Groups**

The apps had various types of uses. Most of the functions were recording and monitoring blood glucose or blood pressure measurements, while several provided education about the disease or self-care, reminders for taking medication, or calculated insulin doses.

One study had a unique intervention where the participants ingested a sensor attached to the pills they took, and the sensor sent to a wearable patch how many pills were taken, which was reflected in the app to monitor medication adherence[1]. Another app displayed some socioeconomic factors that could affect a patient’s health such as education and SEP[2].

Most of the comparison groups (n= 51) were usual or standard care, some had a paper or logbook alternative to the app, and some had information or advice on managing the disease or improving lifestyle and one study had another app as a comparison[3].

*Table 1. Summary of apps, interventions, and comparisons in the studies*

| **Primary Author, Year** | **Indication/ Disease** | **App Name; Device Used // Additional Device** | **App brief  description** | **Intervention  description** | **Comparison  description** |
| --- | --- | --- | --- | --- | --- |
| Agarwal et al., 2019 [80] | T2DM | BlueStar; smartphone | - Virtual coaching - Entering diabetes measurements - Receiving feedback | - Virtual coaching: deliver customized, evidence-based real time messages aiming to improve motivation, behavior, and education. | - Usual care for 3 months while being on a waiting list, then using the app for 3 months |
| Alanzi et al., 2018 [106] | T2DM | SANAD; smartphone | - Collecting BG measurements - Providing educational material - Behavioral change | - The intervention system consisted of 1) a diabetes management module, 2) a social networking module, and 3) a cognitive behavioral therapy module | - Normal medical treatment and management by staff of the center |
| Alfonsi et al., 2020 [81] | T1DM | iSpy; smartohone | - Carbohydrate counting, photo taking, portion sizing, and food identification. | - iSpy participants were instructed to use the app at their discretion and when they thought its use would be beneficial. | - Usual care |
| Alonso-Dominguez et al., 2019 [94, 95] | T2DM | EVIDENT II; smartphone | - Counting the number of daily steps - Inputting food intake, daily exercise data, and physical activity - Daily assessment of the physical activity levels - Providing recommendations - Tailoring diet-based information | - Multifactorial intervention consisting of five heart-healthy walks (once per week), use of the app, a diet workshop, and counselling on physical activity and diet | - Physical activity and diet counselling |
| Alotaibi et al., 2016 [107] | T2DM | SAED; smartphone | - Transferring BG measurements - Receiving educational messages - Receiving recommendations about risk factors - Lifestyle advice and/or routine changes in pharmacological treatment | - The SAED system compromises: 1) mobile patient/healthcare provider component, 2) intelligent diabetes management component, and 3) diabetes educational module component. | - Usual care |
| Anzaldo-Campos et al., 2016 [52] | T2DM | Brew; cell phone | - Offering interactive surveys - Short educational videos - Brochures - Reminders of surveys - Reading text messages | - Arm 1 (PD-INT): care management and a peer-led group education component. - Arm 2 (PD-TE): In addition to the PD-INT, receiving a glucose meter, test strips, and a cell phone. Patients were asked to measure their fasting and postprandial glucose 2 times/day during the first month and 2 days/week during the second month. | - Standard care |
| Baron et al., 2017 [85, 86] | T1 and/or T2DM | MTH; mobile phone | - Store and transmit BG, BP, physical activity, insulin dose, and weight measurements. - Graphical feedback - Feedback on out-of-range clinical readings - Education on life-style changes | - Regular BG and BP self-monitoring routine. - The MTH nurses viewed the data transmitted to the server and provided feedback on out-of-range clinical readings, education on lifestyle changes, and insulin titration. | - Standard care |
| Bee et al., 2016 [99] | T2DM | Diabetes Pal; smartphone | - Entering FPG measurements - Calculating insulin dose - Offering “Hypoglycemia Guide” containing text instructions on recognizing and managing hypoglycemia | - Entering FPG readings daily into the app and the app recommended an insulin dose. The study staff could monitor remotely the readings and report any concerns to the endocrinologists | - Paper logbooks and written instructions |
| Bender et al., 2017 [55] | T2DM | Fitbit app; smartphone/ tablet //  + Fitbit accelerometer | - Diary - Self-reporting daily food/calorie intake - Documenting body weight weekly | - Using the Fitbit accelerometer to monitor real-time physical activity steps and using the associated app with a diary to document food/caloric intake and weight. - Joining a private Facebook group created by the study researchers for virtual social support, coaching, and weekly education topics posted by the staff. | - Waitlist receiving only the Fitbit accelerometer and training for daily wear |
| Berndt et al., 2014 [115] | T1DM | Mobil Diab (mDiab); smartphone | - Transferring diabetes-related records (BG measurements, nutrition and drugs information) - Recording mobility and sport-related information - Graphical representation of trends | - Sending (recommended at least 3 times/day) the diabetes-related records to the central platform. | - Conventional therapy |
| Boels et al., 2019 [108] | T2DM | (-); smartphone | - Providing patients with diabetes self-management education and support delivered as text messages on dietary habits, physical activity, prevention of hypoglycemia, and glucose control - The text messages contained specific goals, healthy lifestyle information and challenges | - Receiving app triggers and care as usual according to the national diabetes guidelines | - Care as usual |
| Brath et al., 2013 [114] | T2DM and/or hypertension | (-); NFC enabled mobile phones// + electronic medication blisters | - Reading and automatically transferring measurements - Sending medication reminders to patients - Presenting the data to the study physician numerically and graphically via browser-based user interface | - Patients had to touch the user ID card with the mobile phone (to launch and login), read out the event data by touching the blister with the mobile phone, wait until data was transmitted successfully and the app closed automatically. - The system recorded the timestamp and number of pills taken whenever a patient took out a pill from a blister. | - Standard medication blisters, routine care and handwritten medication intake diaries |
| Castelnuovo et al., 2011 [98] | T2DM | METADIETA; mobile phone | - Contacting the dietitian - Visually display the food choices (frequency and portions), food diary - Reminders of the videoconference appointments with the clinicians and the days when to fill-in the questionnaires | - Attending for a month an inpatient intensive program with individualized medical care, diet therapy, physical training and short psychological counseling. - Using a weight-loss website, a dietary software installed into the cellular phones, a web-based videoconference tool and an electronic armband measuring daily steps and energy expenditure. | - A 1-month inpatient intensive program that involved individualized medical care, diet therapy, physical training and brief psychological counseling. |
| Castensøe-Seidenfaden et al., 2018 [118] | T1DM | YWD; smartphone/ tablet | - Contacting the healthcare provider - Writing notes - Offering information about the diabetes department - Chatting with peers - Offering information to count carbohydrates - Offering information about type 1 diabetes and tips - Providing parents with information about supporting their teens - Reminders for self-management | - The participants and their parents received the same version of YWD except for the Chat Room, which was only available for the young participants. - They were advised to use YWD as a stand-alone resource and in collaboration with their parents and health care providers. | - Usual outpatient care |
| Chandler et al., 2019 [56] | Hypertension | SMASH; smartphone // + medication tray | - Providing instructions for correct placement of BP cuff - Sending alerts to take a BP reading - Countdown timer - Feedback of systolic and diastolic BP measurements and heart rate levels - Cumulative table of BP progress reports - Automated tailored motivational/social reinforcement messages based on medication adherence | - Measuring BP every 3 days using the Bluetooth monitor paired with SMASH while instantly receiving audio and visual feedback of their BP reading and heart rate levels. - Receiving medication trays which provided a series of reminder signals to take medications (blinking light, intermittent chime, automated SMS/phone call). | - Enhanced standard care |
| Chao et al., 2019 [71] | T2DM | IPMF platform; smartphone/ iPad | - Monitoring health status, education, health behavior change - Displaying the factors that may influence patient's health in daily life such as family history, education, income, lifestyle and stress | - An IPMF interactive system was used to observe the patient-insight factor and encompassed disease history, family history, and relevant characteristics. | - Traditional care |
| Charpentier et al., 2011 [101] | T1DM | Diabeo; smartphone | - Bolus calculator with validated algorithms considering self-monitoring plasma glucose level before meals, carbohydrate counts, and planned physical activity | - Arm 1: receiving a smartphone loaded with the Diabeo software and face-to-face follow-up visits were planned for month 3 and month 6. - Arm 2: receiving a smartphone with the Diabeo software and teleconsultations by telephone call every 2 weeks. | - Paper logbook |
| Chatzakis et al., 2019 [119] | T1DM | Euglyc; smartphone | - A database of 7000 foods and food products in Greek cuisine as well as the corresponding amount of carbohydrates and lipids > amount of carbohydrates and lipids contained in a meal are calculated automatically by the application > calculation of insulin dose + physical activity component | - Use the application for the calculation of the bolus insulin dose they were injected. | - Normal method of insulin dose calulation |
| Di Bartolo et al., 2017 [97] | T1DM | iBGStar™ Diabetes Manager App (DM App); smartphone | - When iBGStar™ is connected to the iPhone with the iBGStar™ Diabetes Manager App launched, the new blood glucose readings will be downloaded from the meter to the Application. | - Experimental glucose meter and telemedicine system | - Traditional glucose meter |
| Dorsch et al., 2020 [57] | Hypertension | LowSalt4Life; smartphone | - Sending messages for behavioral change - Showing participant-selected alternatives to their high-sodium foods - Suggesting a list of low-sodium meal options at restaurants - Offering to search restaurant menus prioritized by low sodium content - Offering to scan the universal product codes of the grocery store items to find similar food options containing lower sodium contents | - Participants selected alternatives to their 5 high-sodium foods and geotagged the places in which these foods were consumed or purchased. - Real time messages were provided to promote behavioral change when a participant entered a grocery store, restaurant, or home | - Usual care |
| Drion et al., 2015 [109] | T1DM | DBEES; smartphone | - Entering diabetes-related self-care data (BG values, carbohydrate intake, medication, physical exercise, and notes) | - Using the DBEES app and a personal web portal linked to it | - Paper diary |
| Dugas et al., 2018 [58] | T2DM | DiaSocial; tablet //  + FitBit | - Providing patients with specific, salient health behavior goals through features like gamification | - Encouraged to use the app daily and to earn points by recording the progress in achieving better diabetes selfcare goals through managing glucose level, exercise, diet, and medication adherence. - Social interaction with team mates online through the app | - Usual care |
| Franc et al., 2019 [102] | T2DM | Diabeo-BI; smartphone | - Adaptation and calculation of evening bolus insulin doses according to the fasting BG values entered - Interpretation of pre- and postprandial glycaemia | - Arm 1: Diabeo‐BI combined with short telephone consultations (~5 min) - Arm 2: interactive voice response system and short telephone consultations (~5 min) | - Standard care |
| Frias et al., 2017 [59] | Hypertension/T2DM | Proteus Discover; smartphone/ tablet // + proteus ingestible sensor + wearable sensor patch | - Receiving data transmitted from a wearable sensor patch which was transmitted from the proteus ingestible sensor (activated after swallowing and sends signal with a specific code to the patch) - Reminders of medication schedule | - Receiving the same intervention but for different durations: arm 1 for 4 weeks and arm 2 for 12 weeks. - The medicines were co-encapsulated with an ingestible sensor that was activated after swallowing, in addition to wearing an adhesive wearable sensor patch. - Investigators made medication changes and provided patient education/counseling as needed. | - Usual care |
| Garg et al., 2017 [60] | T1DM | Diabetes Manager App; smartphone // + iBGStar glucose meter | - Receiving glucose measurements - Connection with the research center | - Contacting the research site if BG reading was below 60 or above 300 mg/dL using the app, which sends a notification text from the iPhone to the provider. - Participants instructed to take BG measurements 3 times/day. - Using iBGStar with the Diabetes Manager App, iPhone can function as a glucometer | - Routine care, communication provided via telephone/email/fax, and receiving Accu-Chek Nanometers and training on how to use them. |
| Gong, E et al., 2020 [110] | T2DM | MDC; smartphone | - Personalized support, monitoring and coaching via an embodied conversational agent, Laura, through a series of modules covering BG monitoring, healthy eating, physical activity, medication taking, and foot care | - The program had 5 components: the MDC app, a printed user guide, the MDC website, an optional BG meter with Bluetooth, and some interactions with the program coordinator. - Using the app weekly to complete online modules. - Additionally, participants were also encouraged to regularly access the user guide, the website, and the discussion forums on diabetes self-management. | - Routine diabetes self-care |
| Gong, K et al., 2020 [72] | Hypertension | Yan Fu; smartphone | - Personal record - BP measurement storage and reminder - Consultation about HTN - Emergency contact - Allowing relatives and doctors to monitor the patient - Health evaluation and advice | - Receiving scientific information and suggestions about hypertension. - Uploading BP at least once daily and recording medication taken in the app. Otherwise, the app would send reminder messages. - If the BP was abnormal, the app would send warnings to measure the BP again after taking a rest and would recommend seeing a doctor. | - Measure BP using the same sphygmomanometer like the intervention group and record on paper daily |
| Goyal et al., 2017 [84] | T1DM | bant; smartphone | - Automatic Data Transfer, elogbook, trends, reward system, social media communty (banter), personal health record | - Intervention group received an iPhone 4S loaded with bant, blood glucose meter, and a Bluetooth adapter that allowed for wireless transmission of data from the blood glucose meter to bant. | - Usual care |
| Gunawardena et al., 2019 [47] | Diabetes | SGM; smartphone | - Reminders for BG measurement, medication, exercise, meal - Demographics collection - Storage and visualization of BG measurements - Bolus insulin calculation | - Participants were instructed to download and use the app | - Existing BG monitoring methods and diabetes care |
| Hilliard., 2020 [61] | T1DM | Type 1 Doing Well; smartphone | - Psychoeducation, feedback on teen's strength, reminder, text message library | - Parents used the app to record positive T1D-related actions (i.e., diabetes strengths) their teen engaged in each day. The app summarized their ratings each week and presented a personalized weekly strengths summary for each family. Parents had access to videos in the app to learn about recognizing strengths and how to give effective, developmentally appropriate praise. The app also included a library of text messages that parents could use to praise their teen. | - Usual medical care |
| Holmen et al., 2014 [103] & Torbjørnsen et al., 2014 [104] | T2DM | FTA self-management system; mobile phone | - Diary - Food habit registration - Physical activity registration - Personal goal setting - Providing general information | - Arm 1: In addition to usual care, participants received a mobile phone with the FTA self-management system. - Arm 2: In addition to the mobile phone, FTA system, and usual care, the participants in the received health counseling for the first 4 months | - Usual care |
| Hsu et al., 2016 [62] | T2DM | CollaboRhythm software platform; tablet | - Self-efficacy in diabetes care through self-tracking tools - Shared decision-making interfaces for subjects and healthcare professionals - Streamlined communications tools - Co-creation of a diabetes care plan with the healthcare professional - Secure text messages and virtual visits (audio, video, shared screen control) instead of office visits | - Receiving a tablet (with the medication regimen and the initial insulin dose preset on it), a glucose meter (connected to the tablet) - Self-monitoring BG once a day - Regular and when needed virtual interactions with the healthcare professionals. | - Standard care |
| Huang et al., 2019 [100] | T2DM | Medisafe; smartphone | - Medication scheduling - Sending reminders - Tracking adherence - Data sharing - Medication adherence assessments | - Using the app on for managing medications for 12 weeks by setting the medication schedule and reminder. - Adding the research group as a “Medfriend” to observe their medication adherence. | - Usual care |
| Istepanian et al., 2009 [87] | Diabetes | (-); mobile phone | - Receiving and transmitting BG measurements - Sending alerts to remind of measurement time | - Self-measuring capillary blood sugar and data was sent from the phone to a server where the research staff clinicians checked the recordings. - The clinicians sent letters with details of the amalgamated readings and treatment recommendations to the patients and their general practitioners. | - Did not transmit their readings and received care with their usual doctor in the outpatient and/or primary care setting |
| Kardas et al., 2016 [116] | T2DM | SmartHub; smartphone | - Receiving and transmitting measurements | - Using the COMMODITY12 system which was a smartphone and wirelessly connected sensors (a glucometer, BP reader and scale, a sensor of ECG, heart rhythm, and respiratory movements, an accelerometer, an adherence monitor). | - Standard care |
| Kim et al., 2019 [91] | T2DM | mDiabetes; smartphone + Bluetooth glucometer | - Glucose measurements recording - Sending feedback - Calculating insulin dose | - Receiving a Bluetooth glucometer and an activity tracker. - Using mDiabetes | - Paper logbook (plogbook) to record glucose measurement and received a Bluetooth glucometer |
| Kirwan et al., 2013 [111] | T1DM | Glucose Buddy; smartphone | - Recording BG levels, insulin dosages, other medications, diet (food item in grams), and physical activities (minutes) - Visualization of measurements in graph and export this information via email | - The data entered in the app was reviewed weekly by a Certified Diabetes Educator via a Web interface. - Participants received at least 1 personalized text-message per week for the first 6 months. | - Usual care |
| Klee et al., 2018 [113] | T1DM | Webdia; mobile device | - Insulin bolus calculator - List of nutrients and carbohydrate content - Option to save frequent meals | - Using Webdia as frequently as possible for 3 months. - BG measurements were reviewed monthly by diabetologists, and suggested treatment changes were sent to the participants by email. | - Usual care for 3 months |
| Kleinman et al., 2017 [48] | T2DM | Gather Health; mobile phone | - Reminders - Data visualization - Ongoing support to increase self-care behaviors and facilitate collaborative care decisions | - Using Gather Health platform: Gather app for patients, a Web portal, and an app for providers. - The app sent daily automated reminders to complete tasks. - Following up on out-of-range BG values to detect problems. | - Usual care |
| Kusnanto et al., 2019 [49] | T2DM | DM-Calendar; mobile phone | The app is for patients with diabetes living alone and has 4 pillars:   - Helping with blood sugar control - Education program - Nutrition therapy - Physical activity - Reminders for blood sugar check | - Using the app | - Media leaflets containing information about diet program, physical activity and blood sugar control. |
| Lakshminarayan et al., 2018 [63] | Hypertension + stroke survivors | Withings; smartphone | - Transmitting BP measurements to database to be checked by medical staff | - Patient education delivered by a nurse coordinator. - Self-measured BP monitoring - Rapid transfer of BP readings to an inter-professional care team and responsive medication adjustment and feedback. | - Education on the importance of hypertension control.   Advice to self-monitor BP daily and share with primary care provider at clinic visits.   - Encouragement to follow up with primary care provider based on follow-up schedule advised by them. |
| Lee et al., 2017 [53] | T2DM | (-); smartphone | - Upload glucose measurements | - Uploading the glucose readings to an online portal to be viewed by the researcher. - Sending reminders through the web portal to measure glucose level. | - Usual routine practice |
| Lee et al., 2020 [92] | T2DM | Healthynote; smartphone | - Storing BG measurements, BP, body weight - Receiving messages from healthcare professionals about diabetes or medications | - Entering self-monitored BG level, dietary record, exercise, BP, medication record, and body weight into the app. - Receiving messages from healthcare professionals | - Usual care |
| Logan et al., 2012 [82] | Diabetes + hypertension | (-); smartphone | - Transmitting measurements - Sending selfcare messages | - The home BP device with built-in Bluetooth was paired with a smartphone to automatically transmit the readings to a central server for processing and storage. - Patients received instructions when BP was outside the target range to take additional BP readings. - Nonadherence to the BP measurement schedule triggered an automated voice message sent to the patients’ home telephone to check the smartphone for a message | - The home BP device without built-in Bluetooth capability |
| Márquez Contreras et al., 2019 [96] | Arterial hypertension | AlerHTA; mobile phone | - Health education - Medication reminders - Record personal data - Recommend BP levels as objectives - Set reminder alarms, a calendar of appointments or events, and record the results of the BP measurement. | - For medication monitoring, the participants received a digital record in the form of a microchip in the lid of the drug container that automatically controls its opening and registers the time and date. - Measuring how many times the container was opened, assuming that one pill was taken with each opening. | - Usual care |
| Morawski et al., 2018 [64] | Hypertension | Medication adherence Improvement Support App For Engagement—Blood Pressure (MedISAFE-BP; smartphone | - Medication reminders - Generation of adherence reports - Tracking of BP and other biometric measurements - Peer support | - Downloading and using the Medisafe app to assist with medication. | - No app |
| Nagrebetsky et al., 2013 [88] | T2DM | (-); mobile phone | - BG measurements transmission - Graphical representation of the readings - Recommendations on medication dosage based on glucose readings | - Receiving a mobile telephone and a BG meter with a Bluetooth cradle and performing at least six BG tests per week, of which at least three were required to be fasting. | - Usual care and monthly telephone calls about diet, physical activity, medication changes |
| Or et al., 2016 [73] | T2DM and/or hypertension | (-); tablet | - Measuring, automatic recording, and monitoring BP, BG, and pulse - Generating graphs and tables containing the vital signs records - Video-based educational materials about how to measure BG and BP, what diet to follow, and what exercises to perform | - Patients received a tablet computer and a 2-in-1 BG and BP monitor that enabled them to measure, automatically record, and monitor their BG, BP, and pulse. | - 2-in-1 BG and BP monitor for self-monitoring and a logbook for recording the vital signs measured and the dates and times of the measurements |
| Orsama et al., 2013 [112] | T2DM | Monica; mobile phone | - Uploading of health parameter measurements (BP, weight, physical activity, and BG) - Displaying graphs reflecting the uploaded data in relation to individual target values - Behavioral skills feedback message to support patient self-care | - Receiving a mobile phone with the app to report BP, weight, physical activity, and BG). - Receiving a personal health record account to view medical records and remote data reports that were uploaded using the app | - Standard medical care |
| Persell et al., 2020 [65] | Hypertension | HPCP coaching app; smartphone // + HBPM | Conversational artificial intelligence using cognitive behavioral therapy techniques.   - Provide education, coaching to promote healthy behaviors (diet, physical activity, medication adherence, BP measurement, sleep, and stress management) - Provide medication reminders - Ask about adherence - Provide coaching around barriers to adherence | - Receiving BP monitor - Self-monitoring of BP - Using HPCP coaching app | - Receiving BP monitor - Self-monitoring of BP - Omron app: BP tracking app and home BP monitoring |
| Quinn et al., 2011 [66] | T2DM | MDMA; mobile phone // + web portal + glucose meter | - Enter diabetes self-care data (BG values, carbohydrate intake, medications, and other diabetes management information) - Receiving automated, real-time educational/ behavioral/motivational messages specific to the entered data | - Group 3 (CPP): data only view group allowed providers to access unanalyzed patient data. - Group 4 (CPDS): providers had access to analyzed patient data linked to standards of care and evidence-based guidelines. | - Group 1: usual care - Group 2: coach only |
| Quinn et al., 2014, 2016 [67, 68] | T2DM | MDMA; mobile phone // + web portal + glucose meter | - Enter diabetes self-care data (BG values, carbohydrate intake, medications, and other diabetes management information) - Receiving automated, real-time educational/ behavioral/motivational messages specific to the entered data | - Group 4 (CPDS): providers had access to analyzed patient data linked to standards of care and evidence-based guidelines. | - Usual care |
| Rossi et al., 2010 [89, 90] | T1DM | DID; mobile phone | - Automatic carbohydrate/insulin bolus calculator - Communication tool between patient and physician via text messages | - Attending a course on the use of DID lasting up to 2 weeks. - The course was provided as an outpatient program of 3 meetings with the physician and/or dietitian. | - Standard carbohydrate counting |
| Sarfo et al., 2019 [50, 51] | T1DM | (-); smartphone | - BP readings transfer - Monitoring medication adherence | - Receiving a Bluetooth built-in device and smartphone for monitoring and reporting BP measurements and medication intake. - Tailored motivational text messages were delivered based on the levels of adherence to the medication intake protocol. | - SMS messages about healthy lifestyle behaviors but not about medication adherence |
| Skrøvseth et al., 2015 [105] | T1DM | Diabetes diary (DD); mobile phone | - Generating periodical graphs about BG - Displaying trends - Matching situation to inform on insulin injections | - Using the app | - Not using the app for 8 weeks, then using it for 10 weeks |
| Sun et al., 2019 [74] | T2DM | (-); mobile phone | - Uploading the glucometer data, which was transmitted automatically to the medical server | - Using the app-based diet management software to input daily dietary intake. - The dietitian received the daily dietary record of each patient via the app. - Receiving monthly dietary recommendations from the dietitian | - Received free glucometer and dietary guidance from dietitians during face-to-face meetings at baseline and at the end of the study |
| Waki et al., 2014 [117] | T2DM | DialBetics; smartphone | - Transferring BG, BP, body weight, and pedometer counts. - Evaluating data automatically - if each reading satisfies guideline requirements, then immediately sending results to each patient’s smartphone - Communicating advice on lifestyle modification, matched to the patient’s input about food and exercise - Dietary evaluation: sending the nutritional value of meals | - Receiving smartphone, glucometer, pedometer, and scale all connected to Dialbetics | - Usual care |
| Wang et al., 2018 [69] | T2DM + overweight/ obese | LoseIt! app + Diabetes Connect app; smartphone | - LoseIt!: self-monitoring of diet, physical activity, and weight - Diabetes Connect app:Transmission of data from glucometer and weight scale | - Using apps to monitor meals, calories, fat goals, self-monitoring of carbohydrate intake, BG, weight, exercise, calories burnt. | - Paper diaries given to monitor meals, calories, fat goals, self-monitoring of carbohydrate intake, BG, weight, exercise, calories burnt. - Usual diabetes care and education on glycemic control and weight loss |
| Wang et al., 2019 [74] | T2DM | (-); mobile phone | - Uploading daily BG levels, diet status, insulin and related drug use, and physical activity - Sending tailored information from the physician about glucose monitoring, insulin injection reminders, personalized diets, and personalized exercise guidelines - Options to request health advice using voice, picture, video, or text messages - Providing scientific information on diabetes, diet, sports, and medication - Providing personalized summaries and suggestions on BG control - Sharing experiences with other diabetes patients | - Interacting with the doctor through the app - Receiving BG monitoring reminders - Receiving alerts when BG rose to abnormal levels - Dietary recording in the mobile application interface. - Exercise guidance records developed based on fluctuations of BG levels. - Exercise intensity measured with a motion bracelet, and when the amount of exercise was too high or insufficient, the app gave a warning. | - Nurses provided patients with detailed health guidance before discharge covering BG monitoring, insulin injections, personalized diets, and exercise regimens. |
| Wayne et al., 2015 [83] | T2DM | CWP; smartphone | - Health-related goal setting and progress monitoring - Key metrics tracking (BG measurements, exercise frequency/duration/intensity, food intake via photo journaling, and mood) - Communication with health coach | - Co-monitoring of the patient’s input by a health coach and giving immediate attention to events of desirable progress, relapse, and resistance. | - Health coach support in selecting and progressing toward goals without access to a (study-provided) mobile phone or CWP |
| Yang et al., 2020 [93] | T2DM | Hicare smart K; mobile phone // + glucometer  + strips | - Uploading of BG measurements and feedback | - Educating participants on managing BG, BP, lipid profile, and body weight - Uploading daily self-managed BG results using the app for 3 months. | - No app |
| Yu et al., 2019 [76] | T2DM | Diabetes-Carer; smartphone | - Diabetic education - Self-management - Patient community - Real-time communication between patients and clinicians | - In the groups B and D (self-managed BG), each patient received a BG meter and strips. - In the MPA groups C and D, each patient was asked to install the app. | - Usual care |
| Zha et al., 2020 [70] | Hypertension | iHealth MyVitals; smartphone | - Tracking and analyzing key health vital measurements and receiving instant feedback | - Measuring BP and pulse rate with a Bluetooth synchronization system, in addition to standard care | - Standard care |
| Zhai et al., 2020 [77] | T2DM | YuTangYiHu; smartphone | - Reading BG measurements - Diet advice - Emotional management - Medication guidance | - BG data were compiled into charts in the doctor’s version of the app and viewed by the outpatient doctor in addition to conventional diabetic treatment | - Conventional diabetic treatment |
| Zhang et al., 2019 [78] | Diabetes | Welltang; smartphone | - Education - Self-management (including records of self-monitoring BG, diet, exercise, medication, body weight, and other diabetes data) - Patient community - Communication between patients and clinicians | - Groups B (app-self-management) and C (app interactive management) were asked to regularly record the glucose results in Welltang - Group B learned diabetes-related knowledge and skills by using the app. They received care from 1 clinician from the study staff. - Group C, in addition to app self-management, received interactive management online. | - Record their glucose results in a logbook |
| Zhou et al., 2016 [79] | Diabetes | Welltang; smartphone | - Information on diet, exercise, medicine, BG monitoring, and the latest guidelines for diabetes care - Entering BG values, carbohydrate intake, medications, and other diabetes management information) - Communication with clinicians - Feedback on the BG levels, the target goals, and individualized medication regimens | - Using the Welltang app | - Usual care once a month and not using Welltang |

**Notes**: Studies written in grey are additional published articles of the original study. Reference numbers refer to the reference numbers in the main text.

Abbreviations: app: application; BG: blood glucose; BP: blood pressure; CPDS: coach primary care provider portal with decision support; CPP: coach primary care provider portal; CWP: Connected Wellness Platform; FPG: fasting plasma glucose; FTA: Few Touch Application; HBPM: home blood pressure monitor; HPCP: hypertension personal control program; IPMF: interactive personalized management framework; MDC: My Diabetes Coach; MDMA: Mobile diabetes management system; MPA: mobile phone application; MTH: Mobile Telehealth; PD-INT: Project Dulce–only intervention; PD-TE: Project Dulce technology-enhanced intervention; T1DM: type 1 diabetes mellitus; T2DM: type 2 diabetes mellitus; YWD: Young with Diabetes

References

1. Frias J, Virdi N, Raja P, Kim Y, Savage G, Osterberg L. Effectiveness of Digital Medicines to Improve Clinical Outcomes in Patients with Uncontrolled Hypertension and Type 2 Diabetes: Prospective, Open-Label, Cluster-Randomized Pilot Clinical Trial. J Med Internet Res. 2017;19(7):e246; doi:10.2196/jmir.7833.

2. Chao DY, Lin TM, Ma W-Y. Enhanced Self-Efficacy and Behavioral Changes Among Patients With Diabetes: Cloud-Based Mobile Health Platform and Mobile App Service. JMIR Diabetes. 2019;4(2):e11017; doi:10.2196/11017.

3. Persell SD, Peprah YA, Lipiszko D, Lee JY, Li JJ, Ciolino JD, et al. Effect of Home Blood Pressure Monitoring via a Smartphone Hypertension Coaching Application or Tracking Application on Adults With Uncontrolled Hypertension: A Randomized Clinical Trial. JAMA Netw Open. 2020;3(3):e200255; doi:10.1001/jamanetworkopen.2020.0255.
