# Supplementary material for: Consideration of inequalities in effectiveness trials of mHealth applications – a systematic assessment of studies from an umbrella review
Source: Int J Equity Health. 2024 Sep 11;23:181. doi: 10.1186/s12939-024-02267-4 (PMC11389088; doi:10.1186/s12939-024-02267-4)
Supplement: Supplementary file 9 — Supplementary Material 9 [file 12939_2024_2267_MOESM9_ESM.docx]

Additional File 9. Summary of Outcomes of the Studies

| **Primary Author,**  **Year** | **Disease** | **Outcome Type** | **Outcome(s)^a^** | **Instrument used/ Units^b^** | **Effect Size^c^  (CI / *P* value)** |
| --- | --- | --- | --- | --- | --- |
| Agarwal et al., 2019 [80] | T2DM | Primary | HbA1c | % | - |
|  |  | Secondary | Patient self-management, experience of care, and self-reported health utilization | PAID, SDSCA, EQ-5D |  |
| Alanzi et al., 2018 [106] | T2DM | Primary | HbA1c* | % | - |
|  |  | Secondary | Health awareness | DKT | - |
|  |  | Secondary | Behavioral change* | DMSES | - |
| Alfonsi et al., 2020 [81] | T1DM | Secondary | QoL | Quality of life for youth |  |
|  |  | Secondary | Self-care | Self-Care Inventory |  |
|  |  | Secondary | HbA1c* | % |  |
| Alonso-Dominguez et al., 2019 [94] | T2DM | - | Objective quantification of physical activity* | Pedometer | - |
|  |  | - | Subjective quantification of physical activity* | IPAQ-S |  |
|  |  | - | Drug use | n (%) |  |
|  |  | - | BP | mm Hg |  |
|  |  | - | BMI* | kg/m2 |  |
|  |  | - | Waist circumference* | cm |  |
|  |  | - | FBG | mg/dL |  |
|  |  | - | HbA1c | % |  |
|  |  | - | TG | mg/dL |  |
|  |  | - | TC | mg/dL |  |
|  |  | - | LDL | mg/dL |  |
|  |  | - | HDL | mg/dL |  |
| Alonso-Dominguez et al., 2019 [95] | T2DM | Primary | Mediterranean diet adherence* | Change in total score of MEDAS | - |
|  |  | Secondary | Diet* | DQI |  |
|  |  | Secondary | Drug use | n (%) |  |
|  |  | Secondary | BP | mm Hg |  |
|  |  | Secondary | BMI | kg/m2 |  |
|  |  | Secondary | FBG | mg/dL |  |
|  |  | Secondary | HbA1c | % |  |
|  |  | Secondary | TG | mg/dL |  |
|  |  | Secondary | TC | mg/dL |  |
|  |  | Secondary | LDL | mg/dL |  |
|  |  | Secondary | HDL | mg/dL |  |
| Alotaibi et al., 2016 [107] | T2DM | Primary | HbA1c* | % | - |
|  |  | Primary | Diabetes knowledge test | DKQ |  |
| Anzaldo-Campos et al., 2016 [52] | T2DM | Primary | HbA1c* | % and mmol/mol | - |
|  |  | Secondary | TC | mg/dL |  |
|  |  | Secondary | LDL | mg/dL |  |
|  |  | Secondary | HDL | mg/dL |  |
|  |  | Secondary | TG | mg/dL |  |
|  |  | Secondary | SBP | mm Hg |  |
|  |  | Secondary | DBP | mm Hg |  |
|  |  | Secondary | BMI | kg/m2 |  |
|  |  | Secondary | Self-efficacy | Spanish Diabetes Self-Efficacy |  |
|  |  | Secondary | Depression | PHQ |  |
|  |  | Secondary | Lifestyle | IMEVID |  |
|  |  | Secondary | QoL | Diabetes 39 |  |
|  |  | Secondary | Diabetes knowledge | DKQ24 |  |
| Baron et al., 2017 [85] | T1 and/or T2DM | Primary | HbA1c | % | - |
|  |  | Secondary | BP | mm Hg |  |
|  |  | Secondary | Daily insulin dose | Units |  |
|  |  | Secondary | Diabetes outpatient appointment | No. |  |
|  |  | Secondary | Health related QoL | SF |  |
|  |  | Secondary | Diabetes-specific measures | DHP-18 |  |
|  |  | Secondary | Depression symptoms | CESD-10 |  |
|  |  | Secondary | Anxiety | STAI-6 |  |
| Baron et al., 2017 [86] | T1 and/or T2DM | Primary | Self-efficacy* | heiQ, IMDSES | - |
|  |  | Primary | Diabetes self-care | SDSCA |  |
| Bee et al., 2016 [99] | T2DM | - | Change in HbA1c | % |  |
|  |  | - | Reductions in FPG | mmol/L |  |
|  |  | - | Episodes of severe hypoglycemia | n (%) |  |
| Bender et al., 2017 [55] | T2DM | Secondary | Weight change* | % | - |
|  |  | Secondary | Body weight* | kg |  |
|  |  | Secondary | BMI* | kg/m2 |  |
|  |  | Secondary | Waist circumference* | cm |  |
|  |  | Secondary | FPG* | mg/dL |  |
|  |  | Secondary | HbA1c | % |  |
|  |  | Secondary | Step counts | No. |  |
| Berndt et al., 2014 [115] | T1DM | - | Body weight | kg | - |
|  |  | - | BMI | kg/m^2^ |  |
|  |  | - | Mean amplitude of blood glucose excursions | mmol/L |  |
|  |  | - | HbA1c | % |  |
|  |  | - | Insulin dose | IU |  |
|  |  | - | Insulin dose per kg body weight | IU/kg |  |
|  |  | - | QoL | DQOLY, Diabetes Family Conflict Scale, Overall  expected self-efficacy, Diabetes Self-Efficacy Scale, Child Behavior Checklist, Youth  Self-Report, Vocabulary Test, and Test about sequence of numbers |  |
|  |  | - | Diabetes self-efficacy | DSES |  |
| Boels et al., 2019 [108] | T2DM | Primary | HbA1c | % and mmol/mol | - |
|  |  | Secondary | BMI | kg/m2 |  |
|  |  | Secondary | Presence of hypoglycemic events | No. |  |
|  |  | Secondary | Glycemic variability | Coefficient  of variation (CoV):  SD/ mean*100 |  |
|  |  | Secondary | Self-care behavior | SDSCA |  |
|  |  | Secondary | Dietary habits | Kristal’s food frequency questionnaire |  |
|  |  | Secondary | Physical activity | IPAQ |  |
|  |  | Secondary | Health status | EQ-5D-5L and visual analog scale, SF-36 |  |
|  |  | Secondary | Diabetes-dependent quality of life | ADDQOL |  |
| Brath et al., 2013 [114] | T2DM and/or HTN | Primary | Medication adherence* | Intake rate | - |
|  |  | Secondary | FPG | mg/dL |  |
|  |  | Secondary | HbA1c | % |  |
|  |  | Secondary | Body weight | kg |  |
|  |  | Secondary | BP | mm Hg |  |
|  |  | Secondary | TC | mg/dL |  |
|  |  | Secondary | LDL | mg/dL |  |
|  |  | Secondary | HDL | mg/dL |  |
| Castelnuovo et al., 2011 [98] | T2DM | Primary | Body weight | kg | - |
|  |  | Secondary | Disordered eating-related behaviors and cognitions | EDI-2 |  |
| Castensøe-Seidenfaden et al., 2018 [118] | T1DM | Primary | HbA1c* | mmol/mol or % | - |
|  |  | Secondary | Development of self-management skills | PCD; |  |
|  |  | Secondary | Healthcare providing autonomy-supportive treatment | HCCQ |  |
|  |  | Secondary | Perceived burden of diabetes-related problems | PAID-20 |  |
|  |  | Secondary | Severe hypoglycemic episodes | n (%) |  |
|  |  | Secondary | Acute diabetes-related hospitalizations | n (%) |  |
| Chandler et al., 2019 [56] | HTN | Primary | SBP* | mm Hg | - |
|  |  | Secondary | DBP | mm Hg |  |
|  |  | Secondary | Medication adherence* | MMAS |  |
| Chao et al., 2019 [71] | T2DM | Primary | Self-care knowledge and behavior change | Knowledge score | - |
|  |  | Primary | Weight | kg |  |
|  |  | Primary | BMI | kg/m^2^ |  |
|  |  | Primary | SBP | mm Hg |  |
|  |  | Primary | DBP | mm Hg |  |
|  |  | Primary | HbA1c | % |  |
| Charpentier et al., 2011 [101] | T1DM | Primary | HbA1c* | % | - |
|  |  | Secondary | Change in the HbA1c level from baseline to end point* | % |  |
|  |  | Secondary | The proportion of patients reaching the HbA1c target of below 7.5%* | % |  |
|  |  | Secondary | The change in SMPG frequency | - |  |
|  |  | Secondary | Severe hypoglycemic episodes | No. |  |
|  |  | Secondary | QoL | DQOL and DHP |  |
|  |  | Secondary | Time spent by doctors delivering care to patients | minutes |  |
|  |  | Secondary | Change in basal insulin dose | - |  |
| Chatzakis et al., 2019 [119] | T1DM | Primary | HbA1c* | % | -0.12%  *P* = 0.51 |
|  |  | Secondary | Percentage of normoglycemic, hypoglycemic and hyperglycemic events * | % | -0.014  *P* = 0.85 |
| Di Bartolo et al., 2017 [97] | T1DM | Primary | HbA1c | % | - |
|  |  | Primary | SMBG compliance | % |  |
|  |  | Secondary | percentage of patients with HbA1c ≤7.5% | % |  |
|  |  | Secondary | Additional measures of compliance with SMBG | average number of SMBG/week |  |
|  |  | Secondary | QoL | ADDQoL-19, DQOLY |  |
|  |  | Secondary | Incidence of grade 1 and grade 2 hypoglycemia. | No. |  |
| Dorsch et al., 2020 [57] | HTN | Primary | Change in dietary sodium intake* | Kawasaki, ASAQ24, FFQ, sodium screener | - |
|  |  | Secondary | Self-care | SCFLDS |  |
|  |  | Secondary | BP | mm Hg |  |
| Drion et al., 2015 [109] | T1DM | Primary | QoL | RAND-36 | - |
|  |  | Secondary | Diabetes related stress | PAID |  |
|  |  | Secondary | HbA1c | mmol/mol |  |
|  |  | Secondary | Daily frequency of SMBG | No. |  |
| Dugas et al., 2018 [58] | T2DM | Primary | HbA1c | % | - |
|  |  | Secondary | Treatment adherence | Allocated daily points when reported in the app |  |
| Franc et al., 2019 [102] | T2DM | Primary | Decrease in HbA1c* | % | - |
|  |  | Secondary | % of patients reaching HbA1c <7.0%* | % |  |
|  |  | Secondary | % of patients reaching FBG between 73 and 108 mg/dL* | % |  |
|  |  | Secondary | FBG* | mg/dL |  |
|  |  | Secondary | Pre- and postprandial BG* | mg/dL |  |
|  |  | Secondary | Changes in insulin doses* | U/kg/day |  |
|  |  | Secondary | Quality of life | DHP |  |
|  |  | Secondary | Hypoglycemic episodes | No. |  |
|  |  | Secondary | Severe hypoglycemia | No. |  |
|  |  | Secondary | Weight gain | kg |  |
| Frias et al., 2017 [59] | HTN/ T2DM | Primary | Change of SBP (week 4)* | mm Hg | - |
|  |  | Secondary | Change of SBP (week 12) | mm Hg |  |
|  |  | Secondary | Change of HbA1c (week 12) | % |  |
|  |  | Secondary | Change of DBP (week 4 and 12) | mm Hg |  |
|  |  | Secondary | Change of FPG (week 4 and 12) | mg/dL |  |
|  |  | Secondary | Proportion of participants at BP goal (week 4)* | % |  |
|  |  | Secondary | Proportion of participants at BP goal (week 12) | % |  |
|  |  | Secondary | Medication adherence | % |  |
|  |  | Secondary | TC (week 4 and 12) | mg/dL |  |
|  |  | Secondary | LDL-c (week 4 and 12) | mg/dL |  |
| Garg et al., 2017 [60] | T1DM | Primary | Hypoglycemia fear score | 27-questionnaire | - |
|  |  | Primary | Behavior and worry sub scores | Behavior (10 questions) and worry (17 questions) |  |
|  |  | Secondary | Improvement in glycemic variability indices | Basal/bolus insulin dose, average no. of SMBG/day, no. of hypoglycemic events |  |
|  |  | Secondary | HbA1c (6 months)* | % |  |
|  |  | Secondary | Hypoglycemic events | No. |  |
| Gong, E et al., 2020 [110] | T2DM | Primary | Changes in HbA1c (12 months) | % and mmol/mol | -0.04% (CI: -0.45,0.36),  *P* = 0.83 |
|  |  | Primary | HRQoL (12 months)* | AQoL-8D scale | 0.04 (CI:0.00,0.07)  *P* = 0.039 |
|  |  | Secondary | Anxiety and depressive symptoms (6 months)* | HADS | - |
|  |  | Secondary | Anxiety and depressive symptoms (12 months) | HADS |  |
|  |  | Secondary | Diabetes-specific stress (6 and 12 months) | PAID scale |  |
|  |  | Secondary | Body weight (6 and 12 months) | kg |  |
| Gong, K et al., 2020 [72] | HTN | Primary | SBP* | mm Hg | - |
|  |  | Primary | DBP* | mm Hg |  |
|  |  | Primary | Change in % of participants with controlled BP* | % |  |
|  |  | Secondary | Medication adherence* | MMS-8 |  |
| Goyal et al., 2017 [84] | T1DM | Primary | HbA1c | % | - |
|  |  | Secondary | Frequency of mild and severe hypoglycemic events | No. |  |
|  |  | Secondary | QoL | DQOLY, DFRQ, SCI |  |
| Gunawardena et al., 2019 [47] | DM | Primary | HbA1c* | % | - |
| Hilliard., 2020 [61] | T1DM | Secondary | HbA1c | % | - |
| Holmen et al., 2014 [103] | T2DM | Primary | HbA1c change | % and mmol/mol | - |
|  |  | Secondary | Self-management | heiQ |  |
|  |  | Secondary | Lifestyle changes | Dietary habits, and engagement in physical activity based on intensity, frequency, and duration |  |
|  |  | Secondary | Health-related quality of life | SF-36 |  |
|  |  | Secondary | Depressive symptoms | CES-D |  |
|  |  | Secondary | Body weight | kg |  |
| Hsu et al., 2016 [62] | T2DM | Primary | Change in HbA1c* | % | - |
|  |  | Secondary | Percentage reaching glycemic target ≤7% | % |  |
|  |  | Secondary | Frequency of hypoglycemia | No. |  |
| Huang et al., 2019 [100] | T2DM | Primary | Self-reported barriers to medication adherence* | ASK-12 + ADS | - |
|  |  | Secondary | HbA1c | % |  |
|  |  | Secondary | LDL | mmol/L |  |
|  |  | Secondary | HDL | mmol/L |  |
|  |  | Secondary | TC | mmol/L |  |
|  |  | Secondary | BMI | kg/m2 |  |
| Istepanian et al., 2009 [87] | DM | Primary | HbA1c | % | - |
| Kardas et al., 2016 [116] | T2DM | Secondary | HbA1c | % | - |
|  |  | Secondary | FBG | mg/dL |  |
|  |  | Secondary | SBP | mm Hg |  |
|  |  | Secondary | DBP | mm Hg |  |
|  |  | Secondary | Medication adherence | Proportion of doses taken vs doses prescribed |  |
|  |  | Secondary | HRQoL (health status*, anxiety and depression*) | EuroQoL-5D-5L |  |
| Kim et al., 2019 [91] | T2DM | Primary | HbA1c change (6 months)* | % | - |
|  |  | Secondary | HbA1c change (3 months) | % |  |
|  |  | Secondary | Participants achieving HbA1c <7.0%* | % |  |
|  |  | Secondary | Participants achieving HbA1c ≤6.5%* | % |  |
|  |  | Secondary | FPG* | mmol/L |  |
|  |  | Secondary | LDL | mmol/L |  |
|  |  | Secondary | HDL | mmol/L |  |
|  |  | Secondary | TG | mmol/L |  |
|  |  | Secondary | TC | mmol/L |  |
|  |  | Secondary | SBP | mm Hg |  |
|  |  | Secondary | DBP | mm Hg |  |
|  |  | Secondary | Body weight | kg |  |
|  |  | Secondary | Lean body mass | kg |  |
|  |  | Secondary | Fat mass | kg |  |
|  |  | Secondary | % of body fat* | % |  |
| Kirwan et al., 2013 [111] | T1DM | Primary | HbA1c change* | % | - |
|  |  | Secondary | Diabetes related self-efficacy | DES-SF |  |
|  |  | Secondary | Self-care behavior | SDSCA |  |
|  |  | Secondary | QoL | DQOL |  |
| Klee et al., 2018 [113] | T1DM | Primary | HbA1c change | % | - |
|  |  | Secondary | HbA1c change in patients with HbA1c >8%* | % |  |
|  |  | Secondary | QoL | DQoL |  |
|  |  | Secondary | Hypoglycemia events | Questionnaire |  |
| Kleinman et al., 2017 [48] | T2DM | Primary | Change in HbA1c (6 months)* | % | -0.7%  (-0.10, -1.37)  *P* = 0.02 |
|  |  | Secondary | Change in HbA1c (3 months) | % | - |
|  |  | Secondary | FBG (3 and 6 months) | mg/dL |  |
|  |  | Secondary | BMI (3 and 6 months) | kg/m2 |  |
|  |  | Secondary | Medication adherence (3 months) | No. (%) |  |
|  |  | Secondary | Medication adherence (6 months)* | No. (%) |  |
|  |  | Secondary | BG testing (3 and 6 months)* | No. (%) |  |
|  |  | Secondary | Communication with doctors (3 and 6 months) | No. (%) |  |
|  |  | Secondary | Diabetes self-care activities (3 and 6 months) | SDSCA |  |
|  |  | Secondary | Diabetes distress (3 and 6 months) | PAID-5 |  |
|  |  | Secondary | Self-efficacy (3 and 6 months) | PERC |  |
|  |  | Secondary | Diabetes knowledge (3 and 6 months) | RAND |  |
| Kusnanto et al., 2019 [49] | T2DM | Primary | Self-efficacy* | DMSES | - |
|  |  | Primary | HbA1c* | % |  |
|  |  | Secondary | LDL* | mg/dL |  |
|  |  | Secondary | HDL* | mg/dL |  |
|  |  | Secondary | TG* | mg/dL |  |
|  |  | Secondary | TC* | mg/dL |  |
|  |  | Secondary | Insulin* | μU/mL |  |
| Lakshminarayan et al., 2018 [63] | HTN + stroke survivors | Secondary | Hypertension controlled | n (%) | - |
|  |  | Secondary | Medication adherence | Medication compliance questionnaire |  |
|  |  | Secondary | SBP | mm Hg |  |
| Lee et al., 2017 [53] | T2DM | Primary | Rates of hypoglycemia* | n (%) | - |
|  |  | Secondary | HbA1c* | % |  |
|  |  | Secondary | Achieved HbA1c level of ≤ 7.0% | n (%) |  |
|  |  | Secondary | Lipid control (LDL, HDL, TG, TC*) | mm/DL |  |
|  |  | Secondary | QoL | EQ |  |
|  |  | Secondary | BP (SBP, DBP) | mm Hg |  |
|  |  | Secondary | Weight | kg |  |
|  |  | Secondary | Diabetes Distress | PAID |  |
|  |  | Secondary | Self-efficacy | DMSES |  |
| Lee et al., 2020 [92] | T2DM | Primary | BMI | kg/m2 | - |
|  |  | Primary | SBP | mm Hg |  |
|  |  | Primary | HbA1c | % |  |
|  |  | Primary | TC | mg/dL |  |
|  |  | Primary | TG | mg/dL |  |
|  |  | Primary | LDL | mg/dL |  |
|  |  | Primary | Self-management | SDSCA |  |
|  |  | Primary | QoL | ADDQOL |  |
|  |  | Primary | Diabetes awareness | ADS |  |
|  |  | Primary | Emotional stress derived from diabetes | PAID |  |
| Logan et al., 2012 [82] | Diabetes mellitus + HTN | Primary | Change in daytime ambulatory SBP* | mm Hg | - |
|  |  | Secondary | Change in 24h* and nighttime SBP | mm Hg |  |
|  |  | Secondary | Change in 24h*, daytime* and nighttime DBP | mm Hg |  |
|  |  | Secondary | Anxiety | HADS + ASI |  |
|  |  | Secondary | Depression* | HADS |  |
| Márquez Contreras et al., 2019 [96] | Arterial HTN | Primary | Medication adherence* | Average adherence % | - |
|  |  | Secondary | SBP* | mm Hg |  |
|  |  | Secondary | DBP* | mm Hg |  |
| Morawski et al., 2018 [64] | HTN | Primary | Medication adherence* | MMAS-8 | 0.5 (CI: 0.2, 0.7) *P* = 0.001 |
|  |  | Primary | SBP | mm Hg | -0.1 (CI: -3.2, 3.1) *P* = 0.97 |
|  |  | Secondary | Controlled BP | Less than or equal 140/90 mm Hg | - |
| Nagrebetsky et al., 2013 [88] | T2DM | Primary | HbA1c | mmol/mol | - |
|  |  | Primary | Change in oral glucose lowering medication | N (%) |  |
| Or et al., 2016 [7] | T2DM and/or HTN | Primary | HbA1c | % | - |
|  |  | Primary | FBG | mmol/dL |  |
|  |  | Primary | SBP* | mm Hg |  |
|  |  | Primary | DBP | mm Hg |  |
|  |  | Secondary | Participants’ knowledge about diabetes and hypertension | modified Michigan Diabetes Knowledge Scale |  |
| Orsama et al., 2013 [112] | T2DM | Primary | Change in HbA1c* | % | - |
|  |  | Primary | Change in SBP | mm Hg |  |
|  |  | Secondary | Reduction in body weight* | kg |  |
|  |  | Secondary | Change in DBP | mm Hg |  |
| Persell et al., 2020 [65] | HTN | Primary | SBP | mm Hg | -2.0 (CI: -4.9, 0.8)  *P* = 0.16 |
|  |  | Primary | DBP | mm Hg | - |
|  |  | Primary | Blood pressure <140/90 mm Hg | No. / % |  |
|  |  | Secondary | Self-reported full adherence to antihypertensive medications | No. |  |
|  |  | Secondary | Antihypertensive agents used | No. |  |
|  |  | Secondary | Antihypertensive medication intensification, additions, dose increases, or substitutions | No. |  |
|  |  | Secondary | Months when a home blood pressure reading is obtained | No. |  |
|  |  | Secondary | Frequency of home blood pressure measurements per month derived | median (interquartile range) |  |
|  |  | Secondary | Self-efficacy score | 10 questions |  |
|  |  | Secondary | BMI | kg/m^2^ |  |
|  |  | Secondary | Dietary Approaches to Stop Hypertension | Questionnaire score |  |
|  |  | Secondary | Consumption of processed meats | day/week |  |
|  |  | Secondary | Consumption of fried foods | day/week |  |
|  |  | Secondary | Consumption of sugar-sweetened beverages | day/week |  |
|  |  | Secondary | Consumption of candy, baked goods, or ice cream | day/week |  |
|  |  | Secondary | Self-reported physical activity (min/week of at least moderate exercise) | day/week |  |
|  |  | Secondary | Self-reported sleep duration | hour/night |  |
|  |  | Secondary | Health system contacts (telephone, office, and patient portal encounters) | No. |  |
| Quinn et al., 2011 [66] | T2DM | Primary | Change HbA1c* | % | - |
|  |  | Secondary | Depressive symptoms | PHQ |  |
|  |  | Secondary | Diabetes symptoms | Diabetes symptom inventory + Diabetes Distress Scale |  |
|  |  | Secondary | Hypoglycemic events | No. |  |
|  |  | Secondary | Hospitalization | No. |  |
|  |  | Secondary | Emergency room visits | No. |  |
|  |  | Secondary | SBP | mm Hg |  |
|  |  | Secondary | DBP | mm Hg |  |
|  |  | Secondary | LDL | mg/dL |  |
|  |  | Secondary | HDL | mg/dL |  |
|  |  | Secondary | TG | mg/dL |  |
|  |  | Secondary | TC | mg/dL |  |
| Quinn et al., 2014 [67] | T2DM | Primary | Medication prescription changes | n (%) | - |
| Quinn et al., 2016 [68] | T2DM | Primary | Change HbA1c* | % | -1.2 (CI:0.5, 1.9)  *P* = 0.001 |
|  |  | Secondary | Change HbA1c* in younger age groups (≥55)* | % | -1.0 CI: (-1.8, -0.2)  P = 0.02 |
|  |  | Secondary | Change HbA1c in older age groups (<55)* | % | -1.4 (CI =−2.3,  -0.6)^d^ *P* = .001 |
| Rossi et al., 2010 [89] | T1DM | Primary | HbA1c | % | - |
|  |  | Secondary | Change in FBG | mg/dL |  |
|  |  | Secondary | SBP | mm Hg |  |
|  |  | Secondary | DBP | mm Hg |  |
|  |  | Secondary | Change in body weight | kg |  |
|  |  | Secondary | TC | mg/dL |  |
|  |  | Secondary | LDL | mg/dL |  |
|  |  | Secondary | HDL | mg/dL |  |
|  |  | Secondary | TG* | mg/dL |  |
|  |  | Secondary | BP | mm Hg |  |
|  |  | Secondary | QoL (general health* and role emotional* parameters) | SF-36, DTSQ |  |
| Rossi et al., 2013 [90] | T1DM | Primary | HbA1c reduction | % | - |
|  |  | Secondary | Change in FBG | mg/dL | - |
|  |  | Secondary | Glucose variability | MAGE |  |
|  |  | Secondary | Mean daily doses of basal and prandial insulin | IU |  |
|  |  | Secondary | Frequency of hypoglycemic episodes | - |  |
|  |  | Secondary | Change in body weight | kg |  |
|  |  | Secondary | TC | mg/dL |  |
|  |  | Secondary | LDL | mg/dL |  |
|  |  | Secondary | HDL | mg/dL |  |
|  |  | Secondary | TG | mg/dL |  |
|  |  | Secondary | BP | mm Hg |  |
|  |  | Secondary | QoL (social relations* parameter) | DSQOLS |  |
| Sarfo et al., 2018 [50] | HTN + stroke survivors | Primary | SBP control defined as <140 mm Hg | % | - |
|  |  | Secondary | Medication adherence* | Medication possession ratio at month 3 |  |
|  |  | Secondary | Hypertension management competence | 18-item perceived confidence scale |  |
|  |  | Secondary | Autonomous self-regulation | 15-item treatment self-regulation questionnaire |  |
| Sarfo et al., 2019 [51] | HTN + stroke survivors | Primary | SBP control defined as <140 mm Hg (month 9)* | % | - |
|  |  | Primary | DBP control defined as <90 mm Hg (month 9)* | % |  |
|  |  | Secondary | Medication adherence (only medication possession ratio*) | Medication possession ratio + MMAS |  |
|  |  | Secondary | Hypertension management competence | 18-item perceived confidence scale |  |
|  |  | Secondary | Autonomous self-regulation | 15-item treatment self-regulation questionnaire |  |
|  |  | Secondary | Hypertension and stroke knowledge | 14-item questionnaire |  |
| Skrøvseth et al., 2015 [105] | T1DM | Primary | Hypo- and hyperglycemic events | No. | - |
|  |  | Secondary | Change in HbA1c | Out of range |  |
| Sun et al., 2019 [74] | T2DM | Primary | HbA1c* | % | - |
|  |  | Primary | PBG* | mmol/L |  |
|  |  | Primary | FBG | mmol/L |  |
|  |  | Primary | TC | mmol/L |  |
|  |  | Primary | TG | mmol/L |  |
|  |  | Primary | LDL | mmol/L |  |
|  |  | Primary | HDL | mmol/L |  |
|  |  | Primary | TG | mmol/L |  |
|  |  | Primary | BMI | kg/m2 |  |
|  |  | Primary | SBP | mm Hg |  |
|  |  | Primary | DBP | mm Hg |  |
| Torbjørnsen et al., 2014 [104] | T2DM | Primary | HbA1c | % | - |
|  |  | Secondary | Self-management | heiQ |  |
|  |  | Secondary | HRQoL | SF-36 |  |
| Waki et al., 2014 [117] | T2DM | Primary | Change in HbA1c* | % | - |
|  |  | Secondary | Change in FBG* | mg/dL |  |
|  |  | Secondary | BMI | kg/m^2^ |  |
|  |  | Secondary | SBP | mm Hg |  |
|  |  | Secondary | DBP | mm Hg |  |
|  |  | Secondary | TG | mg/dL |  |
|  |  | Secondary | Diabetes self-management | Diet and exercise |  |
| Wang et al., 2018 [69] | T2DM + overweight/obese | Primary | HbA1c | % | - |
|  |  | Secondary | Body weight | kg |  |
| Wang et al., 2019 [74] | T2DM | Primary | FBG* | mmol/L | - |
|  |  | Primary | 2-hour BG* | mmol/L |  |
|  |  | Primary | Self-management* | RAND |  |
|  |  | Primary | Disease awareness* | Questionnaire |  |
|  |  | Primary | Rehospitalization rate* | % |  |
| Wayne et al., 2015 [83] | T2DM | Primary | HbA1c (between group) | % and mmol/mol | - |
|  |  | Secondary | HbA1c (within group)* | % and mmol/mol |  |
|  |  | Secondary | Body weight | kg |  |
|  |  | Secondary | Waist circumference | cm |  |
|  |  | Secondary | BMI* | kg/m^2^ |  |
|  |  | Secondary | Life satisfaction | Satisfaction with Life Scale |  |
|  |  | Secondary | Anxiety and depression | HADS |  |
|  |  | Secondary | Positive affect | PANAS |  |
|  |  | Secondary | Negative affect* | PANAS |  |
|  |  | Secondary | QoL (mental composite*) | SF-12 |  |
| Yang et al., 2020 [93] | T2DM | Primary | Change in HbA1c* | % and mmol/mol | -0.30%  (CI: -0.50, -0.11%)  -3.32 mmol/mol  (CI: -5.50, -1.15 mmol/mol)  *P* = 0.003 |
|  |  | Secondary | Change in FBG* | mg/dL | −17.29 (−29.33 to −5.26)  *P* = 0.005 |
|  |  | Secondary | Body weight | kg |  |
|  |  | Secondary | Waist circumference | cm |  |
|  |  | Secondary | BMI | kg/m^2^ |  |
|  |  | Secondary | SBP* | mm Hg |  |
|  |  | Secondary | DBP* | mm Hg |  |
|  |  | Secondary | TC | mg/dL |  |
|  |  | Secondary | TG | mg/dL |  |
|  |  | Secondary | LDL | mg/dL |  |
|  |  | Secondary | HDL | mg/dL |  |
|  |  | Secondary | Diabetes treatment satisfaction* | DTSQs |  |
|  |  | Secondary | Motivation for long-term medication adherence/knowledge* | MMAS |  |
| Yu et al., 2019 [76] | T2DM | Primary | HbA1c change | % | - |
|  |  | Primary | Proportion of patients achieving HbA1c <7.0%* | % |  |
|  |  | Secondary | FPG | mmol/L |  |
|  |  | Secondary | 1,5-anhydroglucitol | µg/mL |  |
| Zha et al., 2020 [70] | HTN | Primary | SBP | mm Hg | - |
|  |  | Primary | DBP | mm Hg |  |
|  |  | Primary | BP monitoring adherence | % |  |
|  |  | Primary | Perceived medication adherence efficacy | MASES |  |
|  |  | Primary | HRQoL (mental health component*) | SF-36 |  |
| Zhai et al., 2020 [77] | T2DM | Primary | HbA1c* | % | - |
|  |  | dia | Self-efficacy* | DSES |  |
| Zhang et al., 2019 [78] | DM | Primary | HbA1c (3 and 6 months)* | % | - |
|  |  | Secondary | FPG* | mmol/L |  |
|  |  | Secondary | Body weight | kg |  |
|  |  | Secondary | LDL | mmol/L |  |
|  |  | Secondary | HDL* | mmol/L |  |
|  |  | Secondary | TG* | mmol/L |  |
|  |  | Secondary | TC | mmol/L |  |
| Zhou et al., 2016 [79] | DM | Primary | HbA1c* | mmol/L + % | - |
|  |  | Primary | FPG* | mmol/L |  |
|  |  | Primary | 2h-BG* | mmol/L |  |
|  |  | Primary | Body weight | kg |  |
|  |  | Primary | BMI | kg/m2 |  |
|  |  | Primary | Waist circumference | cm |  |
|  |  | Primary | Hip circumference | cm |  |
|  |  | Primary | SBP | mm Hg |  |
|  |  | Primary | DBP | mm Hg |  |
|  |  | Primary | Diabetes knowledge* | Diabetes knowledge score |  |
|  |  | Primary | Self-care behavior* | Self-care behavior score |  |
|  |  | Primary | Hypoglycemia events | event/patient month |  |
|  |  | Primary | LDL | mmol/L |  |

**Notes**: Reference numbers refer to the reference numbers in the main text.

Abbreviations: ADDQOL: app: application; Audit of Diabetes Dependent Quality of Life; ADS: Appraisal of diabetes scale; ASA24: Automated Self-Administered 24-hour; ASI: Anxiety Sensitivity Index; ASK-12: Adherence Starts with Knowledge-12; AQoL: Assessment of quality of life; BG: Blood glucose; BI: Basal insulin; BMI: Body mass index; BP: Blood pressure; CBT: cognitive behavioural therapy; CES-D: Center for Epidemiologic Studies Depression Scale; CESD: Centre for Epidemiologic Studies Short Depression scale; CI: confidence interval; DBP: Diastolic blood pressure; DFRQ: Diabetes Family Responsibility Questionnaire; DHP: Diabetes health profile; DID: Diabetes Interactive Diary; DKQ24: Diabetes Knowledge Questionnaire 24; DKT: Diabetes Knowledge Test; DM: diabetes mellitus; DMSES: Diabetes Management Self-efficacy Scale; DQI: Diet Quality Index; DSQoLS: Diabetes Specific Quality of Life Scale; DTSQs: Diabetes Treatment Satisfaction Questionnaire status; DQOLY: Diabetes Quality of Life for Youth Scale; EDI: Eating disorder inventory; EQ-5D: EuroQol-5D; FBG: fasting blood glucose; FPG: fasting plasma glucose; HADS: Hospital Anxiety and Depression Scale; HbA1c: glycated hemoglobin; HBPM: home blood pressure monitor; HCCQ: Health Care Climate Questionnaire; HDL-c: high density lipoprotein; heiQ: Health Education Impact Questionnaire; HPCP: hypertension personal control program; HRQoL: Health-related quality of life; HTN: Hypertension; IMDSES: insulin management diabetes self-efficacy scale; IMEVID: Instrument to Measure Lifestyle of Type 2 Diabetes Mellitus Patients; IPAQ-S: International physical activity questionnaire- short; IVRS: interactive voice response system; LDL-c: low density lipoprotein; MA: Medication adherence; MAGE: mean amplitude of glucose excursions; MASES: Medication Adherence Self-Efficacy Scale; MEDAS: Mediterranean Diet Adherence Screener; MMAS: Morisky medication adherence scale; MMS: Modified Morisky Scale; MTH: Mobile Telehealth; NA: Not applicable; PAID: Problem Areas in Diabetes; PAM: Patient Activation Measure; PANAS: Positive and Negative Affect Schedule; PBG: Plasma blood glucose; PCD: Perceived Competence in Diabetes Scale; PERC: Stanford Patient Education Research Center; PHQ: Patient Health Questionnaire; PDM: Personal models of diabetes; SBP: Systolic blood pressure; SCFLDS: Self-care Confidence in Following a Low-sodium Diet Scale; SDSCA: Summary of Diabetes Self-Care Activities; SF: Short-Form Health Survey; SMBG: self-monitored blood glucose; SMPG: Self-monitoring plasma glucose; STAI: Short Trait Anxiety Inventory; T1DM: Type 1 diabetes mellitus; T2DM: Type 2 diabetes mellitus; TC: total cholesterol; TG: triglycerides; QoL: Quality of life.

* Statistically significant between group results.

^a^ Only health-related effectiveness/efficacy outcomes.

^b^ For laboratory and anthropometric parameters, the units of measurement was used. For self-reported outcomes, the questionnaires used without reporting the type of scoring of the questionnaire.

^c^ Effect estimates were only extracted for outcomes that had subgroup analyses with PROGRESS-Plus in objective 2, and the extracted numbers were the mean change between the intervention and the control groups.

^d^ The study Quinn et al., 2016 had a typo in the confidence interval reported in the article, but the typo is corrected in the table above.
