# Supplementary material for: Consideration of inequalities in effectiveness trials of mHealth applications – a systematic assessment of studies from an umbrella review
Source: Int J Equity Health. 2024 Sep 11;23:181. doi: 10.1186/s12939-024-02267-4 (PMC11389088; doi:10.1186/s12939-024-02267-4)
Supplement: Supplementary file 10 — Supplementary Material 10 [file 12939_2024_2267_MOESM10_ESM.docx]

**Additional File 10. Risk of bias assessment results**

**Individual Parallel RCTs**


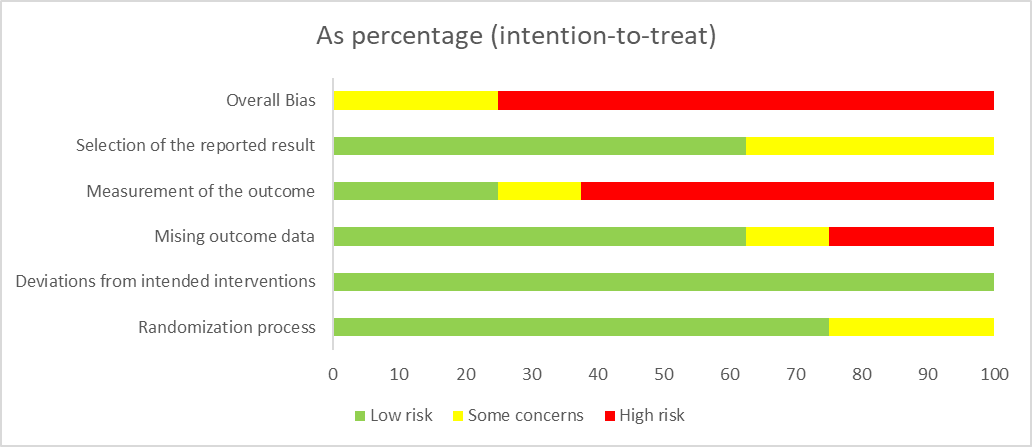


Figure 1: Weighted bar plots of the distribution of risk-of-bias judgements in each bias domain

Figure 2: Traffic light plot of the domain-level judgements for each outcome

**Notes**: Unique ID is the sequence number of the assessed outcome for risk of bias. The study ID is the reference number from the main text.

Abbreviations: DID: Diabetes Interactive Diary; HbA1c: glycated hemoglobin; HRQoL: Health-related quality of life; SBP: systolic blood pressure.

**Cluster RCTs**


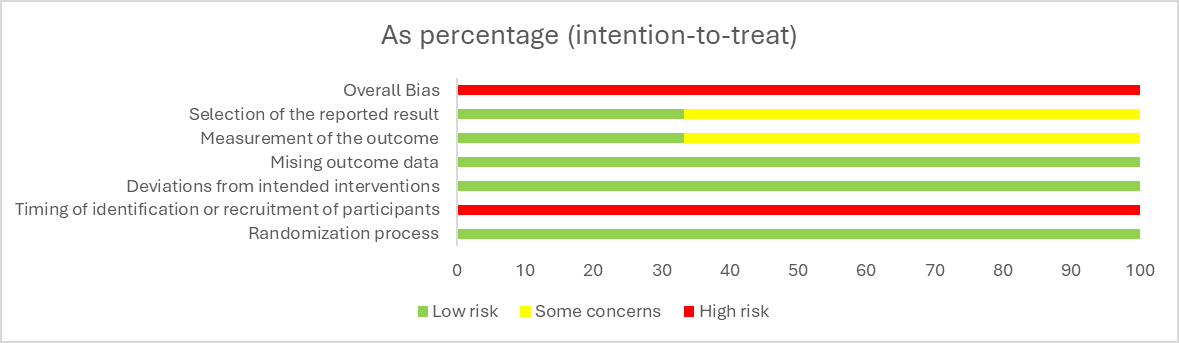


Figure 3: Weighted bar plots of the distribution of risk-of-bias judgements in each bias domain

Figure 4: Traffic light plot of the domain-level judgements for each outcome

**Notes**: Unique ID is the sequence number of the assessed outcome for risk of bias. The study ID is the reference number from the main text.

Abbreviations: CPDS: coach primary care provider portal with decision support; EUC: enhanced usual care; FPG: Fasting plasma glucose; HbA1c: glycated hemoglobin; SBP: systolic blood pressure; UC: usual care
