# Supplementary material for: Consideration of inequalities in effectiveness trials of mHealth applications – a systematic assessment of studies from an umbrella review
Source: Int J Equity Health. 2024 Sep 11;23:181. doi: 10.1186/s12939-024-02267-4 (PMC11389088; doi:10.1186/s12939-024-02267-4)
Supplement: Supplementary file 11 — Supplementary Material 11 [file 12939_2024_2267_MOESM11_ESM.docx]

Additional File 11. Details on Reported PROGRESS-Plus

**PROGRESS-Plus: Ethnicity**

Table 1: Percentages of ethnicities and racial backgrounds reported in the studies

| **Primary Author, Year** | **Country** | **Ethnicity/Race: Majority** | **Ethnicity/Race:  Minority** | **Ethnicity/Race: Other** |
| --- | --- | --- | --- | --- |
| Agarwal et al., 2019 [80] | Canada | Caucasian: 43% | Non-Caucasian: 56% | Refuse to answer: 1%; Missing: 1% |
| Baron et al., 2017 [85, 86] | United Kingdom | White: 25% | Asian: 36%;  Black: 33%;  Other: 6% | - |
| Bender et al., 2017 [55] | United States | - | Ethnicity: Filipino: 100% | - |
| Boels et al., 2019 [108] | Netherlands | Caucasian: 82% | 18%^a^ | - |
| Chandler et al., 2019 [56] | United States | - | Hispanic: 100% | - |
| Dorsch et al., 2020 [57] | United States | - Race: Caucasian: 80% - Ethnicity: non-Latino: 98% | - Race: African American: 10%;  Asian: 6%; other: 4% - Ethnicity: Latino: 2% | - |
| Dugas et al., 2018 [58] | United States | - | African American: 10% | - |
| Frias et al., 2017 [59] | United States | - Caucasian: 66% | - African American: 16%; Asian: 14% - Hispanic: 47%^b^ | - |
| Gong, E et al., 2020 [110] | Australia | - | Aboriginal or Torres Strait Islander origin: 2% | - |
| Hilliard., 2020 [61] | US | - Non-Hispanic white (61%) | - Non-Hispanic black: 13% - Hispanic: 19% - Other or more than one: 7% | - |
| Huang et al., 2019 [100] | Singapore | Chinese: 54% | Non-Chinese: 46% | - |
| Istepanian et al., 2009 [87] | United Kingdom | Caucasian: 34% | African-Caribbean: 31%;  Indo-Asian: 31%; Other: 4% | - |
| Lakshminarayan et al., 2018 [63] | United States | White: 91% | 9%^a^ | - |
| Logan et al., 2012 [82] | Canada | White/European: 65% | African/West Indian: 16%;  Asian: 10%;  Hispanics: 4%; Others: 5% | - |
| Morawski et al., 2018 [64] | United States | Race/Ethnicity: White: 65% | Race/Ethnicity: African American: 25%; other: 10% | - |
| Nagrebetsky et al., 2013 [88] | United Kingdom | White: 100% | - | - |
| Persell et al., 2020 [65] | United States | - Race: White: 52% | - Race: African American: 35%;  Asian: 4%; other or unknown: 9% - Ethnicity: Hispanic: 8% | - |
| Quinn et al., 2011 [66] | United States | Race: White (non-Hispanic): 53% | Race: African American (non-Hispanic): 39%; other: 8% | - |
| Quinn et al., 2014 [67] | United States | Race: White (non-Hispanic): 55% | Race: African American (non-Hispanic): 36%; other: 8% | - |
| Quinn et al., 2016 [68] | United States | Race: White (non-Hispanic): 55% | Race: African American (non-Hispanic): 37%; other: 8% | - |
| Wang et al., 2018 [69] | United States | - Race: White: 27% - Ethnicity: non-Hispanic: 77% | - Race: Black: 65%; American Indian: 4%;  Asian: 4% - Ethnicity: Hispanic: 23% | - |
| Wayne et al., 2015 [83] | Canada | Ethnicity: Caucasian: 27% | Ethnicity: Black (Caribbean): 40%; Hispanic: 9%;  West Indian: 6%;  Black (African): 5%;  South Asian: 4%;  Southeast Asian: 4%;  First Nations: 1%; other: 3% | - |
| Zhai et al., 2020 [77] | United States | - Race: White: 4% - Ethnicity: non-Hispanic: 96% | - Race: Black/African American: 96% - Ethnicity: Hispanic: 4% | - |
| *Minimum* | | 0% | 0% | 1.4% |
| *Maximum* | | 100% | 100% | 1.4% |
| *Mean* | | 54% | 45% | 1.4% |
| *Median* | | 55% | 45% | 1.4% |

**Notes**: Whenever the term *ethnicity* or *race* was used by the authors, the same term was used in the table. Percentages might not add up to 100% due to approximation of decimal numbers. Reference numbers refer to the reference numbers in the main text.

For Dorsch et al (2020), Frias et al (2017), Persell et al (2020), Wang et al (2018), and Zha et al (2020) race is what is included in the calculations of the minimum, maximum, mean and median.

^a^ Percentage of the remainder; not reported by the study but deduced from subtracting the other categories from 100%.
^b^ Reported separately as a percentage of the whole population.

**PROGRESS-Plus: Occupation**

Table 2. Percentages of reported occupational statuses of the participants

| **Primary Author, Year** | **Employed** | **Retired/Disabled** | **Unemployed** | **Other** | **Unclear/not reported** |
| --- | --- | --- | --- | --- | --- |
| Alonso-Dominguez et al., 2019 [94, 95] | Works outside home: 34% | Retired: 38% | Unemployed: 6% | Homemaker: 21% | - |
| Bender et al., 2017 [55] | Full/part time: 69% | Retired: 8% | Unemployed: 4% | - | 19% |
| Chandler et al., 2019 [56] | Full/part-time: 56% | Retired/disabled: 0% | Unemployed: 44% | - | - |
| Chao et al., 2019 [71] | Public servant: 4%;  Office worker: 18%; Businessman: 14% | Retired: 53% | Unemployed: 7% | Others: 4% | - |
| Di Bartolo et al., 2017 [97] | Employed: 13% | - | Unemployed: 14% | Student: 74% | - |
| Frias et al., 2017 [59] | Employed: 47% | - | - | - | 53% |
| Gong, E et al., 2020 [110] | Full time: 47%;  Part time or casual: 16% | Retired: 23% | Unemployed or others: 14% | |  |
| Holmen et al., 2014 [103] | Employed: 52% | Retired: 19% | Unemployed: 27% | - | 2% |
| Kusnanto et al., 2019 [49] | Employed: 83% | - | Not working: 17% | - | - |
| Lee et al., 2017 [53] | Employed: 52% | - | Unemployed: 47% | - | 1% |
| Lee et al., 2020 [92] | Full-time: 68% | - | - | - | 32% |
| Sarfo et al., 2019 [51] | Employed: 73% | Retired: 17% | Unemployed: 10% | - | - |
| Wang et al., 2018 [69] | Full time: 12%;  Part time: 15% | Retired: 19%; Disable/unable to work: 31% | Laid-off: 8% | Full time homemaker: 12%;  Student: 4% | - |
| Wayne et al., 2015 [83] | Part time: 6%;  Full time: 26%;  Self-employed: 9% | Retired: 11% | Unemployed: 36% | Student: 4%; Work in home: 4%;  Not disclosed: 3% | - |
| Zhang et al., 2019 [78] | Working: 58% | - | - | - | 42% |
| *Minimum* | 13% | 0% | 4% | 4% | 0% |
| *Maximum* | 83% | 53% | 47% | 74% | 53% |
| *Mean* | 51% | 24% | 20% | 25% | 10% |
| *Median* | 52% | 19% | 14% | 16% | 0% |

**Notes**: Percentages might not add up to 100% due to approximation of decimal numbers. Reference numbers refer to the reference numbers in the main text. Unclear/unreported is calculated by subtracting the reported occupation from 100 whenever applicable.

**PROGRESS-Plus: Gender**

Table 3: Gender of the Participants in the Studies

| **Primary Author, Year** | **Males** | **Females** |
| --- | --- | --- |
| Agarwal et al., 2019 [80] | 52% | 48% |
| Alanzi et al., 2018 [106] | 75% | 25% |
| Alfonsi et al., 2020 [81] | 61% | 39% |
| Alonso-Dominguez et al., 2019 [94, 95] | 54% | 46% |
| Anzaldo-Campos et al., 2016 [52] | 33% | 67% |
| Baron et al., 2017 [85, 86] | 57% | 43% |
| Bee et al., 2016 [99] | 67% | 33% |
| Bender et al., 2017 [55] | 38% | 62% |
| Berndt et al., 2014 [115] | 60% | 40% |
| Boels et al., 2019 [108] | 60% | 40% |
| Brath et al., 2013 [114] | 56% | 44% |
| Castelnuovo et al., 2011 [98] | 52% | 48% |
| Castensøe-Seidenfaden et al., 2018 [118] | 46% | 54% |
| Chandler et al., 2019 [56] | 34% | 66% |
| Charpentier et al., 2011 [101] | 37% | 63% |
| Chatzakis et al., 2019 [119] | 49% | 51% |
| Di Bartolo et al., 2017 [97] | 51% | 49% |
| Dorsch et al., 2020 [57] | 40% | 60% |
| Drion et al., 2015 [109] | 63% | 37% |
| Dugas et al., 2018 [58] | 90% | 10% |
| Franc et al., 2019 [102] | 65% | 35% |
| Frias et al., 2017 [59] | 50% | 50% |
| Garg et al., 2017 [60] | 52% | 48% |
| Gong, E et al., 2020 [110] | 58% | 42% |
| Gong, K et al., 2020 [72] | 55% | 45% |
| Goyal et al., 2017 [84] | 45% | 55% |
| Gunawardena et al., 2019 [47] | 60% | 40% |
| Hilliard., 2020 [61] | 41% | 59% |
| Holmen et al., 2014 [103] & Torbjørnsen et al., 2014 [104] | 59% | 41% |
| Huang et al., 2019 [100] | 49% | 51% |
| Kardas et al., 2016 [116] | 60% | 40% |
| Kim et al., 2019 [91] | 49% | 51% |
| Kirwan et al., 2013 [111] | 39% | 61% |
| Klee et al., 2018 [113] | 56% | 44% |
| Kleinman et al., 2017 [48] | 70% | 30% |
| Kusnanto et al., 2019 [49] | 43% | 57% |
| Lakshminarayan et al., 2018 [63] | 68% | 32% |
| Lee et al., 2017 [53] | 46% | 54% |
| Lee et al., 2020 [92] | 73% | 27% |
| Logan et al., 2012 [82] | 56% | 45% |
| Márquez Contreras et al., 2019 [96] | 48% | 52% |
| Morawski et al., 2018 [64] | 40% | 60% |
| Nagrebetsky et al., 2013 [88] | 71% | 29% |
| Or et al., 2016 [73] | 32% | 68% |
| Orsama et al., 2013 [112] | 54% | 46% |
| Persell et al., 2020 [65] | 39% | 61% |
| Quinn et al., 2011 [66] | 50% | 50% |
| Quinn et al., 2014 [67] | 50% | 49% |
| Quinn et al., 2016 [68] | 50% | 50% |
| Rossi et al., 2010 [89] | 43% | 57% |
| Rossi et al., 2013 [90] | 48% | 52% |
| Sarfo et al., 2019 [50, 51] | 65% | 35% |
| Skrøvseth et al., 2015 [105] | 37% | 63% |
| Sun et al., 2019 [74] | 41% | 59% |
| Waki et al., 2014 [117] | 76% | 24% |
| Wang et al., 2018 [69] | 42% | 58% |
| Wang et al., 2019 [74] | 53% | 47% |
| Wayne et al., 2015 [83] | 28% | 72% |
| Yang et al., 2020 [93] | 51% | 49% |
| Yu et al., 2019 [76] | 38% | 62% |
| Zha et al., 2020 [70] | 12% | 88% |
| Zhai et al., 2020 [77] | 49% | 51% |
| Zhang et al., 2019 [78] | 62% | 38% |
| Zhou et al., 2016 [79] | 57% | 43% |
| *Minimum* | 12% | 10% |
| *Maximum* | 90% | 88% |
| *Mean* | 52% | 48% |
| *Median* | 51% | 49% |

**Notes**: Percentages might not add up to 100% due to approximation of decimal numbers. Reference numbers refer to the reference numbers in the main text.

**PROGRESS-Plus: Education**

*Table 4: Reported Educational Levels of the Participants in the Studies*

| **Primary Author, Year** | **Low Education** | | **Intermediate Education** | | **High Education** | | **Other** |
| --- | --- | --- | --- | --- | --- | --- | --- |
|  | **Elementary/ Primary Education** | **Middle School** | **Secondary/ High School Education** | **Diploma/ Vocational Education** | **University Education** | **Graduate/ Post-Graduate Education** |  |
| Agarwal et al., 2019 [80] | High school or less: 31% | | | College degree or diploma: 42% | Undergraduate university degree: 11% | Postgraduate degree: 5% | Other: 5%;  Not applicable: 1%;  Refuse to answer: 4%;  Missing: 1% |
| Alanzi et al., 2018 [106] | ≤ Secondary: 25% | | | Diploma: 35% | ≥ University: 40% | - | - |
| Alonso-Dominguez et al., 2019 [94, 95] | Elementary school: 49% | Middle or high school: 34% | | - | University studies: 17% | - | - |
| Anzaldo-Campos et al., 2016 [52] | Basic (till 6^th^ grade): 52% | Middle: 43% | College and above (13^th^ + grade): 5% | | | - | - |
| Baron et al., 2017 [85, 86] | GCSE/O’ levels: 33%  - | | A-level/HNC: 11% | - | University level: 12% | Graduate/ Professional: 11% | No formal education: 32% |
| Bender et al., 2017 [55] | - | - | - | - | College 1-4 years: 80% | Graduate school: 20% | - |
| Boels et al., 2019 [108] | Low: 9% | | Middle: 55% | | High: 36% | | - |
| Castensøe-Seidenfaden et al., 2018 [118] | Danish public school (grade 0-10): 36% | Continuation school: 3% | Secondary education: 19% | - | University: 9% | Other schools: 15% | Not attending a school: 19% |
| Chandler et al., 2019 [56] | ≤ High School: 72% | | | - | Partial/College Grad: 28% | - | - |
| Chao et al., 2019 [71] | - | - | High school education: 75% | - | - | - | - |
| Charpentier et al., 2011 [101] | Low level (≤ college): 24% | | Intermediate level (≤ university degree): 21% | | High level (university degree): 55% | - | - |
| Di Bartolo et al., 2017 [97] | Primary school or middle school: 33% | | High school: 60% | | Graduate/post-graduate: 5% | | - |
| Drion et al., 2015 [109] | Primary school: 3% | Low level: 2% + Intermediate level: 36% | High school: 14% | - | University: 45% | - | - |
| Frias et al., 2017 [59] | Less than high school: 31% | | - | - | - | - | - |
| Gong, E et al., 2020 [110] | ≤ Secondary high school: 19% | | | Technical apprenticeship or diploma: 31% | Bachelor’s degree: 21% | ≥ Postgraduate degree: 19% | - |
| Gunawardena et al., 2019 [47] | - | ≤ High school education: 67% | - | - | - | - | - |
| Holmen et al., 2014 [103] & Torbjørnsen et al., 2014 [104] | - | Some high school or less (<12 years): 55% | High school graduate (12 years): 11% | - | Some college or more (>12 years): 34% | - | - |
| Huang et al., 2019 [100] | ≤ Secondary school: 41% | | | Junior college/diploma: 22% | University: 37% | - | - |
| Kleinman et al., 2017 [48] | - | < High school: 29% | High school graduate: 46% | - | ≥ University: 26% | - | - |
| Kusnanto et al., 2019 [49] | - | - | High school: 60% | - | College: 40% | - | - |
| Lakshminarayan et al., 2018 [63] | ≤ High School: 20% | | | Some college: 38% | College: 27% | Graduate: 13% | Other: 4% |
| Lee et al., 2017 [53] | Primary: 29% | - | Secondary: 61% | - | Tertiary: 5% | - | Illiterate/no formal education: 5% |
| Lee et al., 2020 [9] | - | - | ≥ High school: 92% | | | | - |
| Morawski et al., 2018 [64] | - | Did not finish high school: 2% | High school graduate: 12% | Some college degree: 25% + vocational: 10% | College graduate: 34% | Graduate degree: 17% | - |
| Or et al., 2016 [73] | Primary: 57% | - | Secondary: 35% | - | ≥ College: 8% | - | - |
| Orsama et al., 2013 [112] | Average number of years of education: 12 | | | | | | |
| Persell et al., 2020 [65] | - | < High school graduate: 2% | Grade 12 or General Education Development examination: 9% | - | College 1-3 years: 19% + College ≥4 years: 71% | - | Unknown: 0% |
| Quinn et al., 2011 [66] | ≤ High school/trade school: 30% | | | Some college or associates: 39% | ≥ Bachelor’s degree: 31% | - | - |
| Quinn et al., 2014 [67] | ≤ High school/trade school: 27% | | | Some college or associates: 36% | ≥ Bachelor’s degree: 36% | - | - |
| Quinn et al., 2016 [68] | ≤ High school/trade school: 28% | | | Some college or associates: 36% | ≥ Bachelor’s degree: 36% | - | - |
| Rossi et al., 2010 [89] | - | Low level (< college degree): 18% | - | Intermediate level (< university degree): 64% | High level (university degree): 18% | - | - |
| Rossi et al., 2013 [90] | - | Low level (< college degree): 15% | - | Intermediate level (< university degree): 61% | High level (university degree): 24% | - | - |
| Sarfo al., 2019 [50, 51] | Primary: 47% | - | Secondary: 30% | - | Tertiary: 18% | - | No education: 5% |
| Wang et al., 2018 [69] | Average number of years of education: 12 | | | | | | |
| Wang et al., 2019 [74] | ≤ High school: 50% | | | Junior college: 35% | ≥ Bachelor's degree: 15% | - | - |
| Wayne et al., 2015 [83] | - | < High school: 23% | High school diploma: 36% | College or vocational training: 26% | University degree: 12% |  | Not disclosed: 3% |
| Zhai et al., 2020 [77] | - | - | ≥ High school: 19% | | | | - |
| Zhang et al., 2019 [78] | ≤ Primary school: 20% | | High school: 34% | ≥ Junior college: 46% | | | - |

**Notes**: Percentages might not add up to 100% due to approximation of decimal numbers. Reference numbers refer to the reference numbers in the main text.

Abbreviations: GCSE: General Certificate of Secondary Education; HNC: higher national certificate.

**PROGRESS-Plus: Social Capital**

Table 5: Reported Social Capital of the Participants in the Studies.

| **Primary Author, Year** | **Social Capital: Married/Cohabiting** | **Social Capital: Single/Separated/ Divorced/Widowed/Never married** | **Other/unclear/ unknown/not reported** |
| --- | --- | --- | --- |
| Alanzi et al., 2018 [106] | Married: 70% | Never married: 30% | - |
| Anzaldo-Campos et al., 2016 [52] | Married or domestic partnership: 67% | Single/divorced/widow: 33% | - |
| Bender et al., 2017 [55] | Married/cohabitating: 67% | Divorced/widowed: 22%; Never married: 11% | - |
| Chandler et al., 2019 [56] | Married/Living with Significant Other: 55%; | Separated/Divorced: 22%; Single: 17%; Widowed: 6% | - |
| Di Bartolo et al., 2017 [97] | Living with family: 96% | Living alone: 3% | Other: 1% |
| Holmen et al., 2014 [103] | Cohabitation status (cohabiting/married/living with someone): 73% | - | Unclear/not reported: 27% |
| Lakshminarayan et al., 2018 [63] | Married: 71% | Single: 25% | Other/unknown: 4% |
| Lee et al., 2017 [53] | Married: 92% | Divorced: 6%;  Widower: 2% | - |
| Or et al., 2016 [73] | Live with families: 75% | Live alone: 25% | - |
| *Minimum* | 55% | 3% | 0% |
| *Maximum* | 96% | 45% | 38% |
| *Mean* | 73% | 25% | 7% |
| *Median* | 71% | 28% | 0% |

**Notes**: Percentages might not add up to 100% due to approximation of decimal numbers. Reference numbers refer to the reference numbers in the main text.

**PROGRESS-Plus: Socioeconomic Position (SEP) - Income**

Table 6: Reported Income of the Participants in the Studies

| **Primary Author, Year** | **Country** | **Currency** | **Percentages and Categories of Income** |
| --- | --- | --- | --- |
| Agarwal et al., 2019 [80] | Canada | Can$ | <35000: 24%;  35000 - 50000: 15%;  >50000 - 80000: 18%;  >80000 - 150000: 17%;  >150000: 5%;  Not applicable: 6%; Refuse to answer: 14%; Missing: 1% |
| Chandler et al., 2019 [56] | United States | Income US$ | 0 - 25000: 66%;  25 - 50000: 25%;  >50000: 6%;  Not Reported: 7% |
| Frias et al., 2017 [59] | United States | Income US$ | ≤20000: 57% |
| Huang et al., 2019 [100] | Singapore | Income US$ | <4000: 37%;  4000 - 6999: 24%;  ≥7000: 34% |
| Huang et al., 2019 [100] | Singapore | Housing (number of rooms) | ≤3: 15%;  4 - 5: 54%;  ≥5: 32% |
| Sarfo et al., 2019 [51] | Ghana | Income US$/month | 0 - 100: 33%;  101 - 250: 40%;  251 - 500: 15%;  >501: 12% |
| Wang et al., 2018 [69] | United States | Income US$ | <10000: 23%;  10000 - 13000: 23%;  13000 - 20000: 42%;  20000 - 30000: 8% |
| Wayne et al., 2015 [83] | Canada | Can$ | 0 - 9999: 22%;  10000 - 25,000: 24%;  25000 - 50000: 21%;  50000 - 75000: 9%;  ≥75000: 5%;  Not disclosed: 20% |
| Wayne et al., 2015 [83] | Canada | Car access | Owns a car: 36%;  Has access to car: 12%;  No access to car: 50%;  Not disclosed: 2% |

**Notes**: Percentages might not add up to 100% due to approximation of decimal numbers. Reference numbers refer to the reference numbers in the main text.

**PROGRESS-Plus: Age**

Table 7: Reported Age of the Participants in the Studies

| **Primary Author, Year** | **Age Range/Median Age/ Weighted Mean Age in Years** | **Disease** |
| --- | --- | --- |
| Agarwal et al., 2019 [80] | 51.8 | Type 2 diabetes |
| Alanzi et al., 2018 [106] | 18-40: 85%;  41-50: 15% | Type 2 diabetes |
| Alfonsi et al., 2020 [81] | 13.98 | Type 1 diabetes |
| Alonso-Dominguez et al., 2019 [94, 95] | 60.6 | Type 2 diabetes |
| Alotaibi et al., 2016 [107] | 45.15 | Type 2 diabetes |
| Anzaldo-Campos et al., 2016 [52] | 51.54 | Type 2 diabetes |
| Baron et al., 2017 [85, 86] | 57.13 | Type 1 and/or 2 diabetes |
| Bee et al., 2016 [99] | 53.3 | Type 2 diabetes |
| Bender et al., 2017 [55] | 57.6 | Type 2 diabetes |
| Berndt et al., 2014 [115] | 13.05 | Type 1 diabetes |
| Boels et al., 2019 [108] | 59.15 | Type 2 diabetes |
| Brath et al., 2013 [114] | 69.4 | Type 2 diabetes/ hypertension |
| Castelnuovo et al., 2011 [98] | 51.5 (median) | Type 2 diabetes |
| Castensøe-Seidenfaden et al., 2018 [118] | 17.6 | Type 1 diabetes |
| Chandler et al., 2019 [56] | 45.65 | Hypertension |
| Charpentier et al., 2011 [101] | 33.8 | Type 1 diabetes |
| Chatzakis et al., 2019 [119] | 13.5 | Type 1 diabetes |
| Di Bartolo et al., 2017 [97] | 17.7 | Type 1 diabetes |
| Dorsch et al., 2020 [57] | 57.43 | Hypertension |
| Drion et al., 2015 [109] | 33 (median) | Type 1 diabetes |
| Dugas et al., 2018 [58] | 67.56 | Type 2 diabetes |
| Franc et al., 2019 [102] | 58.7 | Type 2 diabetes |
| Frias et al., 2017 [59] | 58.81 | Hypertension/type 2 diabetes |
| Garg et al., 2017 [60] | 38.5 | Type 1 diabetes |
| Gong, E et al., 2020 [110] | 56.9 | Type 2 diabetes |
| Gong, K et al., 2020 [72] | 58.73 | Hypertension |
| Goyal et al., 2017 [84] | 14 | Type 1 diabetes |
| Gunawardena et al., 2019 [47] | 52.48 | Type 1 and/or 2 diabetes |
| Hilliard., 2020 [61] | 15.3 | Type 1 diabetes |
| Holmen et al., 2014 [103] & Torbjørnsen et al., 2014 [104] | 57.31 | Type 2 diabetes |
| Hsu et al., 2016 [62] | 53.55 | Type 2 diabetes |
| Huang et al., 2019 [100] | 51.73 (median) | Type 2 diabetes |
| Istepanian et al., 2009 [87] | 58.58 | Type 1 and/or 2 diabetes |
| Kardas et al., 2016 [116] | 59.45 | Type 2 diabetes |
| Kim et al., 2019 [91] | 58.43 | Type 2 diabetes |
| Kirwan et al., 2013 [111] | 35.2 | Type 1 diabetes |
| Klee et al., 2018 [113] | 13.6 | Type 1 diabetes |
| Kleinman et al., 2017 [48] | 48.39 | Type 2 diabetes |
| Kusnanto et al., 2019 [49] | 36-45: 13%;  46-55: 53%;  56-65: 33% | Type 2 diabetes |
| Lakshminarayan et al., 2018 [63] | 65.03 | Hypertension + stroke survivors |
| Lee et al., 2017 [53] | 53.49 | Type 2 diabetes |
| Lee et al., 2020 [92] | 51.31 | Type 2 diabetes |
| Logan et al., 2012 [82] | 62.9 | Diabetes + hypertension |
| Márquez Contreras et al., 2019 [96] | 57.39 | Hypertension |
| Morawski et al., 2018 [64] | 52.04 | Hypertension |
| Nagrebetsky et al., 2013 [88] | 58 | Type 2 diabetes |
| Or et al., 2016 [73] | 69.5 | Hypertension/type 2 diabetes |
| Orsama et al., 2013 [112] | 61.9 | Type 2 diabetes |
| Persell et al., 2020 [65] | 58.93 | Hypertension |
| Quinn et al., 2011 [66] | 52.75 | Type 2 diabetes |
| Quinn et al., 2014 [67] | 52.62 | Type 2 diabetes |
| Quinn et al., 2016 [68] | 52.59 | Type 2 diabetes |
| Rossi et al., 2010 [89] | 35.74 | Type 1 diabetes |
| Rossi et al., 2013 [90] | 36.33 | Type 1 diabetes |
| Sarfo et al., 2018 [50, 51] | 55.1 | Hypertension + stroke survivors |
| Skrøvseth et al., 2015 [105] | 39.7 | Type 1 diabetes |
| Sun et al., 2019 [74] | 67.98 (median) | Type 2 diabetes |
| Waki et al., 2014 [117] | 57.3 | Type 2 diabetes |
| Wang et al., 2018 [69] | 55.65 | Type 2 diabetes + overweight/obese |
| Wang et al., 2019 [74] | 45.4 | Type 2 diabetes |
| Wayne et al., 2015 [83] | 53.2 | Type 2 diabetes |
| Yang et al., 2020 [93] | 56.65 | Type 2 diabetes |
| Yu et al., 2019 [76] | 52.53 | Type 2 diabetes |
| Zha et al., 2020 [70] | 52.33 | Hypertension |
| Zhai et al., 2020 [77] | 55.64 | Type 2 diabetes |
| Zhang et al., 2019 [78] | 53 | Type 1 and/or 2 diabetes |
| Zhou et al., 2016 [79] | 54.25 | Type 1 and/or 2 diabetes |
| *Minimum* | 13.05 |  |
| *Maximum* | 69.5 |  |
| *Mean* | 48.9 |  |
| *Median* | 53.2 |  |

**Notes**: Reference numbers refer to the reference numbers in the main text
